# Impaired Humoral Immunity Identified in Inactivated SARS-CoV-2 Vaccine Recipients without Anti-Spike RBD Antibodies

**DOI:** 10.1128/spectrum.02783-22

**Published:** 2023-03-14

**Authors:** Ting Sun, Yuchen Wang, Xiaoyu Song, Ruili Li, Fanghua Mei, Mengliu Yang, Xiaojie Huang, Yan Li, Xuwei Zhou, Haoyu Wang, Wendong Li, Jing Li, Lu Wang, Wei Shi, Kun Cai, Hongjun Li, Jing Zhang

**Affiliations:** a Key Laboratory for Biomechanics and Mechanobiology of the Ministry of Education, Beijing Advanced Innovation Centre for Biomedical Engineering, School of Engineering Medicine, Beihang University, Beijing, China; b Department of Radiology and Nuclear Medicine, Xuanwu Hospital, Capital Medical University, Beijing, China; c Hubei Center for Disease Control and Prevention, Wuhan, China; d Clinical and Research Center for Infectious Diseases, Beijing You An Hospital, Capital Medical University, Beijing, China; e Department of Pathogen Biology, School of Basic Medicine, Tongji Medical College, Huazhong University of Science and Technology, Wuhan, Hubei, China; f Department of Radiology, Beijing You An Hospital, Capital Medical University, Beijing, China; University of Georgia

**Keywords:** anti-Spike RBD antibodies, impaired humoral immunity, inactivated SARS-CoV-2 vaccine

## Abstract

Inactivated SARS-CoV-2 vaccines have been deployed in a significant portion of the world population, who have widely varied responses to vaccination. Understanding this differential response would help the development of new vaccines for non-responders. Here, we conducted surveillance of anti-Spike receptor-binding domain (RBD) antibody levels in a large cohort of 534 healthy Chinese subjects vaccinated with two doses of inactivated SARS-CoV-2 vaccines. We show that the positive rate of antibodies among vaccinated subjects rapidly wanes as the interval between antibody testing and vaccination increases (14 to 119 days: 81.03%, 363 of 448 subjects; 120 to 149 days: 46.43%, 13 of 28 subjects; more than 150 days: 20%, 1 of 5 subjects). However, the antibodies were maintained at high levels in 16 convalescent COVID-19 patients at more than 150 days after recovery. We also found that increased age and body mass index are associated with decreased antibody levels. Vaccinated subjects who fail to produce antibodies display impaired B-cell activating humoral immunity, which was confirmed in COVID-19 patients without antibodies detected at 4 to 18 days after diagnosis.

**IMPORTANCE** Our study illustrates the immune responses engaged by encountering antigen, highlighting the critical roles of B-cell activating humoral immunity in the body’s antibody production.

## INTRODUCTION

As the SARS-CoV-2 pandemic persists globally, vaccines are the most cost-effective way of preventing SARS-CoV-2 infection (1). More vaccines against SARS-CoV-2 are needed to mitigate the tragic health and socioeconomic consequences of the pandemic (2). Due to the unprecedentedly great efforts worldwide to develop vaccines against SARS-CoV-2, many COVID-19 vaccines have been proven to be effective by inducing humoral and cellular immune responses against SARS-CoV-2 (3–7). mRNA-based vaccines have been preferentially employed in the United States and Europe. Some inactivated vaccines, Sinovac CoronoVac and Sinopharm BBIBP-CorV, have been administered to a large portion of the world population, achieving 65 to 85% and 65 to 80% efficacy, respectively (4, 8–10). Sinovac CoronoVac and Sinopharm BBIBP-CorV vaccines have also been approved and administered at a nation-wide level in China to contain the pandemic. While clinical trial data appear promising, real-life evaluations are critical for comprehensive understanding of their effects on recipients’ immune systems and the influences of other biological and socioeconomic factors. Informing vaccine policy decisions associated with timing and booster doses for target populations also requires knowledge of the duration of antibody maintenance and to what extent antibody levels wane over time after vaccination.

To address the issues associated with antibody maintenance and immune response after vaccination, we examined the serologic receptor-binding domain (RBD)-specific IgG antibody levels of 534 healthy Chinese subjects aged 18 to 75 years who had received two doses of inactivated SARS-CoV-2 vaccines. We determined whether biological and socioeconomic components such as age and body mass index (BMI) imposed any influence on antibody levels and the duration of antibody maintenance. Based on these studies, we compared the antibody levels and antibody durations in sera from people who had received inactivated SARS-CoV-2 vaccines and convalescent-phase sera from subjects infected with SARS-CoV-2. We further investigated the potential molecular mechanisms at the transcriptomic level which were associated with subjects showing either high or low antibody levels after two doses of inactivated SARS-CoV-2 vaccines. Moreover, we applied proteomics to examine the mechanisms that would influence antibody levels in sera from COVID-19 patients.

## RESULTS

### Serum samples and the characteristics of the subjects.

A total of 534 subjects who had received two doses of inactivated COVID-19 vaccines were recruited and consented to the collection of blood samples. Samples taken less than 14 days after the second vaccine or from convalescent COVID-19 patients were removed from the subsequent analysis, leaving a total of 526 samples (Table 1, Table S1 in the supplemental material). Based on each individual’s willingness, at least some of the following clinical information was collected: gender, the brand names of the vaccines received, days since the second vaccination, age, family history of cardiovascular diseases, and BMI. Of the subjects, 301 (57.2%) were female and 225 (42.8%) were male (Table 1). Of the subjects, 286 (54.37%) received Sinovac CoronoVac and 136 (25.86%) received Sinopharm BBIBP-CorV, as determined by the availability of these vaccines (Table 1). The cohort age ranged from 18 to 75 years old with an average age of 30.91 (standard deviation [SD]: ±13.58) (Table 1, Fig. S1A). A total of 44 (8.365%) subjects had a family history of cardiovascular diseases (Table 1). According to BMI classifications of overweight and obese, 295 (56.1%) subjects had a healthy weight (BMI), 49 (9.3%) were underweight, 126 (24.0%) were overweight, and 19 (3.6%) were obese (Table 1).

**TABLE 1:** Study subject characteristics^*a*^

Characteristic	*N* (%)^*b*^
Gender	
Male	225 (42.8)
Female	301 (57.2)
Vaccine brand	
Sinovac	286 (54.37)
Sinpharm	136 (25.86)
NA	104 (19.77)
Interval length, days ± SD (range)	61.44 ± 30.66 (16–282)
15–30 days	37 (7.034)
30–60 days	253 (48.099)
60–90 days	126 (23.954)
90–120 days	32 (6.084)
≥120 days	33 (6.274)
NA	45 (8.555)
Age (yrs)	
Mean ± SD	30.91 ± 13.58
Range	18–75
Family history of hypertension or cardiovascular disease	
Yes	44 (8.365)
No	440 (83.650)
NA	42 (7.985)
BMI	
<18.5	49 (9.3)
18.5–24	295 (56.1)
24−30	126 (24.0)
≥30	19 (3.6)
NA	37 (7.0)

a NA, not applicable; SD, standard deviation; BMI, body mass index.

b Values are given as *n* (%) unless otherwise specified.

None of the enrolled individuals reported exposure to known COVID-19 patients, had a documented history of infection with SARS-CoV-2, or exhibited symptoms of a new or worsening cough, sneezing and runny nose, fever, temporary loss of smell or altered sense of taste, sore throat, or shortness of breath over a minimum of 8 weeks. Each subject had an interval of at least 14 days between administration of the second dose and the day of serum sample collection, as no antibodies were expected to be induced in less than this time frame. A total of 253 (48.099%) subjects had a 30- to 60-day interval after the second dose of vaccination, 37 (7.034%) had a 15- to 30-day interval, 126 (23.954%) had a 60- to 90-day interval, 32 (6.084%) had a 90- to 120-day interval, and 33 (6.274%) had more than a 120-day interval (Table 1, Fig. S1B). Notably, none of the subjects exhibited adverse reactions such as fatigue, dizziness, or headache.

### The duration of anti-Spike RBD antibodies in serum less than 150 days after vaccination.

**FIG 1:**
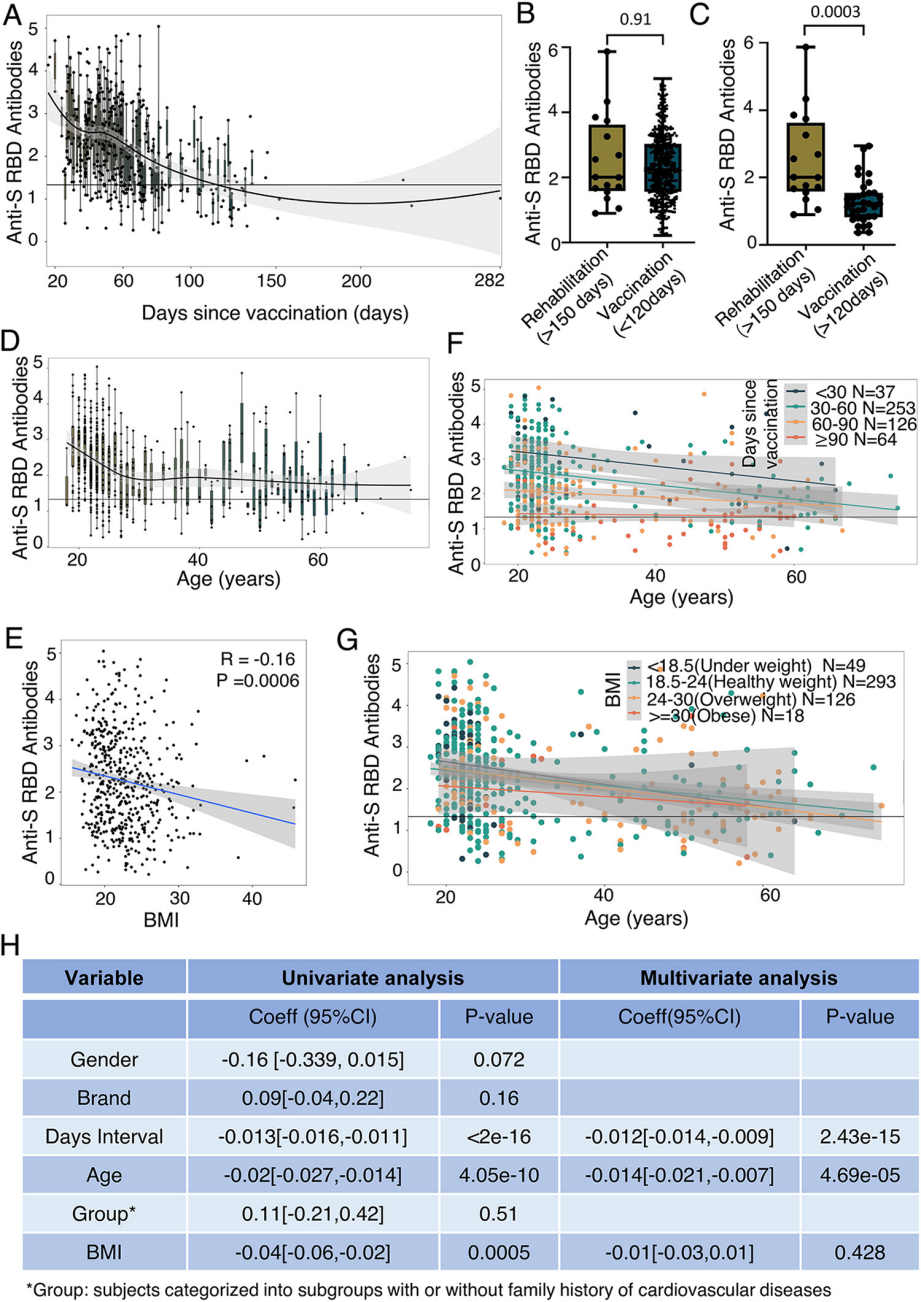
Waning anti-Spike receptor-binding domain (RBD) antibody levels associated with increasing age or body mass index (BMI). (A) Distribution of anti-Spike RBD antibody levels over time intervals between antibody testing and vaccination. (B) Antibody levels in sera from convalescent COVID-19 patients and vaccinated subjects with a time interval of <120 days. (C) Antibody levels in serum of convalescent COVID-19 patients and vaccinated subjects with a time interval of >120 days. (D) Antibody level distribution by age. (F) Antibody level distribution by age for vaccinated subjects with time intervals of  <30, 30 to 60, 60 to 90, and ≥90 days. (E) Correlation between antibody levels and BMI. (G) Antibody level distribution over age for underweight, healthy weight, overweight, and obese vaccinated subjects. (H) Uni- and multivariate linear regression analysis of gender, vaccine brand, days of interval, age, group, and BMI with antibody levels. “Brand” indicates Sinovac CoronoVac or Sinopharm BBIBP-CorV vaccine. “Group” means subjects with or without a family history of cardiovascular disease. *N* = number of subjects.

Because the RBD within the S1 subunit of the Spike (S) protein interacts directly with receptors of the host cells, antibodies directed against the receptor-binding domain (RBD) domain can block pathogen entry into target cells (11). Evaluation of anti-Spike RBD antibodies is useful for estimating an individual’s protection against SARS-CoV-2 infection (11). The levels of anti-Spike RBD antibodies were determined for all 526 subjects who received two doses of inactivated COVID-19 vaccines. Positive anti-Spike RBD antibodies were observed in 77.38% (407 of 526) subjects. We discovered that the longer the interval since the second dose of vaccine, the lower the levels of anti-Spike RBD antibodies observed (Fig. 1A). Positive anti-Spike RBD antibodies were detected significantly more frequently in subjects within 120 days after administration of the second dose of inactivated SARS-CoV-2 vaccine (positivity: 363 of 448 subjects with an interval of <120 days, 13 of 28 subjects with an interval of ≥120 to <150 days, 1 of 5 subjects with an interval of ≥150 days), suggesting that the majority of subjects would have no detectable anti-Spike RBD antibodies in sera by 150 days after the second vaccination.

Additionally, we determined the levels of anti-Spike RBD antibodies in sera from 16 convalescent COVID-19 patients at 150 days after recovery collected from Beijing You An Hospital and found that their anti-Spike RBD antibody levels were similar to those detected in the sera of vaccinated subjects collected within 120 days of vaccination (Fig. 1B), but statistically significantly higher than those collected more than 120 days post-vaccination (Fig. 1C). These results demonstrated that the duration of anti-Spike RBD antibodies was longer in the sera of convalescent COVID-19 patients than in sera of vaccinated subjects who had received two doses of inactivated SARS-CoV-2 vaccine.

### Decreased anti-Spike RBD antibodies associated with increased age or BMI.

To investigate the influence of different factors on anti-Spike RBD antibody levels after administration of inactivated SARS-CoV-2 vaccine, we examined the effects of age, gender, the brand of inactivated SARS-CoV-2 vaccine received (Sinovac CoronoVac or Sinopharm BBIBP-CorV), duration of post-vaccination interval, family history of cardiovascular diseases, and BMI. We discovered that antibody levels varied with age, with subjects 30 years old or younger having higher antibody levels than older subjects. The older the subject, the lower the level of antibodies detected (Fig. 1D). We also observed a weak negative correlation between anti-Spike RBD antibody levels and BMI (R = −0.16, *P = *0.0006) (Fig. 1E). To exclude possible influence of the number of days post-vaccination, we divided all subjects into four categories: <30, 30 to 60, 60 to 90, and ≥90 days post-vaccination. Irrespective of interval duration, anti-Spike RBD levels tended to wane with increasing age in all four subject categories (Fig. 1F). Similarly, all subjects were categorized into underweight (*n* = 49, BMI < 18.5), healthy weight (*n* = 295, 18.5 ≤ BMI < 24), overweight (*n* = 126, 24 ≤ BMI < 30), and obese (*n* = 19, BMI ≥ 30) subgroups. Anti-Spike RBD antibody levels were significantly lower in obese subjects than in other subgroups (*P < *0.05) (Fig. S1C). Regardless of BMI, the levels of anti-Spike RBD antibodies decreased with increasing age (Fig. 1G). A trend of females having higher anti-Spike RBD antibody levels than males was observed but did not reach statistical significance (*P = *0.054; Fig. S1D). The levels of anti-Spike RBD antibodies were higher in subjects who received two doses of Sinovac CoronoVac than in those who received Sinopharm BBIBP-CorV inactivated SARS-CoV-2 vaccines, but this difference was not statistically significant (*P = *0.055; Fig. S1E).

Single- and multivariate linear regression analysis were further performed to evaluate the associations between anti-Spike RBD antibody levels and different factors (gender, vaccine brand, duration of post-vaccination interval, age, family history of cardiovascular diseases, and BMI) (Fig. 1H). We discovered that the number of days since vaccination (*P < *2e–16), age (*P = *4.05e–10) and BMI (*P = *0.0005) were significantly associated with anti-Spike RBD antibody levels as demonstrated by single-variate linear regression analysis. Moreover, multivariate linear regress analysis revealed that the number of days since vaccination (*P = *2.43e–15) and age (*P = *4.69e–05) were independent factors.

Angiotensin-converting enzyme (ACE) polymorphism has been associated with various heart-related and other diseases such as atherosclerosis (12), myocardial infarction (13), ischemic stroke (14), and hypertension (15). ACE deletion polymorphism was recently reported to be associated with susceptibility to COVID-19 in a risk-dependent manner among the Chinese population (16). We therefore examined the levels of anti-Spike RBD antibodies in subjects with a family history of cardiovascular disease and found no difference with those in subjects with no family history of cardiovascular disease (*P = *0.482; Fig. S1F). We determined the ACE genotype, including D/D and I (I/I or D/I), for each subject, and discovered that anti-Spike RBD antibody levels were not statistically significantly different between subjects with the ACE D/D genotype and those with the I genotype (*P* = 0.6853; Fig. S1G). For subjects with a family history of cardiovascular disease, we found no significant differences in antibody levels between subjects with ACE D/D and those with I genotypes (*P = *0.95; Fig. S1H). Similarly, for those with no family history of cardiovascular disease, we observed no significant differences in antibody levels between subjects with ACE D/D and those with I genotypes (*P = *0.94; Fig. S1H).

### Non-responders to vaccination with impaired B-cell activating humoral functions.

**FIG 2:**
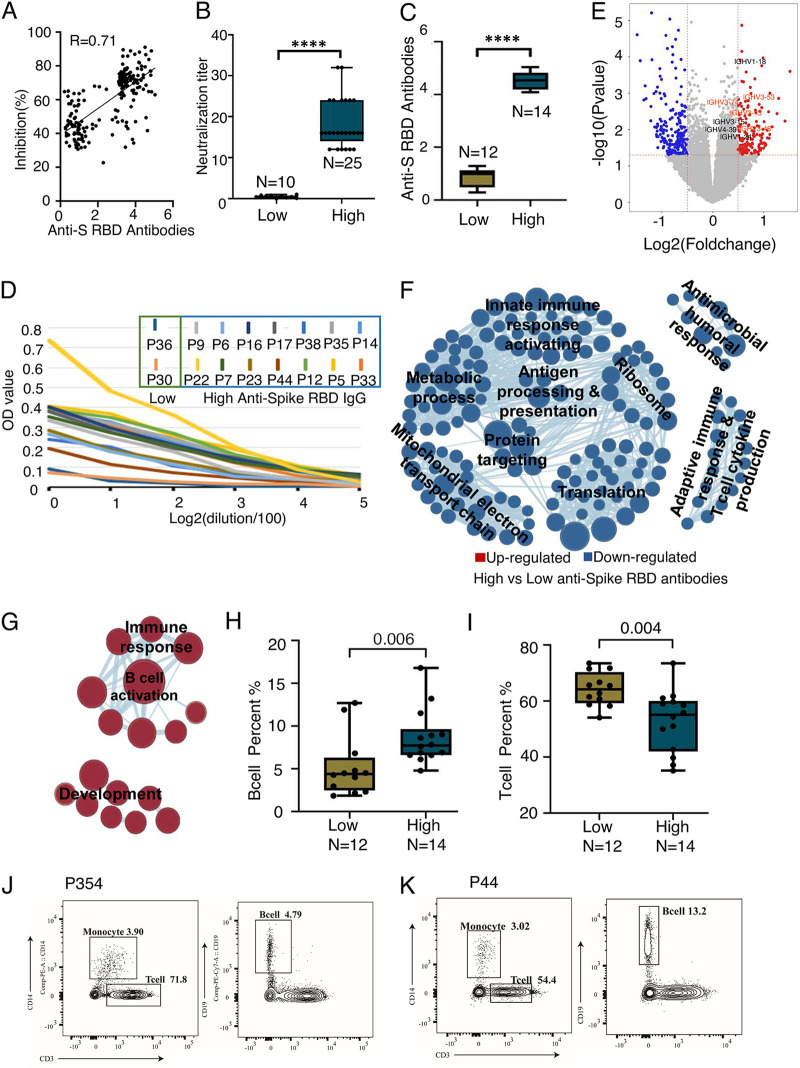
Immune responses to vaccinations at transcriptomic level. (A) Correlation between anti-Spike RBD antibody levels and inhibition (%). (B) Neutralization titer between samples with highest antibody levels and those without antibodies detected. (C) Comparison of anti-Spike RBD antibody levels between 14 vaccinated subjects with the highest antibody levels and 12 randomly selected vaccinated subjects with no antibodies detected. *P* was calculated using Mann-Whitney-U test (****, *P* < 0.0001). *N* = number of subjects. (D) Anti-Spike RBD antibody levels in a serial dilution of sera from 14 vaccinated subjects with highest antibody levels compared with those from two vaccinated subjects with no antibodies detected as a negative control. (E) Significantly differentially expressed genes between blood of vaccinated subjects with high antibody levels and that of subjects with no antibodies detected. Red and blue dots represent up- and downregulated genes in vaccinated subjects with high antibodies. (F and G) Enrichment map network of statistically significant Gene Ontology (GO) categories in vaccinated subjects with high antibodies (G) and with no antibodies (F). (H and I) Boxplot analysis showing the frequency of T cells (I) and B cells (H) of CD45^+^ cells between 14 vaccinated subjects with antibodies and 12 vaccinated subjects without antibodies. *P* was calculated by Mann-Whitney U test. (J and K) Flow cytometric analysis showing the frequency of T cells, B cells, and monocytes for vaccinated subjects (P354) without anti-Spike RBD antibodies (J) and vaccinated subjects (P44) without anti-Spike RBD antibodies (K).

To further examine whether samples with anti-Spike RBD antibodies have neutralization effects, we first used competitive enzyme-linked immunosorbent assay (ELISA) upon sera from 201 randomly selected vaccinated subjects to detect the antibodies that compete with ACE2 to bind to RBD. Sera from vaccinated subjects with stronger anti-Spike RBD antibodies were also found to contain anti-RBD antibodies with stronger ability to bind to RBD when competing with ACE2 (R = 0.71, Fig. 2A, Table S1). Additionally, the neutralizing activities of the sera were tested with SARS-CoV-2 wild-type virus in 35 samples, including samples from 25 subjects with the highest levels of anti-Spike RBD antibodies and 10 subjects which did not respond to vaccination. We observed that samples with the highest levels of anti-Spike RBD antibodies had significantly higher neutralizing activities than those from non-responders (Fig. 2B, Table S2). These results indicated that samples with anti-Spike RBD antibodies, but not samples from non-responders, had neutralization effects.

To determine genome-wide transcriptional changes in non-responders (22.62%, 119 of 526) due to vaccination by inactivated SARS-CoV-2 vaccines, we performed high-throughput RNA sequencing (RNA-seq) on peripheral blood mononuclear cells (PBMCs) from each of the 26 subjects, including 14 subjects with the highest levels of anti-Spike RBD antibodies at a more than 40-day interval post-vaccination and randomly selected 12 subjects who did not respond to vaccination (Fig. 2C, Table S3). The anti-Spike RBD antibody levels in sera from 14 subjects with the highest antibody levels were further confirmed by a serial dilution of serum (Fig. 2D). Both competitive inhibition ELISA and a neutralization assay independently confirmed that samples with the highest anti-Spike RBD antibody levels had stronger neutralization effects than those from non-responders (Table S2 to 3). A total of 159 up- and 217 downregulated genes were identified in sera from subjects with high antibody levels compared with those from non-responders (Fig. 2E, Table S4 to 5). We discovered that the expression levels of immunoglobulin heavy variable (IGHV) genes were upregulated in vaccinated healthy subjects with anti-Spike RBD antibodies, suggesting that IGHV genes play important roles in recognizing foreign antigens and initiating immune responses such as phagocytosis and the complement system. Gene set enrichment analysis demonstrated that B-cell activation and a few immune response categories were highly enriched in the sera from subjects with high anti-Spike RBD antibody levels (Fig. 2G, Table S6), consistent with the knowledge that exposure to an antigen by vaccination leads to the activation of B lymphocytes (17). Notably, several immune response categories, such as antigen processing and presentation, innate immune response activation, adaptive immune response, and T cell cytokine production, were highly enriched in the sera of non-responders to vaccination (Fig. 2F, Table S7), thus providing evidence for impaired B-cell activation of humoral functions in subjects who did not respond to vaccines. We also performed flow cytometry on PBMCs from the 26 subjects to analyze the proportion of T cells, B cells, and monocytes of CD45^+^ cells (Fig. S2A, Table S8), discovering that the proportions of T cells were significantly higher in the 12 vaccinated subjects without anti-Spike RBD antibodies than in the 14 vaccinated ones with anti-Spike RBD antibodies (*P* = 0.004, Fig. 2I, J, and K, Fig. S2B to C). In contrast, the proportions of B cells were significantly higher in the 14 vaccinated subjects with anti-Spike RBD antibodies than in the 12 vaccinated ones without anti-Spike RBD antibodies (*P* = 0.006, Fig. 2H, J, and K, Fig. S2B to C), while the proportions of monocyte cells were not significant (Fig. S2D, *P* = 0.18).

**FIG 3:**
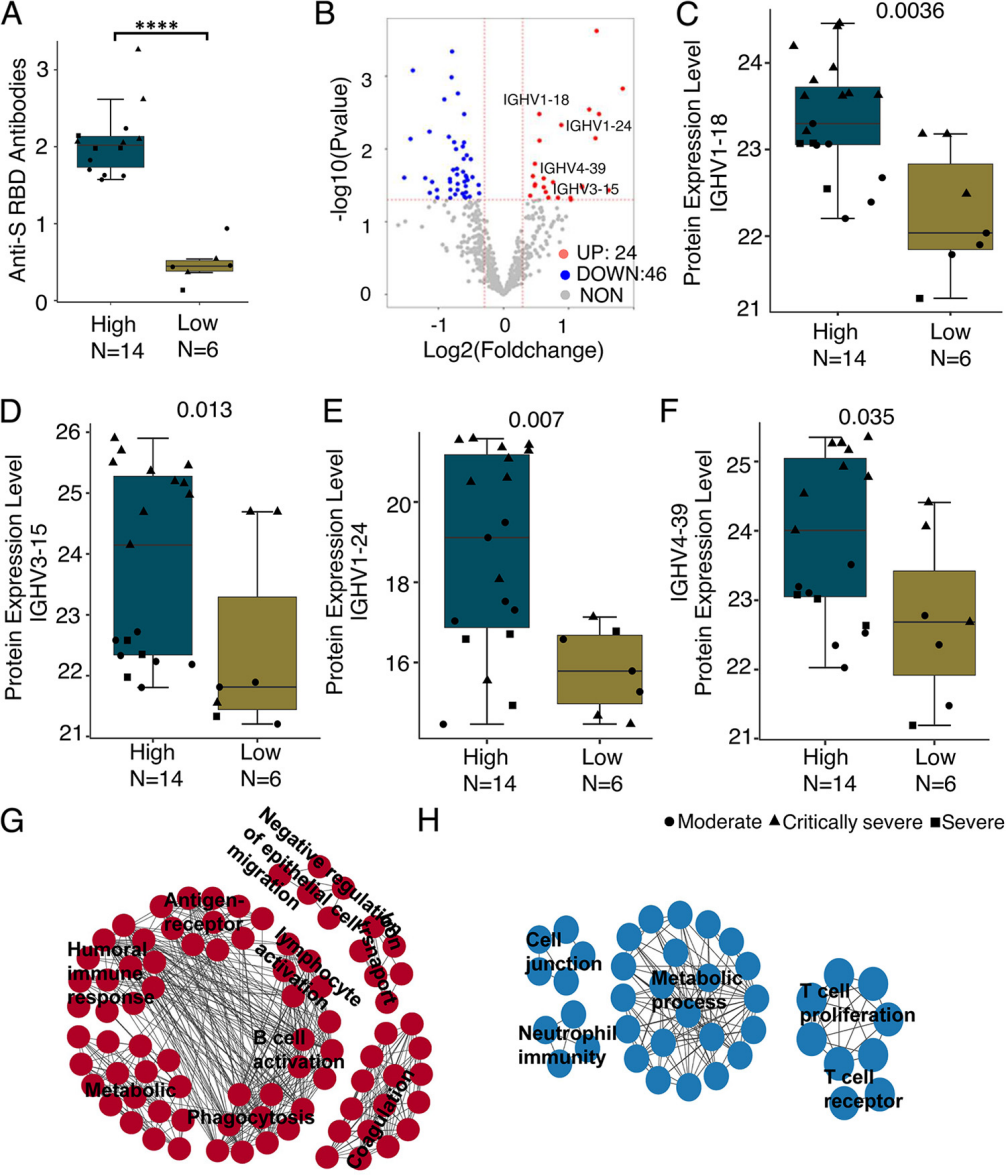
Immune responses to SARS-CoV-2 infection at proteomics level. (A) Levels of anti-Spike RBD antibodies between patients with and without detectable antibodies. *N* = number of patients. *P* was calculated by Mann-Whitney U test (****, *P* < 0.0001). (B) Significantly differentially expressed proteins in blood from COVID-19 patients with and without anti-Spike RBD antibodies detected. In particular, (C) IGHV1-18, (D) IGHV3-15, (E) IGHV1-24, and (F) IGHV4-39. IGHV, immunoglobulin heavy variable. The gene represents the corresponding protein group. (G and H) Enrichment map network of statistically significant GO categories in COVID-19 patients with (G) and without antibodies (H). Red and blue dots represent up- and downregulated genes in vaccinated subjects with high antibody levels. Dot shapes in the boxplot indicate clinical classifications, with circles representing moderate disease, triangles representing critically severe disease, and squares representing severe disease.

We therefore hypothesize that an encounter with antigens quickly induces antibodies in subjects with good B-cell activating humoral immunity. We collected plasma from 20 patients (9 moderate, 4 severe, 7 critically severe) at 4 to 18 days after a diagnosis of COVID-19 in January 2020 at the Hubei Center for Disease Control and Prevention in Wuhan, Hubei, China (Table S9). The levels of anti-Spike RBD antibodies were determined for each subject, revealing positive antibody detection for 14 subjects (6 moderate, 3 severe, 5 critically severe) but not for 6 subjects (3 moderate, 1 severe, 2 critically severe) (Fig. 3A). We then performed a DIA mass spectrometry experiment and obtained 697 protein groups. Differential protein expression analysis identified 24 up- and 46 downregulated proteins in patients with antibodies compared to those without antibodies (Tables S10 to S11). In particular, IGHV genes were highly expressed in patients with anti-Spike RBD antibodies (Fig. 3B to F, Tables S10 to S11), suggesting that the B cell-associated immune response had been activated. Gene Ontology (GO) enrichment analysis confirmed that the B cell activation and humoral immune response categories were statistically significantly enriched in patients with antibodies detected in their plasma (Fig. 3G, Table S12). In contrast, there was statistically significant enrichment of metabolic process and neutrophil-mediated immunity, and a non-negligible trend (not significant) for the enrichment of T cell-associated immune response categories in patients who did not yet have antibodies generated (Fig. 3H, Table S13). Together, these results demonstrated that B cell activation of humoral immunity was impaired in subjects who failed to generate anti-Spike RBD antibodies after receiving inactivated SARS-CoV-2 vaccines.

## DISCUSSION

Vaccines have been developed at an unprecedentedly fast speed to contain the SARS-CoV-2 infection among the population. A large portion of the world population has been administered the inactivated vaccines Sinovac CoronoVac and Sinopharm BBIBP-CorV (4, 8–10). It is critical to assess vaccine efficacy by measuring the levels of antibodies directed against the RBD, which are most relevant to their neutralizing activity (11, 18). Accordingly, it is essential to understand the possible molecular mechanisms for why some subjects fail to produce antibodies against SARS-CoV-2 Spike protein after vaccination. Therefore, the surveillance of circulating anti-Spike RBD antibody levels would be informative on acquired immunity against SARS-CoV-2.

To the best of our knowledge, our study was performed on one of the largest samples of the Chinese population to date. In the current study, we sought to evaluate the duration of anti-Spike RBD antibodies, the factors which influence antibody levels, and the molecular mechanisms leading to the failure of antibody production post-vaccination with inactivated SARS-CoV-2 vaccines. We first investigated the anti-Spike RBD antibody levels of a large cohort, including 526 subjects who had received two doses of inactivated COVID-19 vaccines. We found that 77.38% (407 of 526) subjects successfully produced anti-Spike RBD antibodies, which were detectable in 81.03% (363 of 448) of subjects within 120 days post-vaccination, 46.43% (13 of 28) of subjects 120 to 150 days post-vaccination, and 20% (1 of 5) of subjects at more than 150 days post-vaccination. These data suggested that the antibodies generated by vaccination rarely last longer than 150 days. In contrast, the antibodies in convalescent COVID-19 patients at 150 days after recovery were still maintained at similar levels to those detected in subjects within 120 days of vaccination.

Of the multiple factors which may influence antibody levels, our results demonstrated that elderly subjects had significantly lower levels of antibodies than younger subjects. Similar results have also been reported for the mRNA BNT162b2 COVID-19 vaccine (19). Decreased antibody levels were also observed to be associated with increasing BMI. Balanced diet and exercises are important to maintain BMI within a healthy range. Females displayed a trend of higher antibody levels than males (no statistical significance), while statistically significantly higher antibody levels were reported in females than in males for the mRNA BNT162b2 COVID-19 vaccine (19). Higher antibody levels were observed among subjects who received Sinovac CoronoVac than in those who received Sinopharm BBIBP-CorV, but there was no statistical significance. Neither ACE polymorphism genotype nor a family history of cardiovascular disease were found to influence antibody levels. After comparing our findings with previous reports on the antibody levels induced by mRNA vaccines (20, 21), we found that antibody levels decreased gradually, which was similar for both mRNA and inactivated vaccines.

For the subjects who failed to produce anti-Spike RBD antibodies after vaccination, we found enrichment of T cell-associated immune response categories and a lack of B-cell activation and humoral immunity. We therefore speculate that T cell-associated immune response categories may be universally enriched in antigen-stimulated subjects unable to produce anti-Spike RBD antibodies. Compared with non-infected, non-vaccinated subjects (PBMC RNA-seq data from the public domain), we observed that two more T cell-activation-associated categories (positive regulation of CD4^+^ alpha-beta T cell activation; alpha-beta T cell activation) were significantly enriched in vaccinated subjects without anti-Spike RBD antibodies compared to vaccinated subjects with anti-Spike RBD antibodies (*P* < 0.05, normalized enrichment score (NES) value > 1) (Table S14). In fact, in COVID-19 patients without anti-Spike RBD antibodies detected 4 to 18 days after diagnosis, we also found significant enriched neutrophil immunity and metabolic process, and a non-negligible trend (not significant) for the enrichment of T cell-associated immune response categories. More investigations are needed to examine the T cell-associated immune response and its relationship with impaired B-cell activation of humoral immunity. Additionally, the enrichment of neutrophil immunity suggested that neutrophil-mediated immunity plays an earlier role in battling SARS-CoV-2 than anti-Spike RBD antibody, and the exact relationship between neutrophil-mediated immunity and antibody production will be investigated in the future.

The present study has some limitations. About 60% of the study subjects were less than 30 years old. More subjects of different ages are needed in the future. Because there is a strong correlation between the levels of neutralizing antibodies and anti-S1/S2 antibodies for the mRNA vaccine (22), we measured anti-Spike RBD antibody levels in subjects vaccinated with inactivated SARS-CoV-2 vaccines. We also applied both competitive inhibition ELISA and a neutralization assay to independently verify that samples with high anti-Spike RBD antibody levels had stronger neutralization effects than samples from non-responders. These neutralizing activities also need to be studied in a larger vaccinated population in the future. The portion of participants with a family history of cardiovascular disease was considerably smaller, which may have led to underestimation of its impact on antibody levels. Additionally, the proportion of subjects who received the Sinopharm BBIBP-CorV vaccine was relatively smaller, probably leading to underestimation of the differences between the two vaccines.

In conclusion, this study showed the duration of anti-Spike RBD antibodies produced by two doses of inactivated SARS-CoV-2 vaccines, the factors which influence antibody levels, and impaired humoral immunity observed in the subjects unable to produce antibodies after vaccination. Our findings underscore the need for a booster dose within 150 days of vaccination with two doses of inactivated SARS-CoV-2 vaccines.

## MATERIALS AND METHODS

### Subjects.

We recruited healthy human subjects from Beihang University and Beijing You An Hospital through a campus-wide email, inviting them to provide serum samples and throat swabs from May to August 2021. A total of 534 people responded, completing a medical-history questionnaire before providing samples for study. No inclusion criteria were used other than a requirement that subjects without SARS-CoV-2 infection have received two doses of inactivated SARS-CoV-2 vaccine produced by either Sinovac CoronoVac or Sinopharm BBIBP-CorV. Sixteen convalescent COVID-19 patients at 150 days after recovery from Beijing You An Hospital and 20 COVID-19 adult Chinese patients from Hubei Province were recruited from January to March 2021. All patients were diagnosed with COVID-19 by a combination of PCR, chest computerized tomography (CT) scan, and antibody tests (23) (National Health Commission, 2020, Protocol on Prevention and Control of COVID-19, 6th edition) and clinical classification was conducted according to “Chinese Clinical Guidance for COVID-9 Pneumonia Diagnosis and Treatment (7th edition),” including “Mild,” “Moderate,” “Severe,” and “Critically Severe.” All experimental procedures, including human sampling, were approved by the Hubei Center for Disease Control and Prevention (ethical approval no. 2021-012-01) and the Beijing You An Hospital research ethics committee (approval no. LL-2020-035-K).

### Biosafety.

All blood samples were treated strictly according to biocontainment procedures for the processing of SARS-CoV-2–positive samples.

### Anti-SARS-COV-2 Spike RBD antibody levels in serum.

Anti-Spike RBD antibody levels were measured with a kit (KIT002, Sino Biological, China) according to the manufacturer’s instructions (sera from 534 healthy vaccinated subjects processed at Beihang University, 16 convalescent patients at Beijing You An Hospital, and 20 COVID-19 patients processed at the Xiantao Center for Disease Control and Prevention). The principal component of the KIT002 kit is an indirect ELISA. SARS-CoV-2 spike RBD-His recombinant protein was pre-coated onto well-plate strips. The sample or control antibodies were added to the well; after incubation, the wells were washed, and a horseradish peroxidase-conjugated goat anti-human IgG was added. After a wash, the TMB (3, 3′, 5, 5′-tetramethylbenzidine) substrate solution was loaded, and colors developed in proportion to the amount of antibodies. The reaction was stopped by the addition of a stop solution, and color intensity was measured at 450 nm.

### Competitive inhibition ELISA.

A SARS-CoV-2 inhibitor screening kit (KIT001, Sino Biological) was used to screen for SARS-CoV-2 inhibitors, and the neutralizing effect of the sample was tested through the competitive ability of ACE2 to bind to RBD in serum. In this kit, SARS-CoV-2 Spike RBD-mFc recombinant protein is already pre-coated onto 96-well plate strips. After washing, 100 μL human ACE2-His-tag and 100 μL plasma with phosphate-buffered saline PBS isobaric dilution was added and the sample was incubated for 1 h at room temperature. Next, 100 μL of anti-His-Tag-HRP was added after washing and the sample was incubated for 1 h at room temperature. Finally, 200 μL substrate solution was added and the sample was incubated for 15 min at room temperature. The reaction was stopped by the addition of a stop solution, and color intensity was measured at 450 nm (OD_450_, optical density at 450 nm). The signal color becomes lighter as the SARS-CoV-2 inhibitor content increases. The positive and negative critical values of the kit can be used to judge whether the sample has a neutralization effect:

$$\text{Inhibition}=1-\;\frac{{\text{OD}}_{\text{450}}\;\text{of sample}}{{\text{OD}}_{\text{450}}\;\text{of negative control}}\;\times 100\%$$

### Authentic SARS-CoV-2 neutralization assay.

The SARS-CoV-2 wild-type strain was used. All SARS-CoV-2 authentic virus-related experiments were approved by the biosafety level 3 committee (ABSL-3) of Wuhan University, Wuhan Institute of Virology and Hubei Provincial Center for Disease Control and Prevention. Virus was diluted to 100 CCID_50_ (50% cell culture infective dose)/0.05 mL with Dulbecco’s modified Eagle medium (DMEM) maintenance solution (10% fetal bovine serum [FBS] + 1% Strep) and sample sera were made at a 2-fold ratio dilution with DMEM maintenance solution, starting at 1:4. Next, 140 μL different dilution of serum and 70 μL virus solution (100 CCID_50_/0.05 mL) was added to a 96-well plate and incubated for 2 h at 37°C. Viral control and normal cell control were established. After incubation, the serum-virus mixture was inoculated onto cultured monolayer Vero cells, 2 wells per dilution with 100 CCID_50_ of virus in each well, and incubated at 37°C in a 5% CO_2_ incubator. After neutralization, virus suspensions of 100 CCID_50_/50 μL, 10 CCID_50_/50 μL, 1 CCID_50_/50 μL, and 0.1 CCID_50_/50 μL were obtained by diluting the virus with virus diluent (DMEM + 2% neonatal bovine serum + 1% double antibody). Four dilutions of attack virus were added in 96-well plates, 8 wells/dilution, 50 μL per well, supplemented with 50 μL virus dilution, and cell control wells (8 wells) were set up. After 3 to 5 days of incubation, the cytopathic effects (CPE) in each serum dilution were observed and recorded under an inverted microscope. When CPE changes appeared in the experimental group cells, the group was marked as “+”; otherwise, it was marked as “–.” Finally, neutralization titers were determined.

### ACE genotypes.

A Tianlong DNA extraction kit (ZTLYB-Y64; Wuhan, China) was applied to extract DNA from throat swab samples from the 534 healthy vaccinated subjects enrolled, which were then stored at –80°C refrigerators at Beihang University. The relevant fragment in intron 16 of the ACE was amplified using previously reported methods (24). Briefly, DNA was amplified in a 20-μL mixture of primers (5 pmol/primer; primer 1m 5′-TGG AGA GCC ACT CCC ATC CTT TCT-3′; primer 2m 5′-GAC GTG GCC ATC ACA TTC GTC AGA T-3′; Tianyihuiyuan, Wuhan, China), 0.5 U of *Taq* polymerase (TaKaRa Bio, Kusatsu, Japan, AJE0486A), and 2 μL of two *PCR buffers (TaKaRa Bio) with 2.5 mM MgCl_2_ and 0.2 mM dNTP. PCR (Mastercycler X50s; Eppendorf, Hamburg, Germany) was then performed as described below: 5-min initial denaturation at 95°C; 30 cycles of 30 s at 94°C (denaturation), 30 s at 54°C (annealing), and 1 min at 72°C (extension); and a 5 min elongation at 72°C. The D and I alleles resulted in 192-bp and 479-bp PCR products, respectively. Due to the preferential amplification of the D allele in heterozygous samples, all samples with the DD genotype were used in a second insertion-specific PCR (25) (primer 1, 5′-TGG GAC AGC GCC CGC CAC TAC-3′; primer 2, 5′-TCG CCA GCC CTC CCA TGC CCA TAA-3′; Tianyihuiyuan, Wuhan, China). The PCR was run as follows: 5-min initial denaturation at 95°C; 30 cycles of 30 s at 94°C, 45 s at 62°C, and 40 s at 72°C; and 5-min elongation at 72°C. The I allele resulted in a 335-bp PCR product, and no products were detected for homozygous DD samples.

### RNA-seq data analysis.

RNA-seq was performed upon PBMCs of 26 healthy vaccinated subjects (14 subjects with the highest anti-Spike RBD antibody levels and 12 randomly selected subjects without antibodies) by Novogene Co., Ltd. Genomic RNA (Life Technologies, Rockville, MD, cat no. 15596-018) was extracted and the mRNA-Seq Sample Prep kit (Illumina, San Diego, CA) was used to construct RNA-seq libraries according to the manufacturer’s instructions. The 7G raw FASTQ data per sample were generated by the Illumina HiSeq 2000 platform. FASTQ data were aligned to the human reference genome (hg19) using STAR (26) at default settings and HTSeq-count (27) was used to compute read counts for each gene. DEseq2 (28) was applied to identify differential expression genes with a *P* value cutoff of 0.05 and fold-change value cutoff of 2. Gene Set Enrichment Analysis (GSEA) (29) was performed upon normalized gene expression profiles output from DEseq2 to derive enriched GO terms at default settings (FDR cutoff of 25%, permutation type of gene set). Cytoscape (v3.9.0) (30) was used to generate a GO enrichment network.

We also collected RNA-seq data of PBMCs from a total of 22 non-infected, non-vaccinated healthy samples from previous studies, including raw RNA sequencing data from 5 healthy PBMC human donors (GSE162562 [31]) and PBMC gene expression read count data from 17 healthy donors (GSE152418 [32]). Gene expression read data were analyzed using STAR and HTSeq-count as described above. We used the ComBat function in the sva package (3.38.0) (R version 4.0.2) to remove the batch effect, then performed GSEA on data from non-infected, non-vaccinated healthy subjects, vaccinated subjects without anti-Spike RBD antibodies, and vaccinated subjects with anti-Spike RBD antibodies.

### Mass spectrometry data analysis.

Plasma samples from 20 COVID-19 patients were collected at the Xiantao Center for Disease Control and Prevention. Data-independent acquisition (DIA) mass spectrometry was used for plasma proteomic analysis. Each peptide sample was analyzed by liquid chromatography-tandem mass spectrometry (LC-MS/MS) operating in DIA mode by Shanghai Applied Protein Technology Co., Ltd. Each DIA cycle contained one full mass spectrometry-selected ion monitoring (MS-SIM) scan, and 30 DIA scans covered a mass range of 350 to 1,800 *m/z* with the following settings: SIM full scan resolution, 120,000 at 200 *m/z*; automatic gain control (AGC), 3e6; maximum IT, 50 ms; profile mode; DIA scans set at a resolution of 15,000; AGC target, 36; max IT auto; normalized collision energy, 30 eV. The runtime was 90 min with a linear gradient of buffer B (80% acetonitrile and 0.1% formic acid) at a flow rate of 250 nL/min. Pooled samples from equal aliquots of each sample in the experiment were used as quality control samples, which were injected in DIA mode at the beginning of the MS study and after every six injections throughout the experiment to monitor the MS performance.

DIA data were analyzed using Spectronaut Pulsar XTM searching the spectral library constructed in our previous study (16). The retention-time prediction type was set as dynamic iRT, and interference on MS2-level correction and cross-run normalization were enabled. All results were filtered using a *Q* value cutoff of 0.01.

The median normalization method was used for quantified proteins to reduce biases across samples. Limma was used to call differentially expressed proteins between patients with and without anti-Spike RBD antibodies (*P* < 0.05, fold-change value > 1.5). GO enrichment analysis was performed to obtain the enriched GO terms using clusterProfiler (v.3.16.1) (33) in R. Cytoscape (v.3.9.0) (30) was used to generate a GO enrichment network.

### Flow cytometric sorting experiment procedure.

First, PBMC cells were resuscitated from an –80°C refrigerator and heated in a 37°C water bath for 1 min until thawed. Next, we added 4 mL feeding solution (1640 + 20% FBS), centrifuged the cells at 500 × *g* for 10 min, and incubated them at 37°C for 30 min. Next, we added stain buffer (FBS) and centrifuged the cells at 1,800 rpm at 4°C for 5 min. The supernatant was discarded, and this process was repeated twice. The antibody was dissolved in FBS at a concentration of 1:200, including FITC (fluorescein isothiocyanate) mouse anti-human CD3, PE-CY7 mouse anti-human CD19, PE mouse anti-human CD14, and Alexa Fluor 700 mouse anti-human CD45 (Becton, Dickinson and Co., Franklin Lakes, NJ). Next, antibody was added to each sample and the samples were incubated at 4°C for 30 min. The centrifuge tube was shaken every 10 min during incubation to fully react the cells and antibody. Subsequently, FBS was used to centrifuge the mixture at 1,800 rpm at 4°C for 5 min. The supernatant was discarded, and this process was repeated twice. Finally, the cells were resuspended in 200 μL FBS, stored at 4°C, and sorted into types. An Aria II FACS (fluorescence-activated cell sorter) flow cytometer (Becton, Dickinson and Co.) was used to carry out the experiments. After being separated, cells were centrifuged at 1,800 rpm for 5 min at 4°C, after which the supernatant was discarded and the mixture was resuspended and held at 500 μL in an RNA Keeper Tissue Stabilizer. For subsequent analysis, the FCS data files were exported and imported into FlowJo v10 analysis software. For each sample, 20,000 cells were randomly selected on the machine, and 2 morphological channels, FSC (forward scatter) and SSC (side scatter), were used to exclude interference and identify lymphocyte subpopulations. Immune cells in PBMC were identified based on CD45 expression. Next, T cells were sorted as CD3^+^CD14^–^, monocyte cells were sorted as CD3^+^CD14^+^, and B cells were sorted as CD3^+^CD19^+^. The relative proportion of cells in each sample was obtained using FlowJo.

### Statistical analysis.

Statistical analyses were performed using Prism v8.0 (San Diego, CA, USA) and R. Data were presented as the mean and standard error of the mean. Differences in anti-Spike RBD antibody levels between groups were calculated using a Mann-Whitney-U test, *P* ≤ 0.05. Associations between anti-Spike RBD antibody levels and clinical factors were assessed using single- or multivariate linear regression analysis.

### Data availability.

The raw RNA-seq data of blood samples from 26 healthy vaccinated subjects have been deposited at the National Genomics Data Center (https://ngdc.cncb.ac.cn) under the accession number HRA001898. The original contributions presented in the study are publicly available in the ProteomeXchange Consortium (http://proteomecentral.proteomexchange.org) via the iProx partner repository with the data set identifier PXD028015.

### Ethics approval.

This study was conducted in accordance with the Declaration of Helsinki. The studies involving human participants were reviewed and approved by the Hubei Provincial Center for Disease Control and Prevention (ethical approval no. 2021-012-01) and the Beijing You An Hospital research ethics committee (approval no. LL-2020-035-K).
