# Supplementary material for: Impaired Humoral Immunity Identified in Inactivated SARS-CoV-2 Vaccine Recipients without Anti-Spike RBD Antibodies
Source: Microbiol Spectr. 2023 Mar 14;11(2):e02783-22. doi: 10.1128/spectrum.02783-22 (PMC10101072; doi:10.1128/spectrum.02783-22)
Supplement: Supplemental file 1 — Supplemental material. Download spectrum.02783-22-s0001.pdf, PDF file, 8.3 MB [file spectrum.02783-22-s0001.pdf]

Supplementary Materials

Supplementary Table 1. Clinical information of 534 subjects vaccinated by two doses of inactivated SARS-CoV-2 vaccines.

Supplementary Table 2. Clinical information of patients with neutralization assay.

Supplementary Table 3. Clinical information of 26 subjects with PBMC RNA-Seq data.

Supplementary Table 4. Up-regulated genes in serum of subjects with high vs no anti-Spike RBD antibodies.

Supplementary Table 5. Down-regulated genes in serum of subjects with high vs no anti-Spike RBD antibodies.

Supplementary Table 6. GO terms enriched in serum of subjects with high vs without anti-Spike RBD antibodies.

Supplementary Table 7. GO terms enriched in serum of subjects without vs with high anti-Spike RBD antibodies.

Supplementary Table 8. Percentage of T cells,B cells and Monocyte cells obtained by flow cytometric analysis.

Supplementary Table 9. Clinical information of 20 COVID-19 patients with DIA mass spectrometry experiment.

Supplementary Table 10. Up-regulated proteins in the plasma of COVID-19 patients with vs without anti-Spike RBD antibodies detected.

Supplementary Table 11. Down-regulated proteins in the plasma of COVID-19 patients with vs without anti-Spike RBD antibodies detected.

Supplementary Table 12. GO terms enriched in the plasma of COVID-19 patients with vs without anti-Spike RBD antibodies detected.

Supplementary Table 13. GO terms enriched in the plasma of COVID-19 patients without vs with anti-Spike RBD antibodies detected.

Supplementary Table 14. GO terms enriched in vaccinated individuals with/without antibodies vs Health group.

Supplementary Figure 1. The characteristics of vaccinated subjects.

Supplementary Figure 2. Flow cytometry analysis result for 26 subjects vaccinated.

Supplementary Table 1.Clinical information of 534 subjects vaccinated by two doses of inactivated SARS-CoV-2 vaccines.

Subject ID	Gender	Age	Height	Weight	BMI	Brand name of inactivated SARS-CoV-2 vaccine	Family history of cardiovascular diseases	Antibody level	Days after 2nd dose of inactivated	ACE Genotype	Inhibition (%)
P14	Male	23	181	65	19.841	Sinovac	NO	5.043	81	I	62.879
P33	Female	47	158	62	24.836	Sinovac	NO	4.864	63	NA	64.661
P423	Male	66	165	64	23.508	Rehabilitation	NO	4.845	NA	NA	88.880
P35	Female	21	166	53	19.234	Sinopharm	YES	4.82	56	NA	74.480
P12	Female	19	165	54	19.835	Sinovac	NO	4.82	70	I	76.879
P90	Female	21	158	53	21.231	Sinovac	NO	4.797	28	I	77.001
P114	Female	25	166	55	19.959	Sinopharm	NO	4.71	20	I	83.803
P44	Female	21	163	65	24.465	Sinovac	NO	4.707	59	I	64.831
P276	Female	NA	164	61	22.680	Sinovac	NA	4.642	NA	I	41.634
P380	Male	NA	NA	NA	NA	NA	NA	4.567	NA	NA	49.326
P6	Male	23	181	72	21.977	Sinovac	NO	4.564	56	I	68.560
P17	Male	21	180	70	21.605	Sinovac	NO	4.552	46	I	86.654
P16	Male	25	175	76	24.816	Sinovac	NO	4.513	51	I	79.359
P13	Male	19	172	65	21.971	Sinovac	YES	4.512	39	I	91.075
P133	Female	21	165	48	17.631	Sinovac	NO	4.423	34	I	66.195
P7	Female	23	168	80	28.345	Sinovac	NO	4.42	52	NA	74.635
P350	Male	26	186	76	21.968	Sinovac	NO	4.41	35	I	73.075
P85	Male	37	175	63	20.571	Sinovac	NO	4.323	17	DD	78.018
P93	Male	19	NA	NA	NA	NA	NA	4.32	29	I	79.886
P376	Female	56	162	60	22.862	Sinovac	NO	4.296	29	NA	71.517
P38	Female	60	164	65	24.167	Sinovac	NO	4.227	59	DD	67.602
P19	Male	19	170	62	21.453	Sinovac	NO	4.214	39	DD	88.152
P5	Female	19	163	49	18.443	Sinovac	NO	4.211	47	I	81.824
P230	Male	20	172	83	28.056	Sinovac	NO	4.181	40	DD	82.112
P86	Female	24	157	52	21.096	Sinopharm	NO	4.138	16	DD	74.274
P22	Female	20	162	55	20.957	NA	NO	4.114	70	I	69.729
P9	Male	21	164	51	18.962	NA	NO	4.111	70	DD	67.073
P91	Female	20	160	42	16.406	Sinovac	NO	4.109	27	I	78.480
P23	Female	22	165	67	24.610	Sinopharm	NO	4.081	49	I	71.525
P83	Female	24	163	70	26.346	Sinovac	NO	4.038	45	DD	63.656
P116	Female	23	164	55	20.449	Sinovac	NO	4.007	51	I	46.994
P289	Female	24	169	57	19.957	Sinovac	NO	3.964	31	I	86.855
P485	Male	NA	NA	NA	NA	NA	NA	3.956	NA	DD	70.420
P165	Male	20	175	78	25.469	Sinopharm	NO	3.941	56	I	72.172
P280	Female	51	155	53	22.060	Sinovac	NO	3.914	28	DD	63.873
P242	Female	21	163	65	24.465	Sinovac	NO	3.903	29	DD	34.381
P296	Female	25	160	55	21.484	NA	NO	3.902	35	I	69.784
P403	Female	NA	NA	NA	NA	Rehabilitation	NA	3.9	NA	DD	65.693
P74	Female	23	168	54	19.133	Sinovac	NO	3.896	46	DD	59.754
P82	Male	22	169.4	62	21.606	Sinopharm	YES	3.889	39	I	74.271
P250	Female	25	163	50	18.819	Sinopharm	NO	3.844	28	I	36.525
P72	Male	24	174	69	22.790	Sinovac	YES	3.835	46	I	72.466
P245	Female	23	175	54	17.633	Sinovac	NO	3.82	50	I	65.420
P516	Female	22	165	54	19.835	Sinovac	NO	3.772	NA	I	55.832
P272	Female	55	160	47.5	18.555	Sinovac	YES	3.765	NA	I	73.703
P258	Male	27	173	80	26.730	Sinovac	NO	3.744	53	DD	72.858
P203	Male	51	175	100	32.653	Sinovac	NO	3.742	60	I	88.871
P99	Male	20	NA	NA	NA	NA	NA	3.734	28	I	71.361
P273	Female	49	155	51	21.228	Sinovac	NO	3.734	NA	I	76.167
P84	Male	25	175	75	24.490	Sinovac	NO	3.685	46	I	73.528
P163	Male	21	181	53	16.178	NA	NO	3.654	56	I	75.406
P404	Female	21	167	64	22.948	Sinovac	NO	3.602	13	I	74.757
P77	Female	23	168	52	18.424	Sinovac	NO	3.595	47	DD	42.843
P154	Male	22	177	62	19.790	NA	NO	3.587	51	NA	85.833
P234	Female	21	160	61	23.828	Sinovac	YES	3.573	70	I	68.196
P215	Female	20	158	46	18.427	Sinovac	NO	3.56	40	DD	78.806
P158	Male	24	181	70	21.367	Sinovac	NO	3.559	53	DD	70.444
P251	Female	24	162	46	17.528	Sinopharm	NO	3.555	30	DD	72.401
P58	Male	21	170	58	20.069	Sinovac	NO	3.521	27	I	46.554
P124	Female	24	158	48	19.228	Sinovac	NO	3.516	20	I	77.919
P167	Male	22	175	70	22.857	Sinovac	NO	3.512	52	DD	67.937
P25	Male	21	182	67	20.227	Sinopharm	NO	3.508	48	DD	74.930
P364	Female	50	160	75	29.297	Sinovac	NO	3.503	41	DD	44.308
P259	Male	22	170	65.5	22.664	Sinovac	NO	3.495	59	I	71.319
P486	Female	38	160	65	25.391	NA	NA	3.489	30	I	68.672
P137	Female	23	159	60	23.733	Sinopharm	NO	3.488	46	I	70.965
P265	Female	22	157	48	19.473	Sinopharm	NO	3.483	30	NA	71.494
P155	Male	21	186	110	31.796	Sinovac	NO	3.461	69	I	70.068
P247	Male	23	160	63.5	24.805	Sinovac	NO	3.461	58	I	31.856
P238	Male	51	170	82	28.374	Sinovac	YES	3.449	69	DD	68.238
P169	Male	26	179	65	20.287	Sinovac	NO	3.449	60	DD	73.597
P10	Male	19	NA	NA	NA	NA	NA	3.435	8	I	42.443
P56	Female	24	163	52	19.572	Sinovac	NO	3.43	45	I	78.118
P24	Female	45	158	53	21.231	Sinovac	YES	3.393	57	DD	62.428
P71	Male	23	183	72	21.500	Sinovac	NO	3.385	45	I	76.638
P495	Female	NA	NA	NA	NA	NA	NA	3.382	NA	NA	72.655
P146	Male	19	183	78	23.291	Sinovac	NO	3.364	45	I	72.706
P281	Female	39	152	55	23.805	Sinovac	NO	3.362	24	I	62.636
P107	Female	21	158	48	19.228	Sinopharm	NO	3.359	55	I	71.062
P98	Female	28	158	50	20.029	Sinovac	NO	3.348	45	I	75.175
P70	Male	22	178	60	18.937	Sinopharm	NO	3.346	49	I	67.036
P191	Female	20	174	80	NA	Sinovac	NO	3.344	40	DD	89.517
P69	Female	22	168	52	18.424	Sinovac	NO	3.329	46	I	85.756
P175	Female	21	160	51	19.922	Sinovac	NO	3.327	52	I	74.597
P68	Female	21	165	47	17.264	Sinopharm	NO	3.32	42	I	81.073
P235	Female	23	165	53	19.467	Sinovac	NO	3.316	59	DD	63.787
P469	Female	23	165	60	22.039	NA	NO	3.306	72	I	68.098
P201	Female	36	162.5	52	19.692	Sinovac	NO	3.304	31	NA	74.748
P287	Female	37	156	42	17.258	Sinovac	NO	3.303	70	DD	47.439
P150	Female	24	167	57.5	20.617	Sinovac	YES	3.298	60	NA	73.788
P372	Female	21	158	56	22.432	Sinovac	NO	3.284	66	NA	64.529
P108	Female	23	157	45	18.256	Sinovac	NO	3.283	46	I	66.613
P369	Male	21	183	70	20.902	Sinopharm	NO	3.275	70	DD	69.841
P15	Female	23	158	44	17.625	NA	NO	3.274	38	DD	72.702
P113	Male	21	172	65	21.971	Sinopharm	NO	3.266	56	I	73.017
P226	Female	21	170	51	17.647	Sinopharm	NO	3.263	39	DD	72.452
P221	Female	21	166	60	21.774	Sinopharm	NO	3.258	38	DD	64.288
P291	Female	29	170	59	20.415	Sinopharm	NO	3.254	65	DD	70.545
P205	Female	20	168	52	18.424	Sinovac	NO	3.253	41	DD	72.594
P138	Female	21	165	50	18.365	Sinopharm	NO	3.248	56	I	60.986
P210	Female	22	158	45	18.026	Sinopharm	NO	3.233	40	I	76.487
P225	Female	20	160	50	19.531	Sinopharm	NO	3.22	62	DD	63.135
P439	Female	21	160	55	21.484	Sinovac	NO	3.203	72	I	75.360
P329	Male	26	174	60	19.818	Sinovac	NO	3.194	59	DD	71.071
P79	Female	24	155	45	18.730	Sinovac	NO	3.177	44	I	71.796
P244	Female	26	155	55	22.893	Sinovac	NO	3.172	54	DD	70.368
P288	Female	47	163	67.5	25.406	Sinovac	NO	3.17	58	I	73.345
P511	Female	23	172	75	25.352	Sinovac	NO	3.165	44	I	70.135
P94	Male	26	178	70	22.093	Sinovac	YES	3.16	102	I	68.689
P367	Male	27	186	95	27.460	Sinovac	NO	3.159	71	DD	63.286
P472	Female	45	153	56	23.922	Sinovac	NO	3.14	86	I	75.188
P173	Female	23	163	41.5	15.620	NA	NO	3.126	53	I	69.725
P106	Female	20	160	55	21.484	Sinovac	NO	3.122	74	NA	70.091
P117	Female	21	158	54	21.631	Sinopharm	NO	3.107	55	I	70.851
P186	Female	20	159	53	20.964	Sinovac	NO	3.102	40	I	NA
P246	Female	23	160	64	25.000	Sinopharm	NO	3.1	33	DD	NA
P532	Female	31	168	53	18.778	NA	NO	3.094	32	DD	NA
P57	Female	24	165	46	16.896	Sinopharm	NO	3.072	49	I	NA
P88	Female	23	165	50	18.365	Sinovac	NO	3.059	68	DD	46.736
P196	Male	20	170	56	19.377	Sinovac	NO	3.053	40	NA	53.372
P97	Male	22	180	67.5	20.833	Sinopharm	NO	3.051	49	I	NA
P177	Female	21	156	47	19.313	Sinovac	NO	3.049	53	I	NA
P277	Female	NA	162	85	32.388	Sinovac	NO	3.035	NA	I	NA

P130	Female	19	165	52	19.100	Sinovac	NO	3.031	52	NA	NA
P184	Male	20	178	68	21.462	Sinovac	NO	3.029	53	DD	NA
P37	Male	19	170	53	18.339	Sinopharm	NO	3.02	40	I	NA
P253	Male	24	180	95	29.321	Sinovac	NO	3.011	59	I	NA
P89	Male	20	174	50	16.515	Sinovac	NO	3.007	27	I	NA
P504	Female	19	159	47.5	18.789	NA	NO	2.996	70	I	NA
P105	Female	21	165	54	19.835	Sinovac	NO	2.978	28	NA	NA
P223	Male	23	182	66	19.925	Sinovac	NO	2.973	59	DD	NA
P3	Male	19	178	65	20.515	Sinovac	NO	2.97	38	I	NA
P183	Female	23	168	60	21.259	Sinopharm	NO	2.97	56	I	NA
P202	Female	33	170	72	24.913	Sinovac	YES	2.954	31	I	49.520
P142	Male	40	178	63	19.884	Sinovac	NO	2.951	75	I	NA
P189	Female	22	157	46	18.662	Sinovac	NO	2.951	53	DD	NA
P51	Male	21	182	75	22.642	Sinovac	NO	2.948	24	DD	NA
P297	Female	44	156	59	24.244	Sinovac	NO	2.943	29	DD	NA
P366	Female	44	165	77.5	28.466	Sinovac	NO	2.939	27	I	NA
P2	Female	19	165	57	20.937	Sinovac	NO	2.937	38	DD	NA
P295	Female	58	160	60	23.438	Sinovac	NO	2.933	135	I	53.874
P21	Female	24	163.5	52	19.452	Sinovac	YES	2.924	44	I	NA
P49	Male	28	177	65	20.748	Sinovac	YES	2.917	38	DD	NA
P140	Female	19	165	54	19.835	Sinovac	NO	2.914	33	I	NA
P101	Female	45	166	65	23.588	Sinovac	NO	2.908	106	DD	NA
P522	Female	21	165	56	20.569	Sinovac	NO	2.904	68	DD	NA
P209	Female	26	163	59	22.206	Sinopharm	NO	2.9	27	DD	NA
P170	Male	23	178	80	25.249	Sinovac	NO	2.889	52	I	NA
P236	Female	21	170	58	20.069	Sinopharm	NO	2.888	40	I	NA
P521	Female	22	161	52	20.061	Sinovac	NO	2.869	39	I	NA
P382	Female	64	168	65	23.030	Sinovac	NO	2.86	29	NA	NA
P190	Female	20	163	65	24.465	Sinovac	NO	2.853	40	I	NA
P176	Female	22	163	59	22.206	Sinopharm	NO	2.85	32	I	NA
P408	Male	51	165	70	25.712	NA	NO	2.845	122	I	NA
P41	Female	54	164	69	25.654	Sinopharm	YES	2.831	38	I	NA
P283	Female	20	167	55	19.721	Sinovac	NO	2.827	66	I	NA
P267	Female	19	165	49	17.998	Sinopharm	NO	2.822	39	DD	NA
P11	Female	22	163	80	30.110	Sinovac	NO	2.809	39	I	NA
P65	Male	25	178	64	20.199	Sinovac	NO	2.804	42	I	NA
P120	Female	22	165	64	23.508	Sinopharm	YES	2.799	50	DD	NA
P139	Male	29	175	63	20.571	Sinopharm	NO	2.786	43	I	NA
P188	Male	19	173	60	20.047	Sinovac	NO	2.78	52	DD	NA
P172	Female	23	166	52	18.871	NA	NO	2.774	52	DD	48.182
P483	Male	31	170	120	41.522	NA	NO	2.773	74	I	39.156
P121	Female	26	160	52	20.313	Sinovac	NO	2.765	9	I	NA
P126	Male	23	178	76	23.987	Sinopharm	NO	2.762	48	I	NA
P231	Male	18	186	65	18.788	Sinovac	NO	2.762	55	DD	NA
P217	Female	22	162	60	22.862	Sinopharm	NA	2.756	39	I	NA
P109	Female	25	163	50	18.819	Sinovac	NO	2.745	52	DD	NA
P66	Female	25	164	54	20.077	Sinopharm	NO	2.739	49	I	NA
P347	Female	26	151	59	25.876	Sinopharm	NO	2.734	39	DD	NA
P52	Male	22	174	90	29.727	Sinovac	NO	2.731	27	I	NA
P157	Female	24	161	54	20.833	Sinopharm	NO	2.727	57	I	NA
P229	Male	32	175	93	30.367	Sinovac	NO	2.715	68	DD	NA
P440	Male	22	175	84	27.429	NA	NO	2.709	42	I	NA
P477	Male	23	178	70	22.093	Sinovac	NO	2.706	74	I	NA
P60	Female	23	NA	NA	NA	NA	NA	2.705	49	I	NA
P502	Male	27	189	140	39.193	Sinopharm	NO	2.674	82	DD	NA
P34	Female	31	158	50	20.029	Sinovac	NO	2.672	45	I	NA
P381	Female	57	NA	NA	NA	Sinovac	NA	2.67	NA	NA	NA
P143	Male	25	175	60	19.592	Sinopharm	YES	2.651	48	DD	NA
P269	Female	21	165	58	21.304	Sinopharm	NO	2.647	35	I	NA
P48	Female	34	162	58	22.100	Sinovac	NO	2.643	54	I	NA
P87	Female	24	152	48	20.776	Sinovac	NO	2.643	45	DD	NA
P224	Female	19	172	57	19.267	Sinovac	NO	2.639	59	I	NA
P197	Male	21	168	70	24.802	Sinovac	NO	2.632	40	NA	NA
P506	Female	21	165	63	23.140	Sinovac	NO	2.616	44	I	NA
P256	Female	24	164	49	18.218	Sinovac	YES	2.6	56	I	NA
P533	Female	NA	NA	NA	NA	NA	NA	2.598	NA	DD	NA
P262	Male	24	175	63	20.571	Sinovac	NO	2.593	58	NA	NA
P476	Male	22	180	85	26.235	Sinovac	NO	2.591	51	I	NA
P503	Female	54	NA	NA	NA	Sinovac	NA	2.585	NA	I	NA
P62	Female	26	163	57	21.454	Sinopharm	NO	2.582	49	I	NA
P320	Male	27	183	68	20.305	Sinovac	YES	2.581	59	I	NA
P493	Male	NA	NA	NA	NA	NA	NA	2.581	NA	NA	NA
P78	Female	21	169	69	24.159	Sinovac	NO	2.567	44	I	NA
P362	Female	51	165	50	18.365	Sinovac	NO	2.566	28	DD	NA
P75	Female	22	163	48	18.066	Sinovac	YES	2.55	40	I	NA
P29	Female	22	166	52	18.871	Sinopharm	NO	2.543	49	I	NA
P436	Female	25	158	52	20.830	Sinovac	NO	2.527	73	DD	NA
P370	Female	57	160	58	22.656	Sinovac	NA	2.515	59	I	NA
P161	Male	74	165	60	22.039	Sinovac	NO	2.512	47	I	NA
P268	Female	20	163	60	22.583	Sinopharm	NO	2.503	35	I	NA
P73	Female	22	156	42	17.258	Sinopharm	NO	2.501	50	I	NA
P214	Female	20	160	62.5	24.414	Sinopharm	NO	2.49	38	I	NA
P55	Female	20	159	50	19.778	Sinopharm	NO	2.474	24	I	NA
P455	Female	23	164	59	21.936	Sinopharm	NO	2.465	41	I	NA
P507	Female	NA	NA	NA	NA	NA	NA	2.459	NA	DD	NA
P80	Male	22	177	68	21.705	Sinopharm	YES	2.446	50	I	66.115
P96	Male	21	172	70	23.661	Sinovac	NO	2.445	28	DD	NA
P39	Male	24	175	68	22.204	Sinopharm	NO	2.443	40	I	NA
P125	Female	21	166	57.5	20.867	Sinopharm	NO	2.442	57	I	NA
P216	Female	22	161	47	18.132	Sinopharm	NO	2.439	39	DD	NA
P135	Female	21	157	45	18.256	Sinovac	NO	2.426	34	I	NA
P490	Male	NA	NA	NA	NA	Sinopharm	NA	2.425	NA	NA	NA
P332	Female	64	155	55	22.893	Sinovac	YES	2.423	31	DD	NA
P92	Male	20	176	62.5	20.177	Sinovac	NO	2.414	27	I	NA
P374	Female	45	156	57	23.422	NA	NO	2.414	116	NA	NA
P430	Female	62	158	62	24.836	NA	NO	2.4	78	I	NA
P168	Male	27	187	90	25.737	Sinovac	NO	2.397	53	DD	NA
P153	Male	20	174	84	27.745	NA	NO	2.388	104	NA	NA
P207	Female	21	165	60	22.039	Sinopharm	NO	2.382	39	I	NA
P373	Female	21	163	51	19.195	Sinopharm	NO	2.375	94	NA	NA
P433	Female	25	166	52	18.871	NA	NO	2.374	37	I	NA
P517	Male	22	175	55	17.959	Sinovac	NO	2.37	70	I	NA
P134	Male	29	180	80	24.691	Sinovac	NO	2.367	62	I	NA
P112	Female	22	162	52	19.814	Sinovac	NO	2.358	50	I	NA
P43	Female	24	162	70	26.673	Sinovac	NO	2.354	44	I	NA
P111	Male	21	181	85	25.945	Sinovac	YES	2.35	52	I	NA
P42	Male	24	185	86	25.128	Sinovac	NO	2.346	44	I	NA
P481	Male	28	170	66	22.837	Sinovac	NO	2.335	74	I	NA
P488	Male	NA	NA	NA	NA	NA	NA	2.328	NA	NA	NA
P100	Female	31	155	59	24.558	Sinovac	NO	2.302	46	DD	NA
P218	Male	20	173	58	19.379	Sinovac	NO	2.3	38	DD	NA
P394	Male	28	175	80	26.122	Sinovac	NO	2.291	30	DD	NA
P415	Male	68	NA	65	NA	NA	NO	2.291	35	I	NA
P518	Female	41	160	57	22.266	Sinovac	NO	2.287	99	DD	NA
P147	Male	26	170	65	22.491	Sinopharm	NO	2.285	55	I	NA
P357	Female	25	150	48	21.333	Sinopharm	YES	2.284	46	I	22.661
P127	Female	22	163	45	16.937	NA	YES	2.284	48	I	NA
P286	Female	49	163	56	21.077	Sinovac	NO	2.274	120	I	NA
P115	Male	21	182	75	22.642	Sinovac	NO	2.268	52	I	NA
P368	Male	26	178	145	45.764	Sinovac	NO	2.261	65	I	NA
P118	Female	24	160	58	22.656	Sinovac	NO	2.257	52	I	NA
P484	Female	NA	NA	NA	NA	NA	NA	2.255	NA	I	NA
P59	Male	19	165	58	21.304	Sinopharm	NO	2.254	42	I	NA
P63	Male	22	176	90	29.055	Sinopharm	NO	2.254	49	I	NA
P414	Female	23	160	56	21.875	Sinopharm	NO	2.244	52	I	NA
P162	Male	21	178	54	17.043	Sinovac	NO	2.236	53	I	NA
P482	Male	22	172	61	20.619	Sinopharm	NO	2.236	78	I	NA
P501	Female	23	162	60	22.862	Sinopharm	NO	2.235	55	DD	NA
P304	Female	21	166	57	20.685	Sinovac	NO	2.234	34	I	NA

P31	Male	23	184	67	19.790	Sinopharm	NO	2.232	49	I	NA
P144	Male	27	165	69	25.344	Sinovac	NO	2.226	64	I	NA
P290	Male	24	170	70	24.221	Sinovac	NO	2.218	65	I	NA
P255	Male	23	172	88	29.746	Sinovac	NO	2.215	60	I	NA
P32	Male	55	174	80	26.424	Sinopharm	YES	2.214	39	DD	NA
P195	Female	21	158	49	19.628	Sinopharm	NO	2.214	55	NA	NA
P185	Male	34	175	75	24.490	Sinovac	NO	2.213	63	NA	NA
P462	Male	25	170	60	20.761	Sinovac	NO	2.205	68	I	NA
P434	Female	29	160	57	22.266	Sinovac	NO	2.204	62	I	NA
P228	Female	20	153	43	18.369	Sinopharm	NO	2.203	37	DD	NA
P371	Male	50	176	70	22.598	Sinovac	YES	2.193	75	DD	NA
P365	Male	35	178	95	29.984	Sinovac	NO	2.189	NA	I	NA
P454	Female	24	163	52	19.572	NA	NO	2.189	41	I	NA
P401	Female	25	162	47	17.909	Sinovac	NO	2.187	NA	I	NA
P136	Male	23	175	87.5	28.571	Sinovac	NO	2.178	111	I	NA
P344	Female	23	163	52.5	19.760	Sinopharm	NO	2.173	60	I	NA
P429	Female	26	165	53	19.467	Sinovac	NO	2.171	61	I	NA
P348	Female	62	160	55	21.484	NA	NO	2.17	59	DD	NA
P148	Male	22	190	80	22.161	Sinopharm	NO	2.169	48	NA	NA
P194	Male	25	180	81	25.000	Sinovac	NO	2.166	61	NA	NA
P514	Female	21	155	62	25.806	NA	YES	2.156	49	I	NA
P452	Male	60	165	75	27.548	NA	YES	2.154	66	I	NA
P270	Male	25	173	61	20.382	Sinopharm	NO	2.142	64	I	NA
P53	Male	22	NA	NA	NA	NA	NA	2.137	27	I	NA
P402	Male	22	177	100	31.919	Sinopharm	NO	2.131	77	I	NA
P192	Male	37	174	80	26.424	Sinovac	NO	2.123	69	I	NA
P179	Male	27	180	75	23.148	Sinovac	NO	2.115	107	DD	NA
P102	Male	46	180	78	24.074	Sinovac	NO	2.114	136	NA	NA
P358	Male	23	170	60	20.761	Sinovac	NO	2.113	66	DD	NA
P119	Male	46	160	75	29.297	Sinovac	NO	2.112	75	I	NA
P254	Male	66	175	71	23.184	Sinovac	NO	2.104	27	DD	NA
P298	Female	52	160	58.5	22.852	Sinovac	NO	2.093	39	I	NA
P531	Female	22	156	50	20.546	Sinovac	NO	2.093	70	I	NA
P349	Male	55	172	70	23.661	NA	NO	2.089	145	I	NA
P299	Male	21	178	80	25.249	Sinovac	NO	2.082	81	DD	NA
P204	Male	23	175	63	20.571	Sinovac	NO	2.08	59	DD	NA
P156	Female	24	168	60	21.259	Sinovac	NO	2.076	51	I	67.674
P491	Female	NA	NA	NA	NA	NA	NA	2.064	NA	NA	47.988
P243	Female	34	160	49	19.141	Sinovac	NO	2.058	88	DD	NA
P132	Female	20	153	60	25.631	NA	NO	2.056	52	I	NA
P324	Female	58	159	68	26.898	Sinovac	NO	2.049	39	DD	NA
P28	Male	22	184	94	27.765	Sinovac	NO	2.042	45	I	NA
P425	Female	45	162	62.5	23.815	Sinovac	NO	2.036	87	NA	NA
P237	Female	22	158	54	21.631	Sinopharm	NO	2.027	56	DD	NA
P505	Male	38	183	80	23.888	Sinovac	NO	2.027	35	DD	NA
P321	Female	25	162	50	19.052	Sinopharm	NO	2.025	38	DD	NA
P128	Female	29	158	51	20.429	Sinovac	NO	2.014	55	NA	NA
P40	Male	37	170	81	28.028	Sinovac	NO	2.004	68	DD	NA
P412	Female	NA	NA	NA	NA	NA	NA	2.003	NA	I	NA
P356	Female	22	160	50	19.531	Sinopharm	NO	1.998	70	I	NA
P311	Male	28	175	80	26.122	Sinovac	YES	1.992	62	I	NA
P241	Male	23	171	58	19.835	Sinopharm	NO	1.987	64	DD	NA
P500	Male	28	175	65	21.224	Sinovac	NO	1.974	86	DD	NA
P208	Female	27	156	54	22.189	Sinopharm	NO	1.97	27	I	NA
P200	Male	23	170	50	17.301	Sinopharm	NO	1.951	63	NA	NA
P409	Female	22	167	56	20.080	Sinovac	NO	1.951	95	I	NA
P211	Male	23	170	60	20.761	Sinopharm	NO	1.949	40	I	NA
P219	Female	21	168	65	23.030	Sinopharm	NO	1.939	39	I	NA
P400	Female	NA	NA	NA	NA	Sinovac	NA	1.931	NA	DD	NA
P428	Female	62	158	63	25.236	NA	NO	1.919	35	I	NA
P103	Male	20	NA	NA	NA	NA	NA	1.917	28	NA	NA
P530	Female	NA	NA	NA	NA	Sinovac	NA	1.913	NA	DD	NA
P104	Female	21	172	65	21.971	Sinovac	NO	1.911	79	NA	NA
P351	Female	54.5	165	65	23.875	Sinovac	NO	1.91	29	I	NA
P473	Female	59	165	70	25.712	NA	NO	1.907	35	I	NA
P302	Female	22	160	49	19.141	Sinopharm	NO	1.901	36	DD	NA
P76	Female	23	158	58	23.233	Sinopharm	YES	1.899	50	DD	NA
P180	Female	21	160	64	25.000	Sinovac	YES	1.894	69	I	44.524
P141	Female	41	166	52	18.871	Sinovac	NO	1.89	102	I	NA
P257	Male	27	173	65	21.718	Sinovac	NO	1.887	45	I	NA
P123	Male	23	173	58	19.379	Sinopharm	NO	1.884	49	I	NA
P110	Female	24	170	55	19.031	Sinovac	NA	1.874	111	I	NA
P293	Female	26	163	60	22.583	Sinopharm	NO	1.874	45	I	NA
P261	Male	22	181	80	24.419	Sinovac	NO	1.865	29	I	NA
P326	Male	43	172	85	28.732	Sinovac	NO	1.852	123	I	NA
P377	Male	52	172	60	20.281	Sinovac	NO	1.842	71	NA	NA
P220	Female	20	150	42	18.667	Sinopharm	NO	1.834	38	DD	NA
P54	Male	NA	NA	NA	NA	NA	NA	1.822	NA	I	NA
P310	Female	42	151	62	27.192	NA	NO	1.82	28	DD	68.024
P407	Female	61	160	74	28.906	NA	NO	1.809	43	DD	NA
P337	Female	25	160	44	17.188	Sinopharm	NO	1.802	33	I	NA
P529	Female	23	172	66	22.309	Sinovac	NO	1.801	70	I	NA
P159	Male	22	174	69	22.790	Sinopharm	NO	1.797	49	DD	NA
P285	Male	25	173	70	23.389	Sinopharm	NO	1.783	71	I	NA
P227	Female	21	167.7	74	26.313	Sinovac	NO	1.782	70	DD	NA
P359	Male	25	174	82	27.084	Sinopharm	NO	1.77	47	DD	NA
P64	Male	30	174	67	22.130	Sinovac	NO	1.768	45	I	NA
P266	Male	25	172	79	26.704	Sinopharm	NO	1.767	87	NA	NA
P340	Female	23	168	65	23.030	Sinovac	NO	1.757	60	DD	NA
P444	Female	53	155	55	22.893	NA	NO	1.752	35	I	NA
P284	Male	24	178	70	22.093	Sinovac	NO	1.734	65	DD	NA
P519	Female	23	172	54	18.253	Sinopharm	NO	1.733	80	DD	NA
P384	Male	64	172	80	27.042	Sinovac	YES	1.726	31	NA	NA
P222	Female	23	172	65	21.971	Sinovac	NO	1.719	59	I	NA
P160	Female	42	160	50	19.531	Sinovac	NO	1.717	76	NA	NA
P248	Male	22	181	108	32.966	Sinopharm	NO	1.717	57	DD	NA
P468	Male	58	168	72.5	25.687	NA	NO	1.713	132	I	42.836
P4	Female	19	157	50	20.285	Sinovac	NO	1.711	38	I	NA
P389	Male	52	170	75	25.952	Sinovac	NO	1.708	26	NA	NA
P330	Female	66	NA	NA	NA	Sinovac	NA	1.706	NA	DD	45.387
P426	Female	51	156	50	20.546	NA	NO	1.702	55	I	NA
P528	Female	23	163	66.5	25.029	Sinovac	NO	1.673	70	DD	NA
P275	Female	36	155	70	29.136	Sinovac	NO	1.67	NA	NA	NA
P81	Female	22	156	46	18.902	Sinopharm	YES	1.668	51	I	NA
P432	Male	58	165	124	45.546	NA	NO	1.663	35	I	NA
P346	Male	31	178	65	20.515	Sinovac	NO	1.662	97	I	NA
P312	Female	29	165	52	19.100	Sinopharm	NO	1.652	69	I	NA
P355	Female	53	164	65	24.167	Sinovac	NO	1.647	25	DD	NA
P524	Male	22	184	85	25.106	Sinovac	NO	1.645	33	DD	NA
P353	Male	37	172	85	28.732	Sinopharm	NO	1.638	46	I	NA
P212	Female	20	155	63	26.223	Sinopharm	NO	1.632	38	DD	NA
P50	Male	21	190	85	23.546	Sinovac	NO	1.628	59	I	NA
P479	Female	53	171	62	21.203	Sinovac	NO	1.618	83	I	NA
P464	Male	25	170	92.5	32.007	NA	NO	1.606	43	I	NA
P333	Female	23	161	47	18.132	NA	NO	1.602	47	I	NA
P8	Male	75	163	72	27.099	NA	NO	1.593	38	I	NA
P325	Female	56	160	52	20.313	Sinovac	NO	1.578	58	DD	NA
P182	Female	24	157	45	18.256	Sinopharm	NO	1.572	56	DD	NA
P352	Male	24	180	69	21.296	Sinovac	NO	1.57	67	I	NA
P20	Male	19	182	68	20.529	Sinovac	NO	1.569	38	I	NA
P305	Female	63	155	57.5	23.933	Sinovac	NO	1.567	5	DD	NA
P131	Male	24	175	75	24.490	Sinovac	NO	1.565	111	I	NA
P445	Female	26	168	59	20.904	NA	NO	1.56	77	I	NA
P46	Male	21	175	60	19.592	Sinopharm	NO	1.559	48	I	NA
P387	Male	23	174	80	26.424	NA	NO	1.559	26	NA	NA
P492	Male	NA	NA	NA	NA	NA	NA	1.553	NA	NA	NA
P309	Female	47	150	52.5	23.333	NA	NO	1.541	126	DD	NA
P424	Male	57	180	79	24.383	NA	NO	1.54	35	NA	NA
P339	Male	23	173	66	22.052	Sinopharm	NO	1.538	70	I	NA

P363	Female	56	170	56	19.37	NA	NO	1.538	120	I	NA
P181	Female	23	155	46	19.14	Sinopharm	NO	1.521	54	I	NA
P199	Female	23	161	44	16.97	Sinovac	NO	1.521	59	NA	NA
P279	Male	27	173	67	22.38	Sinovac	NO	1.521	64	DD	NA
P213	Female	19	155	70	29.13	Sinopharm	NO	1.516	38	I	34.592
P334	Male	20	183	75	22.39	NA	NO	1.515	71	I	NA
P240	Male	24	175	73	23.83	Sinopharm	NO	1.51	64	DD	NA
P47	Female	22	162	51.5	19.62	Sinovac	NO	1.507	44	I	NA
P508	Female	52	160	68	26.56	Sinovac	NO	1.496	144	DD	NA
P450	Female	28	158	47	18.82	NA	NO	1.465	54	I	NA
P278	Female	NA	157	52	21.09	Sinovac	YES	1.449	NA	I	NA
P282	Male	30	171	68	23.25	Sinovac	NO	1.449	64	I	NA
P193	Male	26	181	81	24.725	Sinopharm	NO	1.446	55	I	NA
P443	Female	63	159	75	29.66	NA	NO	1.446	47	I	NA
P419	Female	58	160	80	31.25	NA	NO	1.445	225	NA	NA
P410	Female	NA	NA	NA	NA	NA	NA	1.432	NA	DD	NA
P427	Female	22	160	50	19.531	NA	NO	1.432	52	I	NA
P421	Female	51	170	60	20.76	NA	NO	1.425	122	NA	NA
P460	Male	62	160	60	23.43	NA	NO	1.411	35	I	NA
P317	Female	25	160	53	20.70	Sinopharm	NO	1.4	31	DD	43.585
P451	Male	50	163	73	27.47	NA	YES	1.398	127	I	NA
P465	Female	40	160	63	24.60	NA	NO	1.394	30	I	NA
P431	Male	61	162	60	22.86	NA	NO	1.391	59	I	NA
P328	Female	54	162	62	23.62	Sinovac	NO	1.379	NA	DD	NA
P525	Female	20	160	50	19.53	Sinovac	NO	1.374	70	DD	NA
P345	Female	60	156	63	25.88	Sinovac	NO	1.359	136	DD	NA
P449	Male	70	173	60	20.04	NA	NA	1.356	NA	I	NA
P27	Male	22	NA	NA	NA	NA	NA	1.345	49	I	NA
P303	Female	31	163	55	20.70	Sinopharm	NO	1.34	69	DD	NA
P327	Female	66	159	69	27.29	Sinovac	NO	1.339	61	DD	38.347
P515	Female	23	172	56	18.92	Sinovac	NO	1.336	70	DD	NA
P164	Male	21	172	66	22.30	Sinopharm	NO	1.335	56	I	NA
P509	Female	22	162	46	17.52	Sinovac	NO	1.328	85	I	NA
P260	Male	23	170	55	19.03	Sinopharm	NO	1.323	64	DD	NA
P498	Male	59	175	78	25.46	Sinovac	NO	1.316	107	DD	NA
P441	Female	23	166	55	19.95	Sinopharm	NO	1.315	88	I	NA
P233	Male	30	175	NA	NA	Sinovac	NO	1.312	59	I	NA
P67	Female	22	157	45	18.25	Sinopharm	NO	1.307	49	I	31.932
P341	Male	21	174	69	22.79	Sinovac	NO	1.303	61	I	NA
P252	Female	21	169	51	17.85	Sinovac	NO	1.3	72	I	NA
P510	Female	58	159	58	22.94	Sinovac	NO	1.294	107	I	NA
P378	Female	66	161	65	25.07	NA	NA	1.288	37	NA	41.101
P438	Female	27	161	48.5	18.71	Sinovac	NO	1.275	73	DD	NA
P478	Female	40	168	62	21.967	Sinovac	NO	1.265	83	I	59.581
P416	Female	44	165	60	22.03	NA	NO	1.257	130	I	NA
P463	Male	57	172	70	23.66	NA	NO	1.255	NA	DD	NA
P18	Female	22	160	57.5	22.461	NA	NO	1.244	105	I	NA
P499	Female	34	160	48	18.75	Sinovac	NO	1.239	98	I	NA
P437	Female	22	163	45.5	17.12	NA	NO	1.238	77	I	NA
P331	Female	50	160	65	25.391	NA	NO	1.234	115	DD	NA
P263	Male	20	184	65	19.19	Sinopharm	NO	1.228	63	NA	NA
P467	Male	38	170	78	26.99	NA	NO	1.223	131	I	NA
P487	Female	51	155	52	21.64	NA	YES	1.222	123	I	NA
P392	Male	64	175	55	17.95	NA	NO	1.222	62	NA	NA
P470	Male	48	176	78	25.18	Sinovac	NO	1.22	84	I	NA
P152	Male	45	175	70	22.85	Sinovac	NO	1.219	65	NA	65.289
P174	Female	24	155	53	22.06	Sinovac	NO	1.215	112	I	43.068
P383	Female	55	157	62	25.15	Sinovac	NO	1.209	31	NA	35.342
P512	Male	27	174	65	21.46	Sinovac	NO	1.203	80	DD	NA
P375	Female	48	160	54	21.09	NA	NO	1.2	120	NA	NA
P417	Female	32	164	60	22.30	Sinovac	NO	1.195	94	I	NA
P145	Female	23	163	51	19.19	Sinopharm	NO	1.181	98	DD	NA
P388	Female	51	160	65	25.39	Sinovac	NO	1.176	50	NA	NA
P318	Male	28	174	90	29.72	Sinopharm	NO	1.174	46	DD	NA
P319	Male	18	173	64	21.38	Sinovac	NO	1.174	60	DD	NA
P122	Male	18	169	60	21.00	Sinopharm	NO	1.165	55	I	NA
P489	Male	NA	NA	NA	NA	NA	NA	1.157	NA	NA	37.198
P399	Female	24	175	62	20.24	Sinopharm	NO	1.15	77	I	NA
P496	Male	20	180	60	18.51	Sinovac	NO	1.148	70	DD	NA
P198	Female	23	167	55.5	19.90	Sinopharm	NO	1.138	62	NA	NA
P435	Male	23	175	58	18.93	NA	NO	1.138	44	I	38.940
P271	Female	42	153	62	26.48	Sinovac	NO	1.132	NA	I	NA
P343	Female	26	170	53	18.33	Sinopharm	NO	1.131	61	I	NA
P294	Female	55	160	65	25.39	Sinovac	NO	1.129	124	I	47.205
P390	Female	48	164	65	24.16	Sinovac	NO	1.126	124	NA	NA
P322	Female	48	150	50	22.22	Sinovac	NO	1.123	113	DD	NA
P335	Male	25	175	72	23.51	Sinopharm	NO	1.118	NA	I	NA
P494	Male	NA	NA	NA	NA	NA	NA	1.117	NA	NA	NA
P361	Male	25	178	67	21.14	Sinovac	NO	1.114	66	DD	NA
P395	Female	NA	NA	NA	NA	NA	NA	1.108	NA	I	NA
P95	Female	27	168	55	19.48	Sinovac	NO	1.106	105	DD	31.571
P447	Female	27	160	82	32.03	Sinovac	NO	1.105	72	I	NA
P171	Male	22	178	100	31.56	Sinopharm	NO	1.103	71	I	NA
P397	Male	22	170	65	22.49	Sinovac	NO	1.094	53	I	NA
P1	Male	19	170	95	32.87	Sinovac	NO	1.092	38	I	NA
P300	Female	45	160	63	24.60	NA	NO	1.089	39	I	36.632
P534	Female	NA	NA	NA	NA	NA	NA	1.081	NA	DD	NA
P466	Female	23	165	50	18.36	Sinopharm	NO	1.065	72	I	NA
P292	Female	49	162	75	28.57	Sinovac	YES	1.058	123	DD	NA
P413	Female	55	157	64	25.96	NA	NO	1.042	47	I	NA
P129	Female	22	160	48	18.75	NA	YES	1.032	56	I	NA
P301	Female	62	174	74	24.44	Sinovac	YES	1.029	45	I	45.104
P232	Male	28	178	95	29.98	Sinopharm	YES	1.024	81	DD	NA
P206	Female	25	170	55	19.03	NA	NO	1.019	NA	I	NA
P480	Male	20	185	105	30.67	Sinovac	NO	1.015	282	I	NA
P26	Male	19	184	76	22.44	Sinopharm	NO	1.007	42	I	39.960
P457	Male	55	172	70	23.66	NA	NO	0.992	152	I	63.601
P420	Male	24	173	70	23.38	Sinopharm	NO	0.991	53	NA	42.582
P379	Male	NA	NA	NA	NA	NA	NA	0.989	NA	NA	39.750
P338	Male	23	181	72	21.977	Sinopharm	NO	0.98	70	I	44.551
P398	Male	22	180	68	20.988	Sinovac	NO	0.971	73	I	57.197
P453	Female	24	165	60	22.039	Sinopharm	NO	0.964	41	I	59.635
P520	Male	25	180	70	21.605	Sinopharm	NO	0.963	72	I	36.583
P187	Male	27	173	70	23.389	Sinovac	NO	0.96	52	I	43.262
P474	Male	53	170	84	29.066	NA	NO	0.959	119	I	43.612
P396	Female	NA	NA	NA	NA	Sinovac	NA	0.953	95	I	33.197
P406	Female	NA	NA	NA	NA	NA	NA	0.951	NA	I	30.442
P448	Female	67	165	61	22.406	NA	YES	0.945	67	I	42.395
P458	Female	23	162	70	26.673	Sinopharm	NO	0.941	51	I	46.101
P264	Male	49	174	72	23.781	Sinovac	NO	0.937	71	NA	30.642
P314	Female	38	159	50	19.778	Sinovac	NO	0.936	72	I	33.739
P526	Male	27	178	78	24.618	Sinovac	NO	0.932	NA	DD	41.787
P61	Female	48	158	53	21.231	Sinovac	NO	0.925	58	DD	56.372
P149	Female	28	162	58	22.100	Sinovac	NO	0.905	113	NA	40.017
P471	Male	44	179	60	18.726	NA	NO	0.847	132	I	31.692
P336	Female	25	173	60	20.047	Sinovac	NO	0.842	230	DD	53.335
P315	Male	28	176	68	21.952	Sinopharm	NO	0.836	59	I	46.438
P239	Female	51	162	60	22.862	Sinovac	NO	0.819	118	DD	35.006
P249	Male	23	175	65	21.224	Sinopharm	NO	0.818	63	I	33.258
P360	Male	36	175	62.5	20.408	Sinovac	NO	0.794	125	I	49.524
P391	Male	57	169	73.9	25.874	Sinovac	NO	0.775	71	NA	44.248
P405	Female	29	158	59	23.634	NA	NO	0.773	53	DD	35.265
P459	Female	50	162	70	26.673	NA	NO	0.754	128	I	40.310
P323	Female	42	160	67	26.172	Sinovac	NO	0.749	123	DD	48.905
P308	Male	54	172	85.5	28.901	Sinovac	NO	0.743	79	I	56.244
P523	Male	31	175	71	23.184	Sinopharm	NO	0.737	87	DD	36.466
P354	Female	24	160	59	23.047	Sinovac	NO	0.732	35	DD	39.558
P316	Male	25	176	75	24.212	Sinopharm	NO	0.726	59	DD	63.657
P45	Male	29	178	78	24.618	Sinovac	NO	0.689	104	DD	45.017

P274	Female	42	171	68	23.255	NA	NA	0.656	NA	DD	44.170
P386	Female	58	156	70	28.764	Sinovac	NO	0.611	12	NA	39.653
P178	Male	24	170	66	22.837	NA	NO	0.608	109	DD	59.258
P166	Male	24	177	60	19.152	Sinovac	YES	0.591	106	DD	51.775
P313	Male	50	170	110	38.062	Sinovac	NO	0.582	123	I	45.503
P307	Female	42	165	70	25.712	Sinovac	NO	0.545	151	I	46.914
P393	Male	50	175	82	26.776	Sinovac	NO	0.544	124	NA	57.616
P461	Female	23	165	56	20.569	Sinopharm	NO	0.523	72	I	50.415
P513	Male	31	173	84	28.066	Sinovac	YES	0.485	NA	I	32.478
P342	Female	50	155	55	22.893	Sinovac	NO	0.454	116	DD	45.802
P411	Female	27	164	62	23.052	NA	NO	0.447	83	I	57.539
P30	Male	59	181	82.2	25.091	Sinovac	YES	0.43	26	I	39.077
P151	Female	25	153	43	18.369	Sinovac	NO	0.419	113	NA	65.074
P442	Male	61	162	60	22.862	NA	NO	0.404	59	I	47.566
P527	Male	NA	NA	NA	NA	Sinovac	NA	0.401	NA	DD	61.963
P456	Female	37	162	62	23.624	Sinovac	NO	0.373	124	I	44.867
P385	Female	58	155	75	31.217	Sinovac	NO	0.364	133	NA	44.674
P497	Male	NA	NA	NA	NA	Sinovac	NA	0.328	NA	DD	39.889
P36	Female	22	165	47.5	17.447	Sinopharm	NO	0.321	39	DD	34.689
P422	Male	27	175	70	22.857	Sinopharm	NO	0.316	50	NA	38.579
P475	Male	66	169	110	38.514	NA	NO	0.283	6	I	39.982
P446	Female	27	155	58	24.142	Sinopharm	NO	0.277	78	I	42.799
P418	Female	24	165	52	19.100	Sinopharm	NO	0.267	66	NA	43.473
P306	Male	57	170	75	25.952	Sinopharm	NO	0.215	81	DD	64.755

Supplementary Table 2. Clinical information of patients with neutralization assay

Subject ID	Antibody level	Group	Neutralizing titer	Inhibition(%)
P14	5.043	High	32	62.87913305
P33	4.864	High	32	64.66141196
P35	4.82	High	24	74.48002059
P12	4.82	High	24	76.87926124
P90	4.797	High	16	77.00129438
P114	4.71	High	24	83.80303203
P44	4.707	High	16	64.83127621
P6	4.564	High	24	68.56043143
P17	4.552	High	16	86.6535316
P16	4.513	High	16	79.35947875
P13	4.512	High	24	91.07486458
P133	4.423	High	24	66.19453998
P7	4.42	High	16	74.63536587
P350	4.41	High	12	73.07487304
P85	4.323	High	16	78.01812809
P93	4.32	High	16	79.88637335
P376	4.296	High	12	71.51725168
P38	4.227	High	12	67.60225506
P5	4.211	High	12	81.824429
P230	4.181	High	16	82.11211854
P22	4.114	High	12	69.7287628
P9	4.111	High	16	67.07289327
P91	4.109	High	16	78.47979406
P23	4.081	High	12	71.52478634
P83	4.038	High	16	63.65588343
P378	1.288	Low	< 8	41.10066568
P383	1.209	Low	< 8	35.34203599
P435	1.138	Low	< 8	38.94044128
P300	1.089	Low	< 8	36.63163608
P301	1.029	Low	< 8	45.10449958
P26	1.007	Low	< 8	39.96007749
P354	0.732	Low	< 8	39.55752189
P386	0.611	Low	< 8	39.65256821
P36	0.321	Low	< 8	34.68925323
P475	0.283	Low	< 8	39.98165749

Supplementary Table 3. Clinical information of 26 subjects with PBMC RNA-Seq data.

Subject ID	Gender	Age	Height	Weight	BMI	Brand name of inactivated SARS-CoV-2 vaccine	Family history of cardiovascular diseases	Days after 2nd dose of inactivated SARS-CoV-2 vaccine	Antibody level	ACE Genotype	Group	Inhibition (%)
P35	Female	21	166	53	19.2336	Sinopharm	YES	56	4.82	NA	High	74.480
P14	Male	23	181	65	19.8407	Sinovac	NO	81	5.043	I	High	62.879
P16	Male	25	175	76	24.8163	Sinovac	NO	51	4.513	I	High	79.359
P17	Male	21	180	70	21.6049	Sinovac	NO	46	4.552	I	High	86.654
P22	Female	20	162	55	20.9572	NA	NO	70	4.114	I	High	69.729
P23	Female	22	165	67	24.6097	Sinopharm	NO	49	4.081	I	High	71.525
P33	Female	47	158	62	24.8358	Sinovac	NO	63	4.864	NA	High	64.661
P38	Female	60	164	65	24.1672	Sinovac	NO	59	4.227	DD	High	67.602
P44	Female	21	163	65	24.4646	Sinovac	NO	59	4.707	I	High	64.831
P12	Female	19	165	54	19.8347	Sinovac	NO	70	4.82	I	High	76.879
P5	Female	19	163	49	18.4425	Sinovac	NO	47	4.211	I	High	81.824
P6	Male	23	181	72	21.9774	Sinovac	NO	56	4.564	I	High	68.560
P7	Female	23	168	80	28.3447	Sinovac	NO	52	4.42	NA	High	74.635
P9	Male	21	164	51	18.9619	NA	NO	70	4.111	DD	High	67.073
P301	Female	62	174	74	24.4418	Sinovac	YES	45	1.029	I	Low	45.104
P30	Male	59	181	82.2	25.0908	Sinovac	YES	26	0.43	I	Low	39.077
P378	Female	66	161	65	25.0762	NA	NA	37	1.288	NA	Low	41.101
P36	Female	22	165	47.5	17.4472	Sinopharm	NO	39	0.321	DD	Low	34.689
P26	Male	19	184	76	22.4480	Sinopharm	NO	42	1.007	I	Low	39.960
P300	Female	45	160	63	24.6094	NA	NO	39	1.089	I	Low	36.632
P354	Female	24	160	59	23.0469	Sinovac	NO	35	0.732	DD	Low	39.558
P383	Female	55	157	62	25.1532	Sinovac	NO	31	1.209	NA	Low	35.342
P386	Female	58	156	70	28.7640	Sinovac	NO	12	0.611	NA	Low	39.653
P435	Male	23	175	58	18.9388	NA	NO	44	1.138	I	Low	38.940
P453	Female	24	165	60	22.0386	Sinopharm	NO	41	0.964	I	Low	59.635
P475	Male	66	169	110	38.5141	NA	NO	6	0.283	I	Low	39.982

Supplementary Table 4. Up-regulated genes in serum of subjects with high vs no anti-Spike RBD antibodies.

Gene	baseMean	log2FoldChange	lfcSE	stat	pvalue
PTGER3	31.92761582	1.666299148	0.47109225	3.537097346	0.000404551
WNT8B	1.934566037	1.630155359	0.64235722	2.537770743	0.011156104
RAP1GAP	3.528614919	1.209470538	0.511457576	2.364752416	0.018042128
DAZL	2.575017849	1.820772636	0.688198091	2.645710096	0.008151965
DSP	12.95912505	1.350010646	0.613515845	2.200449517	0.027775018
TCL1A	251.4136853	1.08930188	0.283671025	3.840018138	0.000123025
NFATC4	3.82005837	1.219403888	0.578449274	2.108056737	0.035026082
SALL4	3.638595741	1.147087828	0.517951179	2.214663999	0.026783136
SRPX	1.780255048	2.026743608	0.73815171	2.74570062	0.006038184
MINDY4	1.237464185	1.888786733	0.903747272	2.089950135	0.036622279
SERPINC1	2.346930482	1.644617504	0.629096565	2.614252875	0.008942282
MMP8	3.519125962	1.131805351	0.567673961	1.993759498	0.046178344
RASSF8	7.169565402	1.062583593	0.419702561	2.531753896	0.011349362
PRRG1	4.344112369	1.088778085	0.466754085	2.332658929	0.019666053
CCNA1	3.244746147	1.353065585	0.591022041	2.289365692	0.022058114
UNC79	4.231288761	1.31945745	0.470528522	2.804202912	0.005044115
CRB1	1.293988131	1.937743098	0.81136489	2.388251109	0.016928771
ARHGAP20	2.912856631	1.93421473	0.639822521	3.023048842	0.002502419
FRAS1	1.765341058	1.769856576	0.870701777	2.032678263	0.042085039
FCRL5	117.3045034	1.1512851	0.329263623	3.496545074	0.000471325
EFNA3	5.408010143	1.037319895	0.494732709	2.096727943	0.036017665
WDR31	3.868861704	1.133097517	0.536086935	2.113645089	0.034545592
CCDC3	6.196111495	1.686479905	0.567224888	2.973212108	0.002947007
SCN3A	22.29093488	1.023373763	0.32174548	3.18069352	0.00146923
ANKRD61	3.118826245	1.037517122	0.52888301	1.961713841	0.049795809
ZBTB8A	4.710429925	1.076416289	0.452313218	2.379802859	0.017321902
CAGE1	1.462122543	1.90358224	0.892124628	2.133762684	0.032862212
GFRA2	33.77821754	1.147073104	0.263731193	4.349402472	1.37E-05
CFAP46	3.865935528	1.158676262	0.523183161	2.214666579	0.026782958
HSPA6	905.9610501	1.831386251	0.59365911	3.084912234	0.002036122
ZBED2	8.004230784	1.007344599	0.412481319	2.44215811	0.014599751
AL672207.1	1.84444556	1.358959115	0.674400175	2.015063408	0.043898006
ACTG1P1	2.250044544	1.454824302	0.650759469	2.235579153	0.025379351
ZNF572	5.700995564	1.397868033	0.456736592	3.060556255	0.002209263
GPR173	2.591266314	1.269205162	0.567823847	2.235209334	0.025403609
PKP3	1.673307711	1.338520715	0.645659953	2.073104751	0.038162532
SORCS2	15.13667258	1.136949217	0.372854532	3.049310439	0.002293673
SPDYE16	1.669376432	1.967840929	0.793137407	2.481084504	0.013098332
EFCAB10	1.933931947	1.470509166	0.66610666	2.20761817	0.027270901
FAM228A	2.078470857	1.5370921	0.650382186	2.36336747	0.018109703
TMEM221	2.524830017	1.184588601	0.55111387	2.149444362	0.031599192
TREML4	6.762655865	3.046746567	0.832464176	3.65991313	0.000252301
NANOS1	2.860106699	1.282315147	0.590574815	2.171300086	0.029908496
HRH1	2.18259131	2.850439022	1.033585266	2.757817004	0.005818876
GYPE	3.810981428	1.403530119	0.445261763	3.152146078	0.001620752
SLC2A10	3.017098839	1.202804408	0.571465325	2.104772338	0.03531113
HOXA4	1.624797819	1.70706807	0.831606873	2.052734442	0.04009834
STPG3	1.426901447	1.654046827	0.817534551	2.023213361	0.043051155
KCNRG	4.111118164	1.484390965	0.437543455	3.392556668	0.000692436
SNORA16B	1.703846812	2.156706999	0.903006876	2.388361656	0.016923679
IGBP1-AS1	1.418777146	2.143786276	0.837169358	2.560755785	0.010444475
GGNBP1	3.292818418	1.085202785	0.519567975	2.088663732	0.036738004
HLA-DOA	88.98165884	1.986267882	0.512887731	3.872714748	0.00010763
TRIM51BP	7.052373856	1.756860541	0.661104845	2.657461299	0.007873163
AC118344.1	2.814741743	1.394918597	0.622501417	2.240827985	0.025037219
AC118278.1	2.481736159	1.611266662	0.650241144	2.477952491	0.013213874
IGLV3-12	5.136055398	1.514242338	0.642493001	2.356823086	0.018432026

TRGJP	2.82613995	1.134025558	0.536988689	2.111823919	0.034701555
IGHD3-22	2.845983286	2.202297022	0.748062551	2.944001165	0.003239988
IGHD3-9	5.270417846	1.509831524	0.459651794	3.284728887	0.001020805
IGHV3-13	14.84060834	1.05198026	0.364732439	2.884251983	0.003923447
IGHV3-48	36.80992688	1.020493124	0.386737658	2.638721891	0.008321922
IGHV3-53	26.35347448	1.295349541	0.41194717	3.144455496	0.001663962
IGHV5-78	7.475600715	1.087735514	0.41120301	2.645251827	0.008163014
AC010615.1	4.734564791	1.935288593	0.515980892	3.750698181	0.000176343
AC010468.1	3.419339446	1.135451774	0.480134405	2.364862344	0.018036774
AC114744.1	4.907465533	1.126924983	0.538492966	2.092738539	0.036372501
UBE2QL1	1.628306559	1.652121515	0.826318343	1.999376548	0.045567627
GAB4	2.168039698	2.598606478	0.812902848	3.19669993	0.001390095
AC005481.1	1.826368705	1.670434298	0.801730345	2.083536326	0.037202364
DCDC2B	0.96866178	2.065872025	0.938281562	2.201761293	0.027682175
AC022400.1	5.688196168	1.067774339	0.380873481	2.803488275	0.005055307
IGHV3-74	70.0907854	1.019387096	0.332349919	3.067210306	0.002160668
DYNC1I2P1	2.123672501	1.347037735	0.637536751	2.112878566	0.034611163
IGHD6-25	2.798316988	1.653249604	0.680506099	2.429441274	0.015122115
ZNF503-AS1	1.368882113	2.344735594	0.8897039	2.635411167	0.008403542
AC079779.1	5.176064015	1.377441064	0.454781164	3.028799724	0.002455274
AL080276.2	3.151749104	1.368393303	0.562032116	2.434724394	0.014903136
SUCLA2-AS1	1.768247476	1.627222946	0.672014058	2.421412064	0.015460341
AL096678.1	0.92013735	1.964491008	0.871301134	2.254663667	0.024154448
RAB11FIP1P1	1.738158656	2.537899735	0.872900011	2.907434646	0.003644065
BTBD6P1	5.996000463	1.098198045	0.35366322	3.10520852	0.001901449
AC233976.1	3.626895774	1.119582859	0.5709709	1.960840488	0.049897633
ACAP2-IT1	3.024092002	1.280127597	0.624915401	2.048481434	0.040512849
LINC01315	7.284312384	1.319817898	0.361628207	3.649654186	0.000262594
LRRC37A15P	2.692540318	1.562213145	0.689181832	2.266764841	0.023404595
CNOT6LP1	1.536432646	1.553983093	0.721038978	2.155199845	0.031146199
DSCR9	2.427137827	1.159541174	0.557683916	2.079208565	0.037598185
YY2	3.846414295	1.032236304	0.501538588	2.05813935	0.039576759
LINC01293	1.365730863	2.093020734	0.942263285	2.221269541	0.026332711
BEND3P1	11.48644953	1.291876384	0.410948771	3.14364338	0.001668587
RP6-206I17.2	3.255448845	1.170314715	0.576309318	2.030705869	0.042284841
AL589935.1	3.813989266	1.083277185	0.509202139	2.127401089	0.033386772
DPYD-IT1	2.643180561	1.400928349	0.527145486	2.657574401	0.007870522
AC060234.2	2.049592929	1.568279326	0.665949213	2.354953343	0.018525032
LINC01694	2.264946376	1.723597839	0.627047051	2.748753603	0.005982234
AC073349.2	8.325155045	1.185941867	0.533529547	2.222823224	0.026227722
HM13-IT1	2.95354783	1.425110819	0.532394434	2.676795113	0.007433007
SNORA71B	4.607599579	1.33886685	0.537598434	2.490458986	0.012757822
AC104135.1	2.975641181	1.747617172	0.708238334	2.467555183	0.013603925
NDUFAF4P3	1.52350115	1.517726408	0.754153835	2.012489147	0.0441684
AC097662.1	4.563943569	1.223580818	0.408589014	2.994649335	0.002747605
AL034550.1	2.798542249	1.600087426	0.654412259	2.445075566	0.014482179
AL357079.1	5.934881005	1.269881848	0.504597237	2.516624654	0.011848498
Z68871.1	4.016278513	1.305650338	0.480248472	2.718697535	0.006553951
JMJD7	2.850837735	1.229346761	0.601176734	2.04490076	0.040864643
AC246787.2	10.33995469	1.149837821	0.289465501	3.972279317	7.12E-05
AL449212.1	4.245084734	1.374678657	0.581820709	2.362718679	0.018141435
AC104958.1	2.55262021	2.250868735	0.912250061	2.467381293	0.013610534
TARM1	2.944104828	1.791148688	0.879408456	2.036765368	0.04167356
MTND6P4	1.902418492	1.569582839	0.699621846	2.243473169	0.024866318
OSGEPL1-AS1	2.738468235	1.143633694	0.557052525	2.053008726	0.040071731
AC011726.3	1.565175794	1.646155158	0.795490838	2.069357784	0.038512525
AC080023.2	1.010086951	1.840842562	0.886284381	2.077033739	0.037798445
PABPC1P4	2.781540049	1.704991689	0.58269541	2.926042767	0.003433037
AC112777.1	2.815985883	1.014672933	0.504059435	2.01300256	0.04411436

OTOAP1	2.915050753	1.276142378	0.609813595	2.092676171	0.036378072
AL442663.3	2.240642844	1.566725044	0.66454171	2.357602271	0.018393388
AL355075.5	7.291004824	1.01064102	0.393154432	2.570595515	0.010152383
SLC22A31	5.220143761	1.003559997	0.502451135	1.99732855	0.045789504
AL513534.2	3.058612008	1.32444659	0.656774049	2.016593975	0.043737902
AL031709.1	3.69999238	1.014986573	0.492740911	2.059878835	0.039410126
AC092338.2	2.824473098	1.864493632	0.631206866	2.95385512	0.003138313
EPPK1	16.58375455	1.268472188	0.443439884	2.860527961	0.004229363
AC006504.1	3.624193484	1.167143468	0.589735108	1.979097823	0.047804994
AC004034.1	1.875452271	1.399701652	0.625268684	2.238560299	0.02518454
AC104564.3	9.654966893	1.101030618	0.392105981	2.807992411	0.00498514
hsa-mir-4539	2.477090787	1.149528528	0.566111779	2.030568115	0.042298825
AC022211.3	1.858297194	1.899469553	0.839068552	2.263783511	0.023587433
AP005671.1	0.93486314	2.004362135	0.937015244	2.139092344	0.03242819
AL122127.4	4.13682411	1.080251757	0.488185293	2.212790455	0.026912096
AC010618.3	1.160713489	2.06978438	0.917264536	2.256474876	0.024040907
AC078795.1	1.813653451	1.897296034	0.816679686	2.323182597	0.020169345
AL583832.1	3.088406039	1.554967338	0.609821366	2.549873496	0.010776201
AC090948.2	21.71954575	1.214810392	0.348371399	3.487112881	0.000488265
AC084018.1	15.34371428	1.459642174	0.506284168	2.883049215	0.003938459
AC009237.14	1.632279252	1.833702975	0.824365063	2.224382204	0.02612274
AC084824.3	2.218445873	1.323869264	0.607494194	2.179229494	0.029314624
AC092119.2	3.917796009	1.501392232	0.68503843	2.191690518	0.028401861
LINC02371	1.188104258	1.82734148	0.851464785	2.146115155	0.031863792
AC134878.2	3.980245023	1.212878097	0.603081065	2.011136091	0.044311084
AC100847.1	1.139244036	2.016526858	0.88821073	2.270324811	0.023187883
AC020951.1	3.619176134	1.86221444	0.760632041	2.448246117	0.014355356
AC099667.1	1.682436635	1.898629664	0.817031349	2.323814949	0.020135414
AC093503.4	1.901916586	1.818022431	0.832596862	2.183556669	0.028994841
AP000866.6	4.170268363	1.292147619	0.62361061	2.07204239	0.038261488
AC007610.4	2.615105277	1.922535928	0.783602381	2.453458509	0.014148986
AC003957.1	2.914593768	1.425572154	0.683713737	2.085042434	0.037065449
AC004241.5	2.891543039	1.000528868	0.476613864	2.099244156	0.035795383
AL603750.1	2.8242575	2.256881615	0.685608603	3.291793024	0.000995508
AC093642.2	3.937191527	1.137110941	0.541544937	2.099753618	0.03575052
AC096887.2	1.176149048	1.81161586	0.915410984	1.979019142	0.047813851
AC004706.3	10.12668248	1.09667719	0.408716279	2.683223661	0.007291621
Z95115.2	1.260949733	1.94549309	0.860988665	2.259603603	0.023845864
AC119673.2	2.388180609	1.541846797	0.772047958	1.997086813	0.045815753
AL137139.1	6.578060239	1.066679181	0.497008821	2.146197684	0.03185721
AL133313.1	1.256639915	2.226265892	0.934913677	2.381252888	0.017253863
AC022137.4	4.984371259	1.170627652	0.505694534	2.314890855	0.020618906
AC006059.5	1.948480743	1.982637241	0.768574455	2.579629374	0.00989064

Supplementary Table 5. Down-regulated genes in serum of subjects with high vs no anti-Spike RBD antibodies.

Gene	baseMean	log2FoldChange	lfcSE	stat	pvalue
SLC4A1	42.76104562	-1.284095956	0.562132035	-2.284331575	0.022352052
RHBDF1	1.967550922	-1.373523246	0.616371418	-2.228401914	0.025853726
CLDN11	1.344755471	-1.554233436	0.754739544	-2.05929774	0.039465725
PITX1	2.063892247	-1.630401618	0.701127697	-2.325398961	0.020050637
GLI2	1.511666964	-2.429418961	0.797610377	-3.045871807	0.002320067
KCNH4	4.222358552	-1.353662632	0.479621122	-2.822358254	0.004767189
PLEK2	4.049878541	-1.216456515	0.467229363	-2.603553222	0.009226294
NALCN	1.243390765	-1.945892834	0.831005986	-2.341611093	0.019200711
LHB	3.47406593	-1.100523016	0.52496667	-2.096367404	0.036049612
RSPH6A	5.337910524	-1.306850605	0.47491263	-2.751770586	0.005927403
CRX	3.820176384	-1.03613154	0.523062611	-1.980893908	0.047603172
RASIP1	25.71889737	-2.405196825	0.532271376	-4.518741633	6.22E-06
UPK1A	1.303530458	-1.896655179	0.798742881	-2.374550339	0.01757034
KCNT1	15.02657935	-1.093159731	0.357830526	-3.054964998	0.002250868
RUNDC3A	5.749689354	-1.400084104	0.552057684	-2.536119221	0.011208857
FAM149A	4.557429309	-2.079304093	0.561317512	-3.704327852	0.000211952
IL1A	42.66877864	-2.309838117	0.703243445	-3.284549799	0.001021454
CCL20	137.8994543	-2.388880309	0.621299109	-3.844976234	0.000120564
PRKAG3	1.22314201	-2.62274024	0.821686155	-3.191900247	0.001413401
RBPJL	1.599120964	-1.486329909	0.68768797	-2.161343477	0.030668817
HIF3A	1.992260665	-1.267843803	0.628959109	-2.015780969	0.043822884
HIST1H4B	3.559909226	-1.46684201	0.50708373	-2.892701782	0.003819438
IL1B	4996.511211	-1.308453125	0.471300958	-2.776258147	0.005498853
BPIFB1	1.998642578	-1.842323797	0.675783305	-2.726204959	0.00640672
FFAR2	207.2059708	-1.345093924	0.314251203	-4.280314315	1.87E-05
VGF	3.147729749	-1.173591008	0.593890503	-1.97610671	0.048142693
UGT2B28	3.592659525	-1.575859056	0.706345638	-2.231002743	0.025680947
IL1RN	1352.018006	-1.403365988	0.447963966	-3.132765342	0.001731678
ALDOB	1.095932501	-2.162980432	0.813609905	-2.658498157	0.007848978
TRPC3	6.742184592	-1.25562366	0.475487067	-2.640710438	0.00827324
SLC39A5	2.441520357	-1.171756526	0.510293605	-2.296239881	0.021662169
SLIT2	1.439138542	-2.030141736	0.88102335	-2.304299579	0.021205833
TPBG	18.65037867	-1.564792931	0.398920718	-3.922566216	8.76E-05
VWDE	2.651361466	-1.944440481	0.770281198	-2.524325514	0.011592054
SNTG1	2.257881584	-2.018288043	0.732681936	-2.754657845	0.005875354
NPAS3	1.519725144	-2.058688477	0.801567313	-2.568328879	0.010219015
DNAAF1	18.55988581	-1.340034577	0.537434241	-2.493392631	0.012652885
ABI3BP	1.216435903	-1.487308198	0.717330971	-2.073391861	0.038135826
CDC25C	13.73559465	-1.211882833	0.393849785	-3.07701789	0.002090828
KCNB1	2.523131103	-1.411353784	0.654997409	-2.154747124	0.031181628
ALAS2	187.8404242	-2.971195559	0.70474794	-4.215969131	2.49E-05
NPM2	5.345112007	-1.152363371	0.490315494	-2.350248738	0.018760869
COL26A1	8.200742329	-2.157676092	1.002378614	-2.152555993	0.03135359
SNX7	5.223847049	-2.849111664	0.74384191	-3.83026504	0.000128005
C1orf189	1.681916342	-1.679878296	0.721228115	-2.329191363	0.01984893
PTX3	345.3657462	-1.231958564	0.361763947	-3.405421057	0.000660621
CXCL3	68.25548399	-1.061469727	0.468129536	-2.2674701	0.023361523
CPA3	13.90246413	-1.099967562	0.418820202	-2.62634791	0.008630655
IFI27	29.25881006	-1.117283683	0.528908122	-2.112434348	0.034649212
RRAD	83.28036487	-1.116613744	0.475604623	-2.347777313	0.018885809
CA7	6.571958401	-1.584008018	0.391763504	-4.043276114	5.27E-05
SLC35G2	11.0329776	-1.16395673	0.442337018	-2.631379884	0.008503892
CXCL10	68.25050101	-1.304954716	0.451879937	-2.887835043	0.003879032
PCDH7	1.311377198	-1.90205949	0.82406452	-2.308143894	0.020991134
AHSP	19.43775217	-1.673081264	0.82985666	-2.016108738	0.043788607
DLGAP1	5.007413141	-1.258892553	0.530833054	-2.371541379	0.017714063

HOXB9	2.180287331	-1.35212956	0.625709509	-2.160954152	0.030698881
LRG1	127.8498619	-1.019819736	0.399475707	-2.552895501	0.010683154
RCAN2	30.36417152	-1.592438905	0.563250695	-2.827229366	0.004695268
IGDCC3	2.790844241	-1.158030008	0.50270898	-2.303579315	0.02124627
NKX2-5	3.96776399	-1.225951636	0.49124333	-2.495609732	0.012574086
EPHA10	2.579955329	-1.25793872	0.509747912	-2.467766305	0.013595905
GRIN2A	2.08321165	-2.019819224	0.775603621	-2.604190039	0.009209168
HIST2H2AA3	2.517509737	-1.236982303	0.528507963	-2.340517814	0.01925702
HIST2H2AB	17.18049101	-1.456669014	0.350938699	-4.150779091	3.31E-05
IRS3P	3.507151269	-1.261763386	0.511672116	-2.465960813	0.013664628
ZBTB7C	7.362838838	-1.039787706	0.467918092	-2.222157515	0.026272662
FFAR3	9.842457622	-1.031067308	0.501328154	-2.056671464	0.039717839
SELENOV	1.747921948	-1.922529053	0.748460506	-2.56864462	0.01020971
CSPG4P8	2.131031521	-1.413050754	0.61348657	-2.303311636	0.021261315
PRTN3	5.971968368	-1.424563438	0.624914572	-2.279613092	0.022630646
SLC30A10	2.359285335	-1.625297406	0.629417329	-2.582225387	0.009816545
CFAP43	1.84512217	-1.334359044	0.664663881	-2.007569664	0.044689041
KEL	4.324587407	-1.275111776	0.46914649	-2.717939501	0.006568985
PAX9	2.147718425	-1.919981355	0.879512292	-2.183006845	0.029035306
RNU5F-1	13.4035873	-1.365651865	0.61309689	-2.227465002	0.025916212
RNU5A-1	25.08944965	-1.202069657	0.392744907	-3.060688081	0.00220829
VTRNA1-1	15.37931478	-2.065434193	0.576779429	-3.580977558	0.000342311
RNU5B-1	8.167221501	-1.394429662	0.517609283	-2.69398117	0.007060415
RNU1-59P	32.61443178	-1.68580263	0.380163632	-4.434413201	9.23E-06
RNU5E-4P	21.92513434	-1.837475133	0.594752448	-3.089478892	0.00200508
RNU1-60P	227.1808942	-1.56677597	0.526369426	-2.976571002	0.002914916
RNU4-2	26.77272092	-1.440710286	0.40570926	-3.551090471	0.000383639
LINC01562	1.667239048	-2.225002449	0.670401345	-3.318911076	0.000903692
BTBD7P1	2.758600783	-1.001663639	0.479901172	-2.087228991	0.03686744
AC083899.1	32.23583231	-1.097137762	0.29780296	-3.684106302	0.000229506
FAM83A-AS1	1.458260412	-1.819352281	0.712658268	-2.552909807	0.010682716
CCL4L1	86.65894202	-1.554705134	0.768384386	-2.023342953	0.043037801
CCL3L1	868.0676435	-1.781708886	0.443660709	-4.015926702	5.92E-05
HBA1	755.2778107	-1.20038963	0.554196065	-2.16600172	0.030311056
HBM	38.89771097	-1.852935629	0.808238705	-2.29255988	0.021873356
RNU1-1	11.47060706	-1.309501012	0.449159046	-2.915450603	0.003551753
RNVU1-15	74.42873682	-2.076629352	0.63085228	-3.291783858	0.000995541
RNU1-4	4.251103289	-1.941711853	0.778941736	-2.492756213	0.012675584
MIR150	2.411175715	-2.002421846	0.784042487	-2.55397109	0.01065021
RNVU1-13	114.088673	-2.013304065	0.570609916	-3.528336973	0.000418179
AL713999.1	2.751389794	-1.25056221	0.501055376	-2.495856288	0.01256535
AL139100.1	3.066767425	-1.165650674	0.509152063	-2.289395955	0.022056357
RNU2-63P	6.768255933	-1.045808455	0.522409083	-2.001895619	0.045295959
FTH1P10	1.650575648	-1.451710298	0.735995184	-1.972445377	0.048558786
AC005165.1	1.288217301	-1.585741882	0.756580177	-2.095933689	0.036088074
HBD	33.75809016	-1.490970966	0.669260748	-2.227787855	0.025894665
AC023157.1	2.772543753	-1.397406665	0.627271348	-2.227754655	0.02589688
SNRPGP15	5.196156826	-1.161709551	0.441493676	-2.631316403	0.008505481
AC004552.1	2.813413036	-1.380343443	0.608912392	-2.266899904	0.023396341
MTND2P28	30.41013195	-1.433523774	0.493467158	-2.905003404	0.003672491
AC005099.1	1.356769253	-2.058536468	0.83454559	-2.466655499	0.01363815
AC021028.1	6.853404508	-1.38393321	0.514667163	-2.688986803	0.007166925
RPS15AP30	4.17582902	-1.292169528	0.467166062	-2.76597474	0.005675293
AL731568.1	1.70828202	-1.647556834	0.798172289	-2.064161906	0.039002368
LINC02585	1.203222616	-1.770438711	0.884508586	-2.001607151	0.045326999
GRK5-IT1	5.140915462	-1.117016825	0.444688593	-2.51190798	0.01200804
EEF1A1P11	33.91404894	-1.190632179	0.402937872	-2.954877816	0.003127929
AC233280.1	1.166692131	-2.278029006	0.865372716	-2.632425269	0.008477767

ORM1	11.56688985	-1.102428089	0.512907452	-2.149370387	0.031605051
CT45A11P	1.330543223	-1.778300017	0.795633485	-2.235074378	0.025412466
AL136320.1	1.646924798	-1.676515966	0.76144617	-2.201752444	0.0276828
EMC1-AS1	3.97601341	-1.082013022	0.550483872	-1.965567162	0.04934863
LIMD1-AS1	14.63019879	-1.116890531	0.378386235	-2.951720832	0.003160085
AC241584.1	7.978756455	-1.110004549	0.527694117	-2.103499951	0.03542209
AL590133.1	3.487256202	-1.004911836	0.508336874	-1.976861975	0.048057234
CFAP58-DT	7.162139696	-1.567916889	0.524215342	-2.990978637	0.002780849
AL592284.1	15.58882875	-1.162904288	0.57148619	-2.034877324	0.041863218
AC007285.2	1.397111774	-1.441789845	0.722693069	-1.995023763	0.046040288
LINC01031	1.606072784	-1.674553141	0.74291321	-2.254036027	0.024193902
SDCBPP3	0.820945931	-2.195981133	0.907776711	-2.419076306	0.015559976
HNRNPLP1	1.136784049	-1.713188544	0.832347397	-2.058261432	0.039565044
RPS20P10	1.274792983	-2.169349337	0.804980621	-2.694908773	0.00704079
AC073529.1	5.000730241	-1.284740629	0.444960264	-2.887315412	0.003885445
ELOBP1	1.193703484	-1.727339864	0.869334037	-1.986969093	0.046925822
LINC01952	2.211539847	-1.815129556	0.685197767	-2.649059358	0.008071615
AL592546.1	7.741343393	-1.093224528	0.383765113	-2.848681373	0.004390082
AL357497.1	1.314516764	-1.942955713	0.90547528	-2.145785486	0.031890097
AL445231.1	6.166897133	-1.271542224	0.388966297	-3.26902931	0.001079171
DAPK1-IT1	1.613898185	-1.697002865	0.725423789	-2.339326187	0.019318558
SNX25P1	2.451227811	-1.358776005	0.601269905	-2.259843698	0.023830953
AL365255.1	3.128576233	-1.352306939	0.6713057	-2.014442807	0.043963064
LINC01115	7.95445401	-1.906738442	0.581670928	-3.278036345	0.001045319
TMED10P2	1.201132643	-1.814117609	0.793989246	-2.284813828	0.022323747
DEFA3	97.51831193	-2.023409194	0.633509464	-3.193968375	0.001403315
AL133163.1	14.65122608	-1.87945327	0.579666217	-3.24230258	0.001185681
AC093627.3	4.994327548	-1.202069876	0.547943666	-2.193783687	0.028250964
AC093583.1	5.105284644	-1.134451427	0.536826268	-2.113256178	0.034578847
RPS4XP14	1.697533979	-1.359284837	0.68588649	-1.981792699	0.047502447
RPL12P6	1.658813269	-1.61279594	0.77546649	-2.079775157	0.037546161
LINC02086	22.79690556	-1.064242596	0.351050004	-3.031598299	0.002432627
LINC01093	3.28669759	-1.918586635	0.716513494	-2.677669927	0.007413624
MIR3945HG	5.237760346	-1.454496271	0.391892784	-3.711464793	0.000206063
TRBV7-7	7.095827095	-1.186899098	0.481371602	-2.46566082	0.013676077
AC023790.2	1.407763232	-2.211855582	0.898202264	-2.462536191	0.013795825
LINC00346	4.520311864	-1.267914803	0.435027015	-2.914565672	0.003561839
YWHABP2	1.305364429	-2.435399462	0.881355377	-2.763243438	0.005723006
CCL3L3	90.25424272	-1.30977238	0.476663596	-2.747791923	0.005999807
TRDV3	7.05447204	-1.092767747	0.508429621	-2.149299926	0.031610632
AP003721.3	3.274731083	-1.172275309	0.588226242	-1.992898692	0.046272542
AC026369.3	19.95558503	-1.001919232	0.424877772	-2.358135206	0.018367002
AC025031.2	3.401023656	-1.18140143	0.486582611	-2.427956537	0.015184163
AC010173.1	1.992244253	-1.464918334	0.58193648	-2.517316553	0.011825253
AL160191.1	0.991696574	-1.730386777	0.879851177	-1.966681211	0.049219974
RBM8B	3.132275588	-1.103686712	0.468929325	-2.353631246	0.018591044
AL157871.2	3.111342476	-1.38038316	0.571175019	-2.416742878	0.015660075
LINC01397	4.178818567	-1.270210545	0.442957694	-2.867566278	0.004136422
AC243965.1	4.38777072	-2.021898737	0.764992383	-2.643031202	0.008216746
AC025580.1	1.935428921	-2.065374226	0.739018268	-2.794753954	0.005193923
AC051619.8	5.490288569	-1.577487521	0.646673158	-2.439389206	0.014712113
AC007952.4	68.29610206	-1.316706309	0.569346148	-2.312663944	0.020741123
LINC01977	2.067022403	-1.448334977	0.715194524	-2.025092375	0.04285788
ABHD15-AS1	2.391623771	-1.993453966	0.830380321	-2.400651744	0.016365904
SNORD3C	10.66184909	-2.211221268	0.843987977	-2.619967734	0.008793809
SNORD3B-1	181.2643352	-1.026461965	0.510889771	-2.009165231	0.044519614
AC024267.4	1.106675991	-1.639772973	0.824724344	-1.988267939	0.046782065
MIR4740	2.836700438	-1.310393462	0.57878754	-2.264031916	0.023572152

AC023421.2	1.194782593	-2.149714907	0.889117738	-2.417806794	0.015614365
AC008752.2	2.370808676	-1.306890149	0.586537062	-2.228145898	0.025870787
KCNJ2-AS1	11.61488858	-1.22345155	0.511076195	-2.39387309	0.016671516
AC104365.2	1.475682333	-1.727508253	0.805857852	-2.143688553	0.03205785
SMIM17	3.046053709	-1.355766061	0.551422917	-2.458668328	0.013945339
ARL14EPL	2.74289163	-1.845916554	0.796734682	-2.316852267	0.020511781
MTDHP3	8.633771035	-1.654260239	0.557614102	-2.96667576	0.003010382
AC011466.3	2.35652536	-1.387905607	0.64611681	-2.148072277	0.031708014
AL360012.1	44.08768656	-1.143623393	0.467727993	-2.445060827	0.014482771
AC018695.3	1.495474797	-1.652093645	0.813252859	-2.031463678	0.042207981
AC104695.3	6.183520826	-1.180283679	0.485789529	-2.429619431	0.015114684
AC013731.1	1.784122912	-1.428913571	0.710642325	-2.010735248	0.044353429
AL024507.2	3.83039118	-1.303043998	0.562662802	-2.315852395	0.020566329
AC009974.1	4.853594781	-1.045603784	0.469286966	-2.228069092	0.025875908
DOC2B	1.712296426	-1.895449412	0.832452268	-2.276946661	0.022789411
AC103702.2	9.255770567	-1.840068746	0.437375908	-4.207064708	2.59E-05
KB-226F1.1	2.257002614	-2.655758538	0.77670195	-3.41927626	0.000627879
LYPD4	2.593204804	-1.361184827	0.647730862	-2.101466685	0.03560002
H2AFB1	1.641349049	-2.70891075	0.855299278	-3.16720804	0.001539102
AP003419.2	1.841981355	-1.27521537	0.6383122	-1.997792569	0.045739153
AC090970.2	1.267712191	-1.684227746	0.799534586	-2.106510181	0.03516006
AC025031.3	7.028936183	-1.593814562	0.418477358	-3.808604058	0.000139754
HIST1H2AH	10.96493851	-1.173225165	0.533399019	-2.199526288	0.027840522
AC245014.3	60.30375074	-2.170411901	0.638933045	-3.396931681	0.00068146
HIST1H2BB	4.947143778	-1.311765817	0.594849817	-2.205205042	0.02743971
HIST1H4D	6.972393349	-1.202925734	0.41677827	-2.886248686	0.00389864
AC004477.3	1.105871261	-2.183260643	0.858842346	-2.542097107	0.011018955
AL031009.1	1.766533151	-1.758856661	0.721345512	-2.438299861	0.014756528
AC109326.1	1060.657398	-1.227252194	0.501335901	-2.447963916	0.014366604
AP006621.5	8.209946181	-1.252227654	0.520645343	-2.405145213	0.016166041
AJ239328.1	1.324920557	-1.793235194	0.867766223	-2.066495729	0.038781695
AL136985.3	3.428688204	-1.47800141	0.660664772	-2.237142758	0.025277011
AL117339.5	2.314662877	-1.358954097	0.575256688	-2.362343844	0.01815979
AC092652.3	9.605803293	-1.242239294	0.32271596	-3.849327115	0.000118443
AL031432.5	7.037903322	-1.332058014	0.518735409	-2.567894904	0.010231817
AC083906.5	3.533135004	-1.336024272	0.601063799	-2.222766158	0.026231572
AL136441.1	2.392750179	-1.881750597	0.684364431	-2.749632377	0.005966216
AL021155.2	179.5739318	-1.063417097	0.416800773	-2.551379859	0.010729731
RNVU1-8	2.775520398	-1.251885064	0.531246118	-2.356506754	0.018447732
AC084871.3	7.337439193	-1.676133684	0.599564473	-2.795585394	0.005180581
HIST1H3C	8.6594466	-1.195461116	0.413421628	-2.891626938	0.003832527
AC132938.6	2.641173563	-1.306968192	0.535626482	-2.440073888	0.014684258
AC069368.2	1.277515446	-1.902883066	0.809982999	-2.349287661	0.018809369
AC061975.8	2.444674915	-1.156588222	0.564379559	-2.049309197	0.04043189
AC092651.3	4.273954411	-1.200964595	0.538660401	-2.229539413	0.025778036

Supplementary Table 6. GO terms enriched in serum of subjects with high vs without anti-Spike RBD antibodies.

NAME	SIZE	ES	NES	NOM p-val	FDR q-val
GOBP_B_CELL_RECEPTOR_SIGNALING_PATHWAY	106	0.556112	2.549422	0	0
GOCC_IMMUNOGLOBULIN_COMPLEX	120	0.507507	2.356203	0	0.0015792
GOMF_IMMUNOGLOBULIN_RECEPTOR_BINDING	55	0.547553	2.204594	0	0.0069527
GOCC_IMMUNOGLOBULIN_COMPLEX_CIRCULATING	53	0.555135	2.191559	0	0.0067924
GOBP_REGULATION_OF_CHROMATIN_ASSEMBLY_OR_DISASSEMBLY	21	0.667285	2.169967	0	0.0068856
GOBP_REGULATION_OF_CHROMATIN_ASSEMBLY	16	0.715119	2.163087	0	0.008177
GOMF_BETA_CATENIN_BINDING	74	0.499747	2.153624	0	0.0079033
GOBP_REGULATION_OF_B_CELL_RECEPTOR_SIGNALING_PATHWAY	25	0.617817	2.079691	0	0.0247169
GOBP_INNERVATION	17	0.659871	2.004098	0	0.0568569
GOCC_SWI_SNF_COMPLEX	18	0.636798	1.955688	0	0.0953557
GOBP_PHAGOCYTOSIS_RECOGNITION	64	0.474807	1.946085	0	0.0989704
GOMF_DNA_BINDING_TRANSCRIPTION_REPRESSOR_ACTIVITY	242	0.373359	1.936321	0	0.1022317
GOMF_HISTONE_BINDING	218	0.375871	1.90461	0	0.1381874
GOBP_HUMORAL_IMMUNE_RESPONSE_MEDIATED_BY_CIRCULATING_IMMUNOGLOBULIN	120	0.403511	1.897032	0	0.1305079
GOCC_DNA_PACKAGING_COMPLEX	19	0.603096	1.88364	0	0.140076
GOBP_NCRNA_EXPORT_FROM_NUCLEUS	37	0.498292	1.854992	0	0.17143
GOBP_SOMITE_DEVELOPMENT	54	0.460841	1.842365	0	0.1848537
GOBP_HISTONE_H3_K9_METHYLATION	31	0.503301	1.81528	0	0.2217373
GOMF_NUCLEOCYTOPLASMIC_CARRIER_ACTIVITY	29	0.522154	1.814727	0	0.2126528
GOBP_B_CELL_ACTIVATION	268	0.34634	1.798492	0	0.2200173
GOCC_PERIKARYON	114	0.387914	1.788232	0	0.2333203
GOMF_TRANSMEMBRANE_RECEPTOR_PROTEIN_TYROSINE_KINASE_ACTIVITY	45	0.461667	1.784098	0	0.226506
GOMF_PROTEIN_TYROSINE_KINASE_ACTIVITY	115	0.381079	1.754506	0	0.2493285
GOMF_GUANYL_NUCLEOTIDE_EXCHANGE_FACTOR_ACTIVITY	185	0.348876	1.734888	0	0.2483897
GOBP_FC_RECEPTOR_MEDIATED_STIMULATORY_SIGNALING_PATHWAY	135	0.361479	1.729142	0	0.2493287
GOMF_GTPASE_ACTIVATOR_ACTIVITY	241	0.336003	1.727262	0	0.2480296
GOBP_MITOCHONDRION_DISTRIBUTION	16	0.631253	1.873925	0.001862197	0.1471817
GOBP_BETA_CATENIN_TCF_COMPLEX_ASSEMBLY	29	0.546157	1.904474	0.001949318	0.1284626
GOMF_OXIDOREDUCTASE_ACTIVITY_ACTING_ON_THE_ALDEHYDE_OR_OXO_GROUP_OF_DONORS_NAD_OR_NADP_AS_ACCEPTOR	27	0.520345	1.768628	0.001968504	0.2367116
GOBP_ACETYL_COA_BIOSYNTHETIC_PROCESS	21	0.549729	1.754163	0.001996008	0.2427389
GOBP_CHROMOSOME_ORGANIZATION_INVOLVED_IN_MEIOTIC_CELL_CYCLE	50	0.438898	1.734127	0.00203252	0.2441325
GOBP_ANTERIOR_POSTERIOR_AXIS_SPECIFICATION	27	0.531449	1.808943	0.00204499	0.2059881
GOBP_BETA_CATENIN_DESTRUCTION_COMPLEX_DISASSEMBLY	19	0.593878	1.826081	0.003921569	0.2083259
GOBP_GLOMERULUS_DEVELOPMENT	45	0.451462	1.752147	0.003921569	0.2406804
GOBP_SOMITOGENESIS	42	0.484333	1.813702	0.004040404	0.205578
GOCC_MICROTUBULE_END	29	0.510831	1.745541	0.005928854	0.243038
GOMF_NUCLEAR_IMPORT_SIGNAL_RECEPTOR_ACTIVITY	19	0.555733	1.763273	0.006060606	0.2423872
GOMF_OXIDOREDUCTASE_ACTIVITY_ACTING_ON_THE_ALDEHYDE_OR_OXO_GROUP_OF_DONORS	32	0.491224	1.778963	0.010121457	0.2301948
GOBP_REGULATION_OF_TRANSCRIPTION_INVOLVED_IN_G1_S_TRANSITION_OF_MITOTIC_CELL_CYCLE	34	0.489497	1.774748	0.011627907	0.2311509
GOBP_V_D_J_RECOMBINATION	15	0.599447	1.784932	0.012396694	0.2326951
GOBP_DETERMINATION_OF_ADULT_LIFESPAN	15	0.609779	1.748409	0.013944224	0.2425209

Supplementary Table 7. GO terms enriched in serum of subjects without vs with high anti-Spike RBD antibodies.

NAME	SIZE	ES	NES	NOM p-val	FDR q-val
GOCC_CYTOSOLIC_RIBOSOME	101	-0.60821	-2.77163	0	0
GOCC_LARGE_RIBOSOMAL_SUBUNIT	111	-0.57812	-2.70526	0	0
GOCC_CYTOSOLIC_LARGE_RIBOSOMAL_SUBUNIT	54	-0.66274	-2.66178	0	0
GOCC_RIBOSOMAL_SUBUNIT	181	-0.53181	-2.64919	0	0
GOMF_STRUCTURAL_CONSTITUENT_OF_RIBOSOME	156	-0.53584	-2.6151	0	0
GOBP_COTRANSLATIONAL_PROTEIN_TARGETING_TO_MEMBRANE	101	-0.57574	-2.60776	0	0
GOBP_ESTABLISHMENT_OF_PROTEIN_LOCALIZATION_TO_ENDOPLASMIC_RETICULUM	114	-0.54875	-2.54147	0	0
GOCC_RIBOSOME	215	-0.48975	-2.47401	0	0
GOBP_PROTEIN_LOCALIZATION_TO_ENDOPLASMIC_RETICULUM	137	-0.51275	-2.41787	0	0
GOBP_NUCLEAR_TRANSCRIBED_MRNA_CATABOLIC_PROCESS_NONSENSE_MEDIATED_DECAY	119	-0.49242	-2.29766	0	3.89E-04
GOBP_PROTEIN_TARGETING_TO_MEMBRANE	192	-0.44781	-2.24365	0	9.65E-04
GOBP_INTERLEUKIN_1_MEDIATED_SIGNALING_PATHWAY	99	-0.48885	-2.20624	0	0.001601982
GOCC_RESPIRASOME	97	-0.48778	-2.19371	0	0.001919336
GOCC_SMALL_NUCLEAR_RIBONUCLEOPROTEIN_COMPLEX	78	-0.50048	-2.18829	0	0.001919324
GOBP_OXIDATIVE_PHOSPHORYLATION	139	-0.45563	-2.17386	0	0.002560743
GOMF_CHEMOKINE_ACTIVITY	31	-0.62231	-2.17132	0	0.002400696
GOBP_REGULATION_OF_CELLULAR_AMINE_METABOLIC_PROCESS	68	-0.50349	-2.15732	0	0.003110109
GOBP_MONOCYTE_CHEMOTAXIS	45	-0.54556	-2.14586	0	0.003150342
GOMF_HEME_COPPER_TERMINAL_OXIDASE_ACTIVITY	23	-0.64976	-2.14231	0	0.003135335
GOCC_PEPTIDASE_COMPLEX	87	-0.48122	-2.14008	0	0.003026669
GOCC_SM_LIKE_PROTEIN_FAMILY_COMPLEX	90	-0.47894	-2.1369	0	0.003019549
GOCC_SPLICEOSOMAL_TRI_SNRNP_COMPLEX	38	-0.57939	-2.1311	0	0.003230531
GOBP_RESPONSE_TO_INTERLEUKIN_1	173	-0.42922	-2.12452	0	0.003465906
GOBP_ANAPHASE_PROMOTING_COMPLEX_DEPENDENT_CATABOLIC_PROCESS	79	-0.48317	-2.1227	0	0.003521086
GOBP_T_HELPER_2_CELL_DIFFERENTIATION	15	-0.73074	-2.12153	0	0.003380243
GOBP_TRANSLATIONAL_INITIATION	185	-0.42601	-2.1148	0	0.003546068
GOCC_RESPIRATORY_CHAIN_COMPLEX	82	-0.48361	-2.10611	0	0.003950617
GOCC_CYTOSOLIC_SMALL_RIBOSOMAL_SUBUNIT	44	-0.55192	-2.10232	0	0.004254356
GOCC_INNER_MITOCHONDRIAL_MEMBRANE_PROTEIN_COMPLEX	137	-0.44544	-2.09983	0	0.004141024
GOBP_ATP_SYNTHESIS_COUPLED_ELECTRON_TRANSPORT	96	-0.46324	-2.08975	0	0.004703062
GOBP_REGULATION_OF_CELLULAR_AMINO_ACID_METABOLIC_PROCESS	56	-0.51978	-2.08949	0	0.004582047
GOMF_ELECTRON_TRANSFER_ACTIVITY	124	-0.44754	-2.08834	0	0.004619998
GOBP_MOTILE_CILIUM_ASSEMBLY	19	-0.6631	-2.08192	0	0.004971257
GOCC_U1_SNRNP	24	-0.63952	-2.0815	0	0.004825043
GOMF_CCR_CHEMOKINE_RECEPTOR_BINDING	20	-0.65277	-2.07909	0	0.004909138
GOCC_MITOCHONDRIAL_LARGE_RIBOSOMAL_SUBUNIT	56	-0.50878	-2.0694	0	0.005412247
GOBP_SPLICEOSOMAL_TRI_SNRNP_COMPLEX_ASSEMBLY	19	-0.66186	-2.06425	0	0.005733191
GOCC_U5_SNRNP	22	-0.64225	-2.0482	0	0.007047067
GOCC_MITOCHONDRIAL_PROTEIN_CONTAINING_COMPLEX	256	-0.38741	-2.03573	0	0.008342746
GOCC_RESPIRATORY_CHAIN_COMPLEX_IV	18	-0.64715	-2.02618	0	0.009548894
GOMF_CHEMOKINE_RECEPTOR_BINDING	36	-0.54884	-2.02295	0	0.009715112
GOBP_ANTIMICROBIAL_HUMORAL_IMMUNE_RESPONSE_MEDIATED_BY_ANTIMICROBIAL_PEPTIDE	34	-0.55872	-2.01607	0	0.010372796
GOCC_SMALL_RIBOSOMAL_SUBUNIT	72	-0.48075	-2.0158	0	0.010199016
GOBP_CD4_POSITIVE_ALPHA_BETA_T_CELL_CYTOKINE_PRODUCTION	16	-0.66446	-2.01259	0	0.010316549
GOCC_PROTEASOME_CORE_COMPLEX	19	-0.66474	-2.00891	0.00204918	0.010747463
GOBP_PROTEASOMAL_UBIQUITIN_INDEPENDENT_PROTEIN_CATABOLIC_PROCESS	21	-0.62337	-1.99811	0.002074689	0.012019446
GOBP_NADH_DEHYDROGENASE_COMPLEX_ASSEMBLY	65	-0.4831	-1.99674	0	0.012009756
GOBP_VIRAL_GENE_EXPRESSION	193	-0.39809	-1.99478	0	0.011939268
GOBP_CYTOPLASMIC_TRANSLATION	97	-0.4385	-1.98983	0	0.012578552
GOCC_ENDOPEPTIDASE_COMPLEX	67	-0.47473	-1.97748	0	0.014173212
GOBP_MITOCHONDRIAL_ELECTRON_TRANSPORT_NADH_TO_UBIQUINONE	53	-0.49549	-1.97463	0	0.01444191
GOBP_ELECTRON_TRANSPORT_CHAIN	163	-0.40462	-1.97404	0	0.014292864
GOCC_ORGANELLAR_RIBOSOME	86	-0.44725	-1.96695	0	0.0153104
GOBP_LIPOPOLYSACCHARIDE_MEDIATED_SIGNALING_PATHWAY	54	-0.49487	-1.96668	0	0.015026875
GOBP_POSITIVE_REGULATION_OF_T_HELPER_CELL_DIFFERENTIATION	20	-0.60918	-1.95348	0	0.017178603
GOCC_T_CELL_RECEPTOR_COMPLEX	118	-0.42089	-1.95323	0	0.016905883
GOBP_POSITIVE_REGULATION_OF_TYPE_2_IMMUNE_RESPONSE	15	-0.66366	-1.94827	0.00204499	0.017536972
GOBP_RESPIRATORY_ELECTRON_TRANSPORT_CHAIN	113	-0.41888	-1.94568	0	0.017766949
GOBP_ANTIGEN_PROCESSING_AND_PRESENTATION_OF_EXOGENOUS_PEPTIDE_ANTIGEN_VIA_MHC_CLASS_I	78	-0.45582	-1.94333	0	0.018002933
GOCC_CYTOCHROME_COMPLEX	31	-0.5426	-1.93639	0	0.018964684
GOCC_NADH_DEHYDROGENASE_COMPLEX	49	-0.49061	-1.93397	0	0.019235557
GOCC_PROTON_TRANSPORTING_ATP_SYNTHASE_COMPLEX	22	-0.5901	-1.93193	0	0.019421896
GOBP_TYPE_2_IMMUNE_RESPONSE	33	-0.52507	-1.92269	0	0.02118371
GOBP_TRANSLATIONAL_ELONGATION	128	-0.40272	-1.92015	0	0.021573424
GOBP_RESPONSE_TO_CHEMOKINE	77	-0.44334	-1.91326	0	0.02282372
GOBP_REGULATION_OF_TRANSCRIPTION_FROM_RNA_POLYMERASE_II_PROMOTER_IN_RESPONSE_TO_HYPOXIA	74	-0.44111	-1.91097	0	0.022985922
GOBP_NIK_NF_KAPPAB_SIGNALING	158	-0.38546	-1.90321	0	0.024778998
GOMF_CYTOKINE_ACTIVITY	140	-0.40158	-1.89857	0	0.025570814
GOBP_TUMOR_NECROSIS_FACTOR_MEDIATED_SIGNALING_PATHWAY	153	-0.39205	-1.88654	0	0.028609287
GOMF_NAD_P_H_DEHYDROGENASE_QUINONE_ACTIVITY	47	-0.48462	-1.86929	0	0.03462808
GOBP_REGULATION_OF_HEMATOPOIETIC_STEM_CELL_DIFFERENTIATION	72	-0.44414	-1.86674	0.00203252	0.035126276
GOBP_SPLICEOSOMAL_SNRNP_ASSEMBLY	46	-0.48353	-1.8496	0	0.041962475
GOBP_POSITIVE_REGULATION_OF_T_CELL_CYTOKINE_PRODUCTION	20	-0.58184	-1.83725	0.004175365	0.047070894
GOCC_PROTEASOME_ACCESSORY_COMPLEX	24	-0.56006	-1.83448	0.001960784	0.04762831
GOBP_MITOCHONDRIAL_TRANSLATIONAL_TERMINATION	88	-0.41893	-1.82987	0	0.049269445
GOBP_ESTABLISHMENT_OF_PROTEIN_LOCALIZATION_TO_MEMBRANE	326	-0.34076	-1.82756	0	0.049902353
GOBP_RESPONSE_TO_TUMOR_NECROSIS_FACTOR	263	-0.35044	-1.82629	0	0.049965747
GOBP_NUCLEAR_TRANSCRIBED_MRNA_CATABOLIC_PROCESS	204	-0.3662	-1.82487	0	0.049917646
GOBP_DEFENSE_RESPONSE_TO_FUNGUS	19	-0.57842	-1.82297	0.00610998	0.05024518
GOCC_TRANSLATION_PREINITIATION_COMPLEX	18	-0.57906	-1.81444	0.010526316	0.054242764
GOBP_ANTIMICROBIAL_HUMORAL_RESPONSE	70	-0.429	-1.81358	0	0.054106247

GOBP_MITOCHONDRIAL_ATP_SYNTHESIS_COUPLED_PROTON_TRANSPORT	21	-0.54854	-1.8054	0.002040816	0.058155477
GOBP_SCF_DEPENDENT_PROTEASOMAL_UBIQUITIN_DEPENDENT_PROTEIN_CATABOLIC_PROCESS	89	-0.4032	-1.79412	0	0.06366705
GOMF_OXIDOREDUCTASE_ACTIVITY_ACTING_ON_NAD_P_H_QUINONE_OR_SIMILAR_COMPOUND_AS_ACCEPTOR	56	-0.43954	-1.79024	0	0.065559484
GOCC_POLYSOMAL_RIBOSOME	30	-0.50717	-1.78984	0.002028398	0.065046534
GOBP_TRANSLATIONAL_TERMINATION	103	-0.39135	-1.78869	0	0.06504985
GOMF_RRNA_BINDING	63	-0.43295	-1.78591	0.002016129	0.06602265
GOBP_LYMPHOCYTE_CHEMOTAXIS	42	-0.47479	-1.7855	0	0.06551364
GOMF_UBIQUITIN_LIKE_PROTEIN_CONJUGATING_ENZYME_ACTIVITY	35	-0.4729	-1.76758	0	0.0769669
GOBP_MRNA_CIS_SPLICING_VIA_SPLICEOSOME	27	-0.52135	-1.75745	0.004166667	0.08368339
GOBP_DETOXIFICATION	110	-0.37776	-1.74518	0	0.092351995
GOBP_ORGAN_OR_TISSUE_SPECIFIC_IMMUNE_RESPONSE	22	-0.53041	-1.74194	0.007766991	0.09416251
GOBP_POSITIVE_REGULATION_OF_CD4_POSITIVE_ALPHA_BETA_T_CELL_DIFFERENTIATION	30	-0.49698	-1.73785	0.003773585	0.09701735
GOBP_POSITIVE_REGULATION_OF_MONONUCLEAR_CELL_MIGRATION	51	-0.43022	-1.73721	0.001872659	0.09661819
GOBP_REGULATION_OF_TYPE_2_IMMUNE_RESPONSE	27	-0.50299	-1.73586	0.00617284	0.09681433
GOBP_RIBONUCLEOPROTEIN_COMPLEX_SUBUNIT_ORGANIZATION	201	-0.34845	-1.73369	0	0.09768229
GOCC_PRECATALYTIC_SPLICEOSOME	53	-0.43356	-1.73256	0	0.09773159
GOCC_U2_TYPE_SPLICEOSOMAL_COMPLEX	92	-0.3865	-1.7305	0	0.0987032
GOCC_EUKARYOTIC_TRANSLATION_INITIATION_FACTOR_3_COMPLEX	17	-0.56814	-1.72731	0.007952286	0.10035333
GOBP_CELLULAR_RESPONSE_TO_MOLECULE_OF_BACTERIAL_ORIGIN	173	-0.35076	-1.72643	0	0.10015541
GOMF_PHOSPHOLIPASE_A2_ACTIVITY	21	-0.54701	-1.72082	0.004032258	0.104300976
GOBP_REGULATION_OF_HEMATOPOIETIC_PROGENITOR_CELL_DIFFERENTIATION	85	-0.3873	-1.72044	0	0.10362575
GOBP_POSITIVE_REGULATION_OF_INTERFERON_GAMMA_PRODUCTION	58	-0.4215	-1.71573	0.002	0.10686686
GOBP_REGULATION_OF_T_HELPER_CELL_DIFFERENTIATION	36	-0.46285	-1.71248	0	0.10867202
GOBP_NEGATIVE_REGULATION_OF_CELL_CYCLE_G2_M_PHASE_TRANSITION	104	-0.3806	-1.71216	0.00203252	0.10796638
GOBP_GRANULOCYTE_CHEMOTAXIS	100	-0.37541	-1.70796	0.001996008	0.110934325
GOBP_RESPONSE_TO_INTERFERON_GAMMA	163	-0.34858	-1.7036	0	0.11451461
GOMF_CYTOKINE_RECEPTOR_BINDING	182	-0.34132	-1.69769	0	0.119515136
GOBP_HEMATOPOIETIC_STEM_CELL_DIFFERENTIATION	84	-0.38449	-1.69721	0.001972387	0.11895524
GOBP_NEUTROPHIL_CHEMOTAXIS	85	-0.38097	-1.69513	0.00210084	0.12017503
GOBP_ENDOSOME_TRANSPORT_VIA_MULTIVESICULAR_BODY_SORTING_PATHWAY	30	-0.48054	-1.68799	0.001976285	0.12659109
GOBP_GLYCOSYL_COMPOUND_CATABOLIC_PROCESS	39	-0.44701	-1.68621	0.005813954	0.12729321
GOMF_ANTIOXIDANT_ACTIVITY	72	-0.38718	-1.68124	0.001926782	0.13172401
GOBP_NEGATIVE_REGULATION_OF_SIGNAL_TRANSDUCTION_IN_ABSENCE_OF_LIGAND	24	-0.50212	-1.67827	0.008080808	0.13412052
GOMF_RECEPTOR_INHIBITOR_ACTIVITY	18	-0.56363	-1.67749	0.012605042	0.1338399
GOBP_POSITIVE_REGULATION_OF_T_CELL_MEDIATED_IMMUNITY	47	-0.43131	-1.67543	0.004008016	0.13494267
GOBP_MITOCHONDRIAL_ELECTRON_TRANSPORT_CYTOCHROME_C_TO_OXYGEN	19	-0.53125	-1.67351	0.014227643	0.13593069
GOBP_MITOCHONDRIAL_RESPIRATORY_CHAIN_COMPLEX_ASSEMBLY	100	-0.36914	-1.673	0.002096436	0.1353564
GOMF_SERINE_HYDROLASE_ACTIVITY	117	-0.36124	-1.67064	0.00617284	0.13684668
GOBP_DOPAMINE_METABOLIC_PROCESS	24	-0.50602	-1.66916	0.006012024	0.13756144
GOBP_INNATE_IMMUNE_RESPONSE_ACTIVATING_SIGNAL_TRANSDUCTION	105	-0.36946	-1.66879	0	0.13694075
GOCC_CHAPERONE_COMPLEX	25	-0.50829	-1.66834	0.009615385	0.13632938
GOBP_GRANULOCYTE_MIGRATION	117	-0.3585	-1.66718	0.001851852	0.13637511
GOMF_THREONINE_TYPE_PEPTIDASE_ACTIVITY	18	-0.54403	-1.66664	0.010141988	0.13589455
GOBP_CELLULAR_OXIDANT_DETOXIFICATION	84	-0.38246	-1.66657	0	0.13485296
GOBP_AMINE_METABOLIC_PROCESS	131	-0.34773	-1.6638	0.00212766	0.13719554
GOBP_NEGATIVE_REGULATION_OF_UBIQUITIN_PROTEIN_TRANSFERASE_ACTIVITY	16	-0.567	-1.66353	0.017857144	0.13640246
GOBP_REGULATION_OF_MONOCYTE_CHEMOTAXIS	19	-0.53779	-1.66181	0.01968504	0.13734835
GOBP_RESPONSE_TO_COPPER_ION	31	-0.47185	-1.66085	0.011811024	0.13736014
GOBP_PROTEIN_IMPORT_INTO_MITOCHONDRIAL_MATRIX	20	-0.51073	-1.66026	0.01002004	0.13694614
GOBP_FORMATION_OF_CYTOPLASMIC_TRANSLATION_INITIATION_COMPLEX	16	-0.5595	-1.65919	0.020618556	0.13722843
GOCC_SPLICEOSOMAL_COMPLEX	181	-0.33695	-1.6568	0	0.13872316
GOMF_MHC_CLASS_I_PROTEIN_BINDING	19	-0.53254	-1.65617	0.013779528	0.13850996
GOCC_EUKARYOTIC_48S_PREINITIATION_COMPLEX	15	-0.56032	-1.65572	0.018595042	0.13802838
GOCC_POLYSOME	62	-0.4034	-1.65559	0.001992032	0.13712004
GOCC_INTRINSIC_COMPONENT_OF_MITOCHONDRIAL_OUTER_MEMBRANE	21	-0.52701	-1.65528	0.004192872	0.1364495
GOBP_CELLULAR_RESPONSE_TO_TOXIC_SUBSTANCE	98	-0.36459	-1.65415	0.005813954	0.13668726
GOBP_REGULATION_OF_CD4_POSITIVE_ALPHA_BETA_T_CELL_DIFFERENTIATION	48	-0.42351	-1.65112	0.005836576	0.1392592
GOBP_SLEEP	24	-0.50212	-1.64771	0.024193548	0.1419934
GOMF_MHC_PROTEIN_BINDING	34	-0.4528	-1.6464	0.007905139	0.14268382
GOBP_NEUTROPHIL_MIGRATION	99	-0.36628	-1.64465	0	0.14375576
GOBP_VIRION_ASSEMBLY	40	-0.43992	-1.64171	0.012605042	0.1462512
GOBP_AMYLOID_FIBRIL_FORMATION	21	-0.50801	-1.64074	0.03219697	0.14642288
GOBP_POSITIVE_REGULATION_OF_LEUKOCYTE_ADHESION_TO_VASCULAR_ENDOTHELIAL_CELL	20	-0.51313	-1.63973	0.018218623	0.1465126
GOCC_U12_TYPE_SPLICEOSOMAL_COMPLEX	27	-0.47217	-1.6391	0.008130081	0.1462029
GOBP_CELL_COMMUNICATION_BY_ELECTRICAL_COUPLING_INVOLVED_IN_CARDIAC_CONDUCTION	18	-0.53452	-1.63709	0.028688524	0.14759466
GOBP_CALCIUM_MEDIATED_SIGNALING_USING_INTRACELLULAR_CALCIUM_SOURCE	17	-0.5319	-1.63056	0.024208566	0.15452282
GOBP_MYOBLAST_FUSION	34	-0.45477	-1.6296	0.003853565	0.1546379
GOBP_ENDOCRINE_HORMONE_SECRETION	29	-0.4706	-1.62603	0.007952286	0.158023
GOMF_GDP_BINDING	63	-0.38917	-1.62532	0.00203252	0.1579145
GOBP_T_CELL_CYTOKINE_PRODUCTION	34	-0.45258	-1.62265	0.010060363	0.1604592
GOMF_RIBOSOME_BINDING	54	-0.4073	-1.62157	0.003952569	0.16075261
GOCC_U2_SNRNP	21	-0.51263	-1.61962	0.02636535	0.16221973
GOBP_CELL_COMMUNICATION_BY_ELECTRICAL_COUPLING	23	-0.49246	-1.61942	0.013779528	0.16134043
GOBP_REGULATION_OF_VIRAL_TRANSCRIPTION	40	-0.42939	-1.61454	0.02079002	0.16662726
GOBP_POSITIVE_REGULATION_OF_CD4_POSITIVE_ALPHA_BETA_T_CELL_ACTIVATION	36	-0.44368	-1.6116	0.00996016	0.16959104
GOMF_PRE_MRNA_BINDING	38	-0.43395	-1.60877	0.010460251	0.1721943
GOBP_ANTIGEN_PROCESSING_AND_PRESENTATION_OF_PEPTIDE_ANTIGEN_VIA_MHC_CLASS_I	96	-0.35528	-1.60683	0.007984032	0.17370392
GOMF_SNAP_RECEPTOR_ACTIVITY	33	-0.45284	-1.60386	0.014084507	0.17675386
GOBP_NEGATIVE_REGULATION_OF_PHAGOCYTOSIS	20	-0.50374	-1.60327	0.02385686	0.17643371
GOBP_POSITIVE_REGULATION_OF_REACTIVE_OXYGEN_SPECIES_BIOSYNTHETIC_PROCESS	47	-0.41296	-1.60267	0.01010101	0.17607722
GOBP_EXTRACELLULAR_TRANSPORT	30	-0.44601	-1.60264	0.013565891	0.17503178
GOBP_REGULATION_OF_ATP_BIOSYNTHETIC_PROCESS	19	-0.50702	-1.60104	0.016842104	0.17626132
GOBP_RNA_CATABOLIC_PROCESS	391	-0.29554	-1.60065	0	0.17568374

GOCC_SNARE_COMPLEX	43	-0.42487	-1.60006	0.010845987	0.17528802
GOCC_GLYCOPROTEIN_COMPLEX	15	-0.55911	-1.59238	0.026915114	0.1848911
GOBP_NUCLEOSIDE_CATABOLIC_PROCESS	29	-0.44968	-1.59228	0.019267824	0.18390504
GOBP_INNER_EAR_RECEPTOR_CELL_DEVELOPMENT	30	-0.45164	-1.59007	0.020366598	0.18593597
GOBP_PROTON_TRANSMEMBRANE_TRANSPORT	130	-0.33281	-1.59003	0	0.18488131
GOBP_PROTEIN_TARGETING	409	-0.28876	-1.58746	0	0.1874749
GOBP_CELLULAR_RESPONSE_TO_OXYGEN_RADICAL	17	-0.51752	-1.58636	0.03448276	0.18791413
GOMF_TRANSLATION_ELONGATION_FACTOR_ACTIVITY	18	-0.51814	-1.58408	0.022222223	0.19004194
GOBP_RIBONUCLEOPROTEIN_COMPLEX_BIOGENESIS	439	-0.2869	-1.58359	0	0.18967018
GOBP_REGULATION_OF_VASCULAR_PERMEABILITY	34	-0.43833	-1.58038	0.022540983	0.19344631
GOBP_RIBOSOME_ASSEMBLY	59	-0.38682	-1.57472	0.005882353	0.20089768
GOBP_C21_STEROID_HORMONE_BIOSYNTHETIC_PROCESS	20	-0.49925	-1.57348	0.0332681	0.20172857
GOBP_POSITIVE_REGULATION_OF_CYTOKINE_PRODUCTION_INVOLVED_IN_IMMUNE_RESPONSE	51	-0.3894	-1.57238	0.010060363	0.202436
GOBP_AXONEME_ASSEMBLY	57	-0.38667	-1.5712	0.008281574	0.20301688
GOBP_REGULATION_OF_CARDIAC_CONDUCTION	42	-0.40677	-1.57114	0.013215859	0.20200056
GOBP_RNA_SPLICING_VIA_TRANSESTERIFICATION_REACTIONS	346	-0.29365	-1.57052	0	0.20192873
GOCC_SARCOPLASMIC_RETICULUM_MEMBRANE	28	-0.45755	-1.57047	0.028985508	0.20089824
GOBP_PYRIMIDINE_NUCLEOSIDE_CATABOLIC_PROCESS	19	-0.51067	-1.56983	0.04518664	0.20068611
GOBP_REGULATION_OF_HETEROTYPIC_CELL_CELL_ADHESION	18	-0.49822	-1.56908	0.039748956	0.20065145
GOBP_POSITIVE_REGULATION_OF_ADAPTIVE_IMMUNE_RESPONSE	96	-0.34701	-1.56863	0.006355932	0.2001705
GOMF_OXIDOREDUCTASE_ACTIVITY_ACTING_ON_PEROXIDE_AS_ACCEPTOR	48	-0.40236	-1.56847	0.011881189	0.19937783
GOBP_CYTOPLASMIC_TRANSLATIONAL_INITIATION	34	-0.43767	-1.56843	0.016701462	0.19837281
GOBP_CATECHOL_CONTAINING_COMPOUND_METABOLIC_PROCESS	34	-0.43192	-1.56583	0.0125	0.20110841
GOBP_POSITIVE_REGULATION_OF_RESPONSE_TO_BIOTIC_STIMULUS	218	-0.30608	-1.56512	0	0.20113091
GOBP_LATE_ENDOSOME_TO_VACUOLE_TRANSPORT	24	-0.46664	-1.56244	0.04411765	0.20414092
GOBP_POSITIVE_REGULATION_OF_PRODUCTION_OF_MOLECULAR_MEDIATOR_OF_IMMUNE_RESPONSE	86	-0.35692	-1.56188	0.008264462	0.2038955
GOBP_VIRAL_BUDDING_VIA_HOST_ESCRT_COMPLEX	21	-0.48857	-1.56061	0.03177966	0.20482607
GOBP_POSITIVE_REGULATION_OF_INTRINSIC_APOPTOTIC_SIGNALING_PATHWAY	53	-0.3843	-1.55856	0.012320329	0.20708963
GOBP_POSITIVE_REGULATION_OF_VASCULAR_PERMEABILITY	15	-0.53783	-1.55721	0.028901733	0.2081378
GOMF_LAMININ_BINDING	26	-0.45771	-1.55655	0.029821074	0.20824115
GOBP_NEGATIVE_REGULATION_OF_PEPTIDE_SECRETION	55	-0.38963	-1.55648	0.014403292	0.20736068
GOBP_MYD88_INDEPENDENT_TOLL_LIKE_RECEPTOR_SIGNALING_PATHWAY	33	-0.43263	-1.55639	0.015053763	0.20644943
GOBP_AUDITORY_RECEPTOR_CELL_DEVELOPMENT	16	-0.52429	-1.55619	0.028806584	0.2056495
GOBP_NEGATIVE_REGULATION_OF_PROTEASOMAL_UBIQUITIN_DEPENDENT_PROTEIN_CATABOLIC_PROCESS	31	-0.44282	-1.55288	0.033333335	0.20977862
GOBP_RESPONSE_TO_GONADOTROPIN	20	-0.49374	-1.54708	0.034343433	0.21788692
GOBP_NEGATIVE_REGULATION_OF_SIGNALING_RECEPTOR_ACTIVITY	38	-0.41042	-1.5457	0.018108651	0.2190277
GOBP_AXONEMAL_DYNEIN_COMPLEX_ASSEMBLY	25	-0.45119	-1.54487	0.031620555	0.21934417
GOBP_NEGATIVE_REGULATION_OF_NIK_NF_KAPPAB_SIGNALING	24	-0.47119	-1.54319	0.027888447	0.22104639
GOBP_MITOCHONDRIAL_MEMBRANE_ORGANIZATION	135	-0.32605	-1.5425	0.002070393	0.22107776
GOBP_RESPONSE_TO_MUSCLE_ACTIVITY	18	-0.50865	-1.54168	0.035856575	0.22133493
GOMF_RECEPTOR_REGULATOR_ACTIVITY	299	-0.29139	-1.54135	0	0.22083096
GOBP_T_CELL_DIFFERENTIATION_INVOLVED_IN_IMMUNE_RESPONSE	67	-0.36327	-1.54003	0.010504202	0.22186415
GOBP_REGULATION_OF_DNA_TEMPLATED_TRANSCRIPTION_IN_RESPONSE_TO_STRESS	107	-0.33674	-1.53952	0	0.2216472
GOBP_CELLULAR_RESPONSE_TO_BIOTIC_STIMULUS	196	-0.30884	-1.53935	0	0.22091301
GOBP_SYNAPTIC_TRANSMISSION_CHOLINERGIC	20	-0.48187	-1.53478	0.023206752	0.22751206
GOBP_ACTIVATION_OF_INNATE_IMMUNE_RESPONSE	132	-0.32374	-1.5346	0.009765625	0.22680832
GOCC_SNO_S_RNA_CONTAINING_RIBONUCLEOPROTEIN_COMPLEX	25	-0.44578	-1.53445	0.030737706	0.22601558
GOBP_REGULATION_OF_NIK_NF_KAPPAB_SIGNALING	85	-0.35152	-1.53414	0.00617284	0.22536178
GOBP_RIBONUCLEOSIDE_CATABOLIC_PROCESS	21	-0.48741	-1.53269	0.035196688	0.22565569
GOBP_INTERLEUKIN_8_PRODUCTION	70	-0.3614	-1.53256	0.007874016	0.22478053
GOMF_TRANSLATION_FACTOR_ACTIVITY_RNA_BINDING	80	-0.34803	-1.52771	0.007984032	0.23197137
GOBP_NEGATIVE_REGULATION_OF_PEPTIDYL_SERINE_PHOSPHORYLATION	25	-0.45714	-1.52752	0.046121594	0.23123413
GOBP_OUTER_DYNEIN_ARM_ASSEMBLY	15	-0.52046	-1.52663	0.036072146	0.23172782
GOBP_RESPONSE_TO_MOLECULE_OF_BACTERIAL_ORIGIN	277	-0.29009	-1.52626	0	0.23132186
GOBP_NUCLEOTIDE_BINDING_DOMAIN_LEUCINE_RICH_REPEAT_CONTAINING_RECEPTOR_SIGNALING_PATHWAY	39	-0.4082	-1.52425	0.028571429	0.23350045
GOMF_PROTEIN_FOLDING_CHAPERONE	31	-0.42726	-1.52404	0.036885247	0.2327998
GOMF_TRANSLATION_INITIATION_FACTOR_ACTIVITY	49	-0.38273	-1.52365	0.014256619	0.23234308
GOBP_NEGATIVE_REGULATION_OF_EXTRINSIC_APOPTOTIC_SIGNALING_PATHWAY	80	-0.3473	-1.52305	0.010121457	0.23236328
GOBP_CELLULAR_RESPONSE_TO_DSRNA	20	-0.48174	-1.52218	0.04621849	0.23278274
GOBP_REGULATION_OF_STEM_CELL_DIFFERENTIATION	100	-0.33845	-1.52168	0.006060606	0.23257488
GOBP_REGULATION_OF_LEUKOCYTE_ADHESION_TO_VASCULAR_ENDOTHELIAL_CELL	24	-0.46033	-1.52153	0.030042918	0.23176289
GOBP_RESPONSE_TO_FUNGUS	32	-0.42079	-1.51994	0.025540275	0.2331965
GOBP_MONONUCLEAR_CELL_MIGRATION	151	-0.3153	-1.51946	0.001949318	0.23303959
GOCC_PROTON_TRANSPORTING_TWO_SECTOR_ATPASE_COMPLEX	45	-0.3897	-1.51677	0.014198783	0.23671566
GOCC_ESCRT_COMPLEX	27	-0.44449	-1.51588	0.040084388	0.2372717
GOBP_POSITIVE_REGULATION_OF_NATURAL_KILLER_CELL_MEDIATED_CYTOTOXICITY	23	-0.46319	-1.51425	0.030864198	0.23902473
GOBP_REGULATION_OF_MITOCHONDRIAL_MEMBRANE_PERMEABILITY	67	-0.35531	-1.50903	0.021825397	0.24679293
GOBP_REGULATION_OF_MYOBLAST_FUSION	16	-0.50869	-1.50642	0.041237112	0.2490181

Supplementary Table 8. Percentage of T cells,B cells,Monocyte cells obtained by flow cytometric analysis

Subject ID	B cell percent of CD45+	T cell percent of CD45+	Monocyte cell percent of CD45+	B cell per/L of blood (10^9)	T cell per/L of blood (10^9)	Monocye cell per/L of blood (10^9)	Group
P475	2.16	58.3	11.8	0.202	5.469	1.102	Low
P435	2.96	66.7	4.76	0.244	5.514	0.394	Low
P383	4.47	65.5	4.58	0.402	5.903	0.412	Low
P30	1.84	73.5	4.27	0.172	7.008	0.406	Low
P354	4.79	71.8	3.9	0.400	5.997	0.325	Low
P386	4.5	65.7	3.41	0.427	6.186	0.321	Low
P300	4.27	71.6	4.68	0.373	6.247	0.408	Low
P36	6.8	54.1	4.4	0.426	3.374	0.275	Low
P301	12.7	61.5	0.9	1.151	5.588	0.083	Low
P378	2.3	58.8	3	0.189	4.911	0.250	Low
P26	11.9	60.8	3.14	0.537	2.751	0.142	Low
P453	4	62.8	1.1	0.346	5.428	0.097	Low
P22	6.87	59	8.08	0.567	4.864	0.666	High
P12	8.6	61.8	4.95	0.735	5.288	0.423	High
P44	13.2	54.4	3.02	0.976	4.029	0.223	High
P16	9	59.7	0.9	0.735	4.854	0.070	High
P35	6.9	73.5	1.8	0.639	6.804	0.169	High
P17	6.1	58.5	3.3	0.490	4.714	0.267	High
P33	6.6	61	5.3	0.559	5.179	0.448	High
P38	16.5	51	0.96	1.384	4.268	0.080	High
P23	4.78	39.7	5.43	0.422	3.503	0.479	High
P6	7.82	55.8	6.72	0.720	5.135	0.618	High
P5	6.59	50	13.1	0.519	3.935	1.030	High
P14	7.6	35.1	7.1	0.624	2.877	0.579	High
P9	8.9	42.7	5.1	0.702	3.380	0.405	High
P7	11.5	37.1	5.93	1.009	3.261	0.522	High

Supplementary Table 9. Clinical information of 20 COVID-19 patients with DIA mass spectrometry experiment

ID	Antibodies Group	Clinical classification	Gender	Age	AcE Type	AcE enzyme activity	Days Interval between morbidity and sampling	morbidity Day	sampling Day	Antibody Level
cyto-1	High	Severe	male	35	ID	33.5828	9	1.13	1.22	2.1485
cyto-2	High	Moderate	female	35	II	32.7136	3	1.19	1.22	1.6316
cyto-3	High	Moderate	female	67	ID	53.1003	3	1.19	1.22	1.8227
cyto-4	High	Moderate	male	44	ID	30.8171	4	1.18	1.22	1.6193
Cov-1	High	Critically severe	male	39	II	22.9943	11	1.11	1.22	2.0676
Cov-2	High	Critically severe	male	23	DD	35.0051	7	1.15	1.22	2.0970
cyto-8	High	Moderate	female	40	ID	53.1793	8	1.14	1.22	1.7026
cyto-15	High	Severe	male	42	II	45.1194	9	1.16	1.25	1.9868
cyto-17	High	Moderate	male	29	ID	33.5828	9	1.16	1.25	2.2416
Cov-4	High	Critically severe	female	52	DD	24.2586	11	1.14	1.25	2.0529
Cov-5	High	Critically severe	female	31	DD	36.9806	9	1.17	1.26	2.6164
Cov-6	High	Critically severe	male	60	II	19.5965	4	1.22	1.26	3.2656
cyto-22	High	Moderate	male	35	DD	34.0569	9	1.17	1.26	1.5752
cyto-28	High	Severe	male	54	ID	22.3622	11	1.15	1.26	1.9770
cyto-11	Low	Severe	female	54	ID	32.0024	13	1.11	1.24	0.1396
cyto-12	Low	Moderate	male	52	II	37.3756	6	1.19	1.25	0.9407
Cov-3	Low	Critically severe	male	28	DD	47.3320	6	1.19	1.25	0.3650
cyto-18	Low	Moderate	female	63	ID	35.5582	17	1.9	1.26	0.4581
cyto-23	Low	Critically severe	male.	53	DD	36.9015	7	1.19	1.26	0.5414
cyto-25	Low	Moderate	male	46	DD	26.7872	11	1.15	1.26	0.4385

Supplementary Table 10.Up-regulated proteins in the plasma of COVID-19 patients with vs without anti-Spike RBD antibodies detected.

ProteinGroupID	logFC	AveExpr	P.Value	Protein.Group	Protein.Ids	Protein.Names	Genes	First.Protein.Description
314	2.857819715	21.23946069	0.0002388	P0DOX3	P0DOX3;P01880	IGD_HUMAN	0	Immunoglobulin delta heavy chain
141	3.659191268	20.96708837	0.0014833	P01601	P01601	KVD16_HUMAN	IGKV1D-16	Immunoglobulin kappa variable 1D-16
588	2.631536319	17.37176657	0.0028703	Q15113	Q15113	PCOC1_HUMAN	PCOLCE	Procollagen C-endopeptidase enhancer 1
33	1.098807973	23.06774257	0.003316	A0A0C4DH31	A0A0C4DH31	HV118_HUMAN	IGHV1-18	Immunoglobulin heavy variable 1-18
36	2.932639402	17.95395499	0.003316	A0A0C4DH33	A0A0C4DH33	HV124_HUMAN	IGHV1-24	Immunoglobulin heavy variable 1-24
150	1.775092204	21.75263882	0.0046911	P01742	P01742	HV169_HUMAN	IGHV1-69	Immunoglobulin heavy variable 1-69
30	2.823386142	19.71668229	0.0071343	A0A0B4J2H0;P01742	P01742;A0A0B4J2H0	HV169_HUMAN;HV69D_HUMAN	IGHV1-69;IGHV1-69D	Immunoglobulin heavy variable 1-69D
176	1.103464858	29.54344852	0.0076851	P01871;P0DOX6	P01871;P0DOX6	IGHM_HUMAN;IGM_HUMAN	IGHM	Immunoglobulin heavy constant mu
153	0.963403788	22.25795313	0.0160096	P01768;P0DP03	P0DP02;P0DP03;P01768	HV330_HUMAN;HVC05_HUMAN	IGHV3-30;IGHV3-30-5	Immunoglobulin heavy variable 3-30
175	0.902861889	21.92629506	0.0236527	P01871	P01871	IGHM_HUMAN	IGHM	Immunoglobulin heavy constant mu
157	1.234633051	23.61128352	0.025396	P01824	P06331;P0DP06;P01824;P0DP08;A0A	HV439_HUMAN	IGHV4-39	Immunoglobulin heavy variable 4-39
346	1.522213642	15.93882729	0.0287097	P12956	P12956	XRCC6_HUMAN	XRCC6	X-ray repair cross-complementing protein 6
170	0.969612638	25.87129343	0.0306668	P01859;P01861	P01859;P01861	IGHG2_HUMAN;IGHG4_HUMAN	IGHG2;IGHG4	Immunoglobulin heavy constant gamma 2
188	0.95819588	22.31113375	0.0321185	P02654	P02654	APOC1_HUMAN	APOC1	Apolipoprotein C-I
398	2.406289897	21.69801917	0.0324773	P23083	P23083	HV102_HUMAN	IGHV1-2	Immunoglobulin heavy variable 1-2
81	1.237045675	17.92202972	0.0335852	O43866	O43866	CD5L_HUMAN	CD5L	CD5 antigen-like
17	2.41274425	18.62310769	0.0336526	A0A0B4J1U3	A0A0B4J1U3	LV136_HUMAN	IGLV1-36	Immunoglobulin lambda variable 1-36
463	3.230850729	17.70631706	0.0369423	P41218	P41218	MNDA_HUMAN	MNDA	Myeloid cell nuclear differentiation antigen
238	1.297030083	22.96035987	0.0390594	P04430	P04430	KV116_HUMAN	IGKV1-16	Immunoglobulin kappa variable 1-16
201	0.821430737	26.2360016	0.043974	P02749	P02749	APOH_HUMAN	APOH	Beta-2-glycoprotein 1
19	1.379464845	23.4624614	0.0465779	A0A0B4J1V0	A0A0B4J1V0	HV315_HUMAN	IGHV3-15	Immunoglobulin heavy variable 3-15
537	1.672682984	17.43823968	0.0468165	P80188	P80188	NGAL_HUMAN	LCN2	Neutrophil gelatinase-associated lipocalin
37	2.054634503	19.5597032	0.0469303	A0A0C4DH34	A0A0C4DH34	HV428_HUMAN	IGHV4-28	Immunoglobulin heavy variable 4-28
230	2.068952102	21.39080745	0.0498968	P04196	P04196	HRG_HUMAN	HRG	Histidine-rich glycoprotein

Supplementary Table 11. Down-regulated proteins in the plasma of COVID-19 patients with vs without anti-Spike RBD antibodies detected.

ProteinGroupID	logFC	AveExpr	P.Value	Protein.Group	Protein.Ids	Protein.Names	Genes	First.Protein.Description
462	-1.57813495	16.1991539	0.000458258	P40925	P40925	MDHC_HUMAN	MDH1	Malate dehydrogenase, cytoplasmic
496	-2.78167811	16.48223829	0.000833654	P55287	P55287	CAD11_HUMAN	CDH11	Cadherin-11
489	-1.59449695	15.54678609	0.00103601	P53621	P53621	COPA_HUMAN	COPA	Coatomer subunit alpha
241	-1.39885568	15.94786094	0.001727356	P04745;P04746;P19961	P04745;P19961;P04746	AMY1_HUMAN;AMY2B_HUMAN;AMYP_HUMAN	AMY1A;AMY2A;AMY2B	Alpha-amylase 1
356	-1.81526797	16.20351157	0.00208367	P14543	P14543	NID1_HUMAN	NID1	Nidogen-1
308	-1.20610798	23.9444316	0.003325666	P0C0L5	P0C0L5	CO4B_HUMAN	C4B	Complement C4-B
263	-2.27213057	16.05575903	0.005784026	P06732	P06732	KCRM_HUMAN	CKM	Creatine kinase M-type
183	-1.67670868	15.82898118	0.006813886	P02538;P48668	P48668;P02538	K2C6A_HUMAN;K2C6C_HUMAN	KRT6A;KRT6C	Keratin, type II cytoskeletal 6A
90	-2.85423487	16.64291811	0.007282882	O75083	O75083	WDR1_HUMAN	WDR1	WD repeat-containing protein 1
497	-1.51341511	17.10618793	0.008087965	P55290	P55290	CAD13_HUMAN	CDH13	Cadherin-13
580	-1.21051371	15.93510606	0.008144065	Q14563	Q14563	SEM3A_HUMAN	SEMA3A	Semaphorin-3A
378	-1.24808743	15.74575967	0.009258101	P19022	P19022	CADH2_HUMAN	CDH2	Cadherin-2
483	-1.45643279	15.85182769	0.01005026	P51452	P51452	DUS3_HUMAN	DUSP3	Dual specificity protein phosphatase 3
550	-1.12753257	24.42539384	0.012218143	Q06033	Q06033	ITIH3_HUMAN	ITIH3	Inter-alpha-trypsin inhibitor heavy chain H3
385	-1.22082041	15.93261114	0.013697763	P20340;Q9NRW1	P20340;Q9NRW1	RAB6A_HUMAN;RAB6B_HUMAN	RAB6A;RAB6B	Ras-related protein Rab-6A
70	-1.00331643	17.09658725	0.013823715	O14498	O14498	ISLR_HUMAN	ISLR	Immunoglobulin superfamily containing leucine-rich repeat protein
287	-1.42771836	15.9793009	0.018761859	P08195	P08195	4F2_HUMAN	SLC3A2	4F2 cell-surface antigen heavy chain
56	-1.54406768	16.15667758	0.01937811	A6NGU5;B5MD39;P19440;P36268;Q14390	Q14390;A6NGU5;P36268;P19440;B5MD39	GGT1_HUMAN;GGT2_HUMAN;GGT3_HUMAN;GGTL2_HUMAN;GGTL3_HUMAN	GGT1;GGT2;GGT3P;GGTLC2;GGTLC3	Putative glutathione hydrolase 3 proenzyme
540	-1.2390813	15.65012462	0.020606032	P98160	P98160	PGBM_HUMAN	HSPG2	Basement membrane-specific heparan sulfate proteoglycan core protein
412	-1.42433742	16.14778218	0.022424508	P26022	P26022	PTX3_HUMAN	PTX3	Pentraxin-related protein PTX3
652	-1.15606889	16.1686226	0.023307961	Q9BRF8	Q9BRF8	CPPED_HUMAN	CPPED1	Serine/threonine-protein phosphatase CPPED1
62	-0.95295029	15.75711863	0.024418558	O00194	O00194	RB27B_HUMAN	RAB27B	Ras-related protein Rab-27B
459	-1.13351928	15.6461343	0.024569933	P39059	P39059	COFA1_HUMAN	COL15A1	Collagen alpha-1(XV) chain
309	-3.04627419	24.09788113	0.024821707	P0DJI8	P0DJI8	SAA1_HUMAN	SAA1	Serum amyloid A-1 protein
184	-2.40118796	15.7490526	0.025147394	P02549	P02549	SPTA1_HUMAN	SPTA1	Spectrin alpha chain, erythrocytic 1
556	-1.64464899	16.60192182	0.025876263	Q08830	Q08830	FGL1_HUMAN	FGL1	Fibrinogen-like protein 1
671	-1.10295507	16.10228524	0.026817814	Q9NVA2	Q14141;Q9NVA2;Q92599	SEP11_HUMAN	SEPTIN11	Septin-11
358	-1.196802	19.16691538	0.028183952	P14625	P14625	ENPL_HUMAN	HSP90B1	Endoplasmin
473	-1.64012904	16.19985591	0.028345633	P49189	P49189	AL9A1_HUMAN	ALDH9A1	4-trimethylaminobutyraldehyde dehydrogenase
607	-2.19047095	17.31798194	0.028448731	Q6EMK4	Q6EMK4	VASN_HUMAN	VASN	Vasorin
112	-1.40671238	22.49999486	0.030259274	P00736	P00736	C1R_HUMAN	C1R	Complement C1r subcomponent
121	-0.76342036	27.96374277	0.031240844	P00751	P00751	CFAB_HUMAN	CFB	Complement factor B
466	-1.15883918	15.90241498	0.032395699	P43121	P43121	MUC18_HUMAN	MCAM	Cell surface glycoprotein MUC18
87	-1.62856015	15.97507297	0.036397226	O60610	O60610	DIAP1_HUMAN	DIAPH1	Protein diaphanous homolog 1
290	-1.41151866	15.75429931	0.036502037	P08294	P08294	SODE_HUMAN	SOD3	Extracellular superoxide dismutase [Cu-Zn]
615	-2.04324375	16.51518646	0.036655903	Q86UD1	Q86UD1	OAF_HUMAN	OAF	Out at first protein homolog
224	-1.15093735	16.5252961	0.039553509	P04066	P04066	FUCO_HUMAN	FUCA1	Tissue alpha-L-fucosidase
47	-2.26227063	16.80139534	0.04012798	A0A1B0GUS4;P68036	A0A1B0GUS4;P68036	UB2L3_HUMAN;UB2L5_HUMAN	UBE2L3;UBE2L5	Ubiquitin-conjugating enzyme E2 L5
113	-0.74503966	30.69468205	0.040703718	P00738	P00738	HPT_HUMAN	HP	Haptoglobin
409	-1.25512733	16.25141617	0.041064607	P25787	P25787	PSA2_HUMAN	PSMA2	Proteasome subunit alpha type-2
454	-1.48199229	16.51187361	0.042086336	P36871	P36871	PGM1_HUMAN	PGM1	Phosphoglucomutase-1
577	-1.0373246	15.94310408	0.045241014	Q14247	Q14247	SRC8_HUMAN	CTTN	Src substrate cortactin
423	-1.19920686	16.21404237	0.045365278	P28065	P28065	PSB9_HUMAN	PSMB9	Proteasome subunit beta type-9
80	-2.03117456	16.34705239	0.046597515	O43865	O43865	SAHH2_HUMAN	AHCYL1	S-adenosylhomocysteine hydrolase-like protein 1
573	-1.15961105	16.70736716	0.046727838	Q13822	Q13822	ENPP2_HUMAN	ENPP2	Ectonucleotide pyrophosphatase/phosphodiesterase family member 2
656	-1.63279541	16.22842305	0.047234228	Q9BWD1	Q9BWD1	THIC_HUMAN	ACAT2	Acetyl-CoA acetyltransferase, cytosolic

Supplementary Table 12. GO terms enriched in the plasma of COVID-19 patients with vs without anti-Spike RBD antibodies detected.

ID	Description	pvalue	p.adjust	qvalue	Count	geneID
GO:0006910	phagocytosis, recognition	1.89E-23	2.3E-21	1.34E-21	12	IGHV1-18/IGHV1-24/IGHV1-69/IGHV1-69D/IGHM/IGHV3-30/IGHV3-30-5/IGHV4-39/IGHG2/IGHG4/IGHV3-15/IGHV4-28
GO:0006956	complement activation	7.14E-24	2.3E-21	1.34E-21	14	IGHV1-18/IGHV1-24/IGHV1-69/IGHV1-69D/IGHM/IGHV3-30/IGHV3-30-5/IGHV4-39/IGHG2/IGHG4/CD5L/IGKV1-16/IGHV3-15/IGHV4-28
GO:0050853	B cell receptor signaling pathway	2.08E-23	2.3E-21	1.34E-21	13	IGHV1-18/IGHV1-24/IGHV1-69/IGHV1-69D/IGHM/IGHV3-30/IGHV3-30-5/IGHV4-39/IGHG2/IGHG4/MNDA/IGHV3-15/IGHV4-28
GO:0006959	humoral immune response	1.47E-23	2.3E-21	1.34E-21	16	IGHV1-18/IGHV1-24/IGHV1-69/IGHV1-69D/IGHM/IGHV3-30/IGHV3-30-5/IGHV4-39/IGHG2/IGHG4/CD5L/IGKV1-16/IGHV3-15/LCN2/IGHV4-28/HRG
GO:0006958	complement activation, classical pathway	4.70E-23	4.1E-21	2.42E-21	13	IGHV1-18/IGHV1-24/IGHV1-69/IGHV1-69D/IGHM/IGHV3-30/IGHV3-30-5/IGHV4-39/IGHG2/IGHG4/IGKV1-16/IGHV3-15/IGHV4-28
GO:0002455	humoral immune response mediated by circulating immunoglobulin	1.60E-22	1.2E-20	6.87E-21	13	IGHV1-18/IGHV1-24/IGHV1-69/IGHV1-69D/IGHM/IGHV3-30/IGHV3-30-5/IGHV4-39/IGHG2/IGHG4/IGKV1-16/IGHV3-15/IGHV4-28
GO:0006911	phagocytosis, engulfment	1.40E-21	8.8E-20	5.17E-20	12	IGHV1-18/IGHV1-24/IGHV1-69/IGHV1-69D/IGHM/IGHV3-30/IGHV3-30-5/IGHV4-39/IGHG2/IGHG4/IGHV3-15/IGHV4-28
GO:0050864	regulation of B cell activation	2.48E-21	1.4E-19	7.99E-20	13	IGHV1-18/IGHV1-24/IGHV1-69/IGHV1-69D/IGHM/IGHV3-30/IGHV3-30-5/IGHV4-39/IGHG2/IGHG4/MNDA/IGHV3-15/IGHV4-28
GO:0099024	plasma membrane invagination	3.52E-21	1.7E-19	1.01E-19	12	IGHV1-18/IGHV1-24/IGHV1-69/IGHV1-69D/IGHM/IGHV3-30/IGHV3-30-5/IGHV4-39/IGHG2/IGHG4/IGHV3-15/IGHV4-28
GO:0010324	membrane invagination	7.54E-21	3.3E-19	1.94E-19	12	IGHV1-18/IGHV1-24/IGHV1-69/IGHV1-69D/IGHM/IGHV3-30/IGHV3-30-5/IGHV4-39/IGHG2/IGHG4/IGHV3-15/IGHV4-28
GO:0050871	positive regulation of B cell activation	1.41E-20	5.6E-19	3.31E-19	12	IGHV1-18/IGHV1-24/IGHV1-69/IGHV1-69D/IGHM/IGHV3-30/IGHV3-30-5/IGHV4-39/IGHG2/IGHG4/IGHV3-15/IGHV4-28
GO:0016064	immunoglobulin mediated immune response	2.37E-20	8.7E-19	5.10E-19	13	IGHV1-18/IGHV1-24/IGHV1-69/IGHV1-69D/IGHM/IGHV3-30/IGHV3-30-5/IGHV4-39/IGHG2/IGHG4/IGKV1-16/IGHV3-15/IGHV4-28
GO:0019724	B cell mediated immunity	2.84E-20	9.6E-19	5.64E-19	13	IGHV1-18/IGHV1-24/IGHV1-69/IGHV1-69D/IGHM/IGHV3-30/IGHV3-30-5/IGHV4-39/IGHG2/IGHG4/IGKV1-16/IGHV3-15/IGHV4-28
GO:0008037	cell recognition	2.33E-18	7.2E-17	4.24E-17	12	IGHV1-18/IGHV1-24/IGHV1-69/IGHV1-69D/IGHM/IGHV3-30/IGHV3-30-5/IGHV4-39/IGHG2/IGHG4/IGHV3-15/IGHV4-28
GO:0042113	B cell activation	2.47E-18	7.2E-17	4.24E-17	13	IGHV1-18/IGHV1-24/IGHV1-69/IGHV1-69D/IGHM/IGHV3-30/IGHV3-30-5/IGHV4-39/IGHG2/IGHG4/MNDA/IGHV3-15/IGHV4-28
GO:0050851	antigen receptor-mediated signaling pathway	3.17E-18	8.7E-17	5.12E-17	13	IGHV1-18/IGHV1-24/IGHV1-69/IGHV1-69D/IGHM/IGHV3-30/IGHV3-30-5/IGHV4-39/IGHG2/IGHG4/MNDA/IGHV3-15/IGHV4-28
GO:0042742	defense response to bacterium	5.60E-18	1.4E-16	8.50E-17	13	IGHV1-18/IGHV1-24/IGHV1-69/IGHV1-69D/IGHM/IGHV3-30/IGHV3-30-5/IGHV4-39/IGHG2/IGHG4/IGHV3-15/LCN2/IGHV4-28
GO:0002757	immune response-activating signal transduction	9.86E-18	2.3E-16	1.34E-16	14	IGHV1-18/IGHV1-24/IGHV1-69/IGHV1-69D/IGHM/IGHV3-30/IGHV3-30-5/IGHV4-39/IGHG2/IGHG4/MNDA/IGKV1-16/IGHV3-15/IGHV4-28
GO:0002429	immune response-activating cell surface receptor signaling pathway	9.86E-18	2.3E-16	1.34E-16	14	IGHV1-18/IGHV1-24/IGHV1-69/IGHV1-69D/IGHM/IGHV3-30/IGHV3-30-5/IGHV4-39/IGHG2/IGHG4/MNDA/IGKV1-16/IGHV3-15/IGHV4-28
GO:0002449	lymphocyte mediated immunity	1.30E-17	2.9E-16	1.68E-16	13	IGHV1-18/IGHV1-24/IGHV1-69/IGHV1-69D/IGHM/IGHV3-30/IGHV3-30-5/IGHV4-39/IGHG2/IGHG4/IGKV1-16/IGHV3-15/IGHV4-28
GO:0002460	adaptive immune response based on somatic recombination of immune receptors built from immunoglobulin superfamily domains	1.81E-17	3.8E-16	2.22E-16	13	IGHV1-18/IGHV1-24/IGHV1-69/IGHV1-69D/IGHM/IGHV3-30/IGHV3-30-5/IGHV4-39/IGHG2/IGHG4/IGKV1-16/IGHV3-15/IGHV4-28
GO:0006909	phagocytosis	2.41E-17	4.8E-16	2.83E-16	13	IGHV1-18/IGHV1-24/IGHV1-69/IGHV1-69D/IGHM/IGHV3-30/IGHV3-30-5/IGHV4-39/IGHG2/IGHG4/IGKV1-16/IGHV3-15/IGHV4-28
GO:0051251	positive regulation of lymphocyte activation	4.86E-16	9.3E-15	5.45E-15	12	IGHV1-18/IGHV1-24/IGHV1-69/IGHV1-69D/IGHM/IGHV3-30/IGHV3-30-5/IGHV4-39/IGHG2/IGHG4/IGHV3-15/IGHV4-28
GO:0051249	regulation of lymphocyte activation	8.41E-16	1.5E-14	9.04E-15	13	IGHV1-18/IGHV1-24/IGHV1-69/IGHV1-69D/IGHM/IGHV3-30/IGHV3-30-5/IGHV4-39/IGHG2/IGHG4/MNDA/IGHV3-15/IGHV4-28
GO:0002696	positive regulation of leukocyte activation	2.29E-15	4E-14	2.36E-14	12	IGHV1-18/IGHV1-24/IGHV1-69/IGHV1-69D/IGHM/IGHV3-30/IGHV3-30-5/IGHV4-39/IGHG2/IGHG4/IGHV3-15/IGHV4-28
GO:0050867	positive regulation of cell activation	3.53E-15	6E-14	3.50E-14	12	IGHV1-18/IGHV1-24/IGHV1-69/IGHV1-69D/IGHM/IGHV3-30/IGHV3-30-5/IGHV4-39/IGHG2/IGHG4/IGHV3-15/IGHV4-28

GO:0030449	regulation of complement activation	4.42E-11	7.2E-10	4.22E-10	7	IGHV1-69/IGHV3-30/IGHV4-39/IGHG2/IGHG4/CD5L/IGKV1-16
GO:0002920	regulation of humoral immune response	1.31E-10	2E-09	1.20E-09	7	IGHV1-69/IGHV3-30/IGHV4-39/IGHG2/IGHG4/CD5L/IGKV1-16
GO:0038096	Fc-gamma receptor signaling pathway involved in phagocytosis	1.03E-08	1.5E-07	8.89E-08	6	IGHV1-69/IGHV3-30/IGHV4-39/IGHG2/IGHG4/IGKV1-16
GO:0002433	immune response-regulating cell surface receptor signaling pathway involved in phagocytosis	1.03E-08	1.5E-07	8.89E-08	6	IGHV1-69/IGHV3-30/IGHV4-39/IGHG2/IGHG4/IGKV1-16
GO:0038094	Fc-gamma receptor signaling pathway	1.18E-08	1.7E-07	9.78E-08	6	IGHV1-69/IGHV3-30/IGHV4-39/IGHG2/IGHG4/IGKV1-16
GO:0002431	Fc receptor mediated stimulatory signaling pathway	1.33E-08	1.8E-07	1.07E-07	6	IGHV1-69/IGHV3-30/IGHV4-39/IGHG2/IGHG4/IGKV1-16
GO:0038093	Fc receptor signaling pathway	2.73E-07	3.6E-06	2.13E-06	6	IGHV1-69/IGHV3-30/IGHV4-39/IGHG2/IGHG4/IGKV1-16
GO:0002697	regulation of immune effector process	6.71E-07	8.6E-06	5.09E-06	7	IGHV1-69/IGHV3-30/IGHV4-39/IGHG2/IGHG4/CD5L/IGKV1-16
GO:0006898	receptor-mediated endocytosis	2.71E-05	0.00034	0.0002	5	IGHV1-69/IGHV3-30/IGHV4-39/APOC1/IGKV1-16
GO:0038095	Fc-epsilon receptor signaling pathway	4.17E-05	0.00051	0.0003	4	IGHV1-69/IGHV3-30/IGHV4-39/IGKV1-16
GO:0051918	negative regulation of fibrinolysis	5.93E-05	0.0007	0.00041	2	APOH/HRG
GO:1901678	iron coordination entity transport	8.69E-05	0.001	0.00059	2	LCN2/HRG
GO:0051917	regulation of fibrinolysis	0.00012	0.00134	0.00079	2	APOH/HRG
GO:0051004	regulation of lipoprotein lipase activity	0.00033	0.00353	0.00208	2	APOC1/APOH
GO:0019730	antimicrobial humoral response	0.00038	0.00399	0.00235	3	IGHM/LCN2/HRG
GO:0030194	positive regulation of blood coagulation	0.00042	0.00422	0.00248	2	APOH/HRG
GO:1900048	positive regulation of hemostasis	0.00042	0.00422	0.00248	2	APOH/HRG
GO:0050820	positive regulation of coagulation	0.00046	0.00445	0.00262	2	APOH/HRG
GO:0042730	fibrinolysis	0.00049	0.00468	0.00276	2	APOH/HRG
GO:1903661	positive regulation of complement-dependent cytotoxicity	0.00118	0.01075	0.00633	1	CD5L
GO:0010900	negative regulation of phosphatidylcholine catabolic process	0.00118	0.01075	0.00633	1	APOC1
GO:0006968	cellular defense response	0.0017	0.01516	0.00893	2	CD5L/MNDA
GO:0030195	negative regulation of blood coagulation	0.00176	0.01543	0.00908	2	APOH/HRG
GO:1900047	negative regulation of hemostasis	0.00183	0.0157	0.00924	2	APOH/HRG
GO:0050819	negative regulation of coagulation	0.00203	0.01713	0.01009	2	APOH/HRG
GO:0090303	positive regulation of wound healing	0.00233	0.01922	0.01132	2	APOH/HRG
GO:0006826	iron ion transport	0.00358	0.0266	0.01566	2	LCN2/HRG
GO:0015891	siderophore transport	0.00353	0.0266	0.01566	1	LCN2
GO:0061045	negative regulation of wound healing	0.00358	0.0266	0.01566	2	APOH/HRG
GO:0010899	regulation of phosphatidylcholine catabolic process	0.00353	0.0266	0.01566	1	APOC1
GO:0030193	regulation of blood coagulation	0.00387	0.02822	0.01662	2	APOH/HRG
GO:1900046	regulation of hemostasis	0.00396	0.02845	0.01675	2	APOH/HRG
GO:0050818	regulation of coagulation	0.00436	0.03079	0.01813	2	APOH/HRG
GO:0010916	negative regulation of very-low-density lipoprotein particle clearance	0.00471	0.0322	0.01896	1	APOC1
GO:1903035	negative regulation of response to wounding	0.00499	0.0336	0.01978	2	APOH/HRG
GO:0010596	negative regulation of endothelial cell migration	0.00577	0.03735	0.02199	2	APOH/HRG
GO:0060191	regulation of lipase activity	0.00588	0.03735	0.02199	2	APOC1/APOH
GO:0010915	regulation of very-low-density lipoprotein particle clearance	0.00588	0.03735	0.02199	1	APOC1
GO:0006641	triglyceride metabolic process	0.00672	0.04208	0.02478	2	APOC1/APOH
GO:0010593	negative regulation of lamellipodium assembly	0.00705	0.04289	0.02525	1	HRG
GO:0060696	regulation of phospholipid catabolic process	0.00705	0.04289	0.02525	1	APOC1
GO:0010633	negative regulation of epithelial cell migration	0.00788	0.04501	0.0265	2	APOH/HRG
GO:2001027	negative regulation of endothelial cell chemotaxis	0.00822	0.04501	0.0265	1	HRG
GO:0097577	sequestering of iron ion	0.00822	0.04501	0.0265	1	LCN2
GO:0051005	negative regulation of lipoprotein lipase activity	0.00822	0.04501	0.0265	1	APOC1
GO:1902744	negative regulation of lamellipodium organization	0.00939	0.04674	0.02752	1	HRG
GO:0015886	heme transport	0.00939	0.04674	0.02752	1	HRG
GO:0000041	transition metal ion transport	0.00927	0.04674	0.02752	2	LCN2/HRG
GO:0051715	cytolysis in other organism	0.00939	0.04674	0.02752	1	HRG
GO:1903659	regulation of complement-dependent cytotoxicity	0.00939	0.04674	0.02752	1	CD5L
GO:0150172	regulation of phosphatidylcholine metabolic process	0.00939	0.04674	0.02752	1	APOC1
GO:0034638	phosphatidylcholine catabolic process	0.00939	0.04674	0.02752	1	APOC1
GO:0006639	acylglycerol metabolic process	0.01	0.04865	0.02865	2	APOC1/APOH
GO:0006638	neutral lipid metabolic process	0.01015	0.04868	0.02866	2	APOC1/APOH
GO:0010873	positive regulation of cholesterol esterification	0.01056	0.04868	0.02866	1	APOC1
GO:0071830	triglyceride-rich lipoprotein particle clearance	0.01056	0.04868	0.02866	1	APOC1
GO:1903036	positive regulation of response to wounding	0.0034	0.0266	0.01566	2	APOH/HRG
GO:0034382	chylomicron remnant clearance	0.01056	0.04868	0.02866	1	APOC1
GO:0050900	leukocyte migration	0.00024	0.00264	0.00156	5	IGHV1-69/IGHM/IGHV3-30/IGHV4-39/IGKV1-16
GO:0002833	positive regulation of response to biotic stimulus	0.00299	0.02427	0.01429	3	XRCC6/MNDA/HRG
GO:0097680	double-strand break repair via classical nonhomologous end joining	0.00471	0.0322	0.01896	1	XRCC6
GO:2000504	positive regulation of blood vessel remodeling	0.00588	0.03735	0.02199	1	HRG
GO:0010046	response to mycotoxin	0.00822	0.04501	0.0265	1	LCN2
GO:0034196	acylglycerol transport	0.00822	0.04501	0.0265	1	APOH
GO:0034197	triglyceride transport	0.00822	0.04501	0.0265	1	APOH
GO:0071475	cellular hyperosmotic salinity response	0.00822	0.04501	0.0265	1	XRCC6
GO:0075713	establishment of integrated proviral latency	0.00939	0.04674	0.02752	1	XRCC6
GO:0002576	platelet degranulation	0.00985	0.04847	0.02854	2	APOH/HRG
GO:0019043	establishment of viral latency	0.01056	0.04868	0.02866	1	XRCC6
GO:0061041	regulation of wound healing	0.013	0.0532	0.03132	2	APOH/HRG
GO:0002831	regulation of response to biotic stimulus	0.01111	0.05068	0.02984	3	XRCC6/MNDA/HRG
GO:0097278	complement-dependent cytotoxicity	0.01172	0.05187	0.03054	1	CD5L
GO:0034447	very-low-density lipoprotein particle clearance	0.01172	0.05187	0.03054	1	APOC1
GO:0051006	positive regulation of lipoprotein lipase activity	0.01172	0.05187	0.03054	1	APOH
GO:0002218	activation of innate immune response	0.01201	0.05261	0.03097	2	XRCC6/MNDA
GO:0002836	positive regulation of response to tumor cell	0.01289	0.0532	0.03132	1	HRG

GO:0002839	positive regulation of immune response to tumor cell	0.01289	0.0532	0.03132	1	HRG
GO:0019042	viral latency	0.01289	0.0532	0.03132	1	XRCC6
GO:0033629	negative regulation of cell adhesion mediated by integrin	0.01289	0.0532	0.03132	1	HRG
GO:0034379	very-low-density lipoprotein particle assembly	0.01289	0.0532	0.03132	1	APOC1
GO:0061365	positive regulation of triglyceride lipase activity	0.01289	0.0532	0.03132	1	APOH
GO:0033700	phospholipid efflux	0.01405	0.05596	0.03295	1	APOC1
GO:0071472	cellular response to salt stress	0.01405	0.05596	0.03295	1	XRCC6
GO:0071481	cellular response to X-ray	0.01405	0.05596	0.03295	1	XRCC6
GO:0009635	response to herbicide	0.01522	0.05795	0.03412	1	LCN2
GO:0010872	regulation of cholesterol esterification	0.01522	0.05795	0.03412	1	APOC1
GO:0042538	hyperosmotic salinity response	0.01522	0.05795	0.03412	1	XRCC6
GO:0060312	regulation of blood vessel remodeling	0.01522	0.05795	0.03412	1	HRG
GO:1903726	negative regulation of phospholipid metabolic process	0.01522	0.05795	0.03412	1	APOC1
GO:1900747	negative regulation of vascular endothelial growth factor signaling pathway	0.01638	0.06028	0.03549	1	HRG
GO:0002834	regulation of response to tumor cell	0.01638	0.06028	0.03549	1	HRG
GO:0002837	regulation of immune response to tumor cell	0.01638	0.06028	0.03549	1	HRG
GO:0034392	negative regulation of smooth muscle cell apoptotic process	0.01638	0.06028	0.03549	1	APOH
GO:0001906	cell killing	0.01652	0.06031	0.03551	2	CD5L/HRG
GO:1902548	negative regulation of cellular response to vascular endothelial growth factor stimulus	0.01754	0.06245	0.03677	1	HRG
GO:0016525	negative regulation of angiogenesis	0.01746	0.06245	0.03677	2	APOH/HRG
GO:0071474	cellular hyperosmotic response	0.01754	0.06245	0.03677	1	XRCC6
GO:2000181	negative regulation of blood vessel morphogenesis	0.01784	0.06303	0.03711	2	APOH/HRG
GO:0043312	neutrophil degranulation	0.01859	0.06348	0.03738	3	XRCC6/MNDA/LCN2
GO:1903034	regulation of response to wounding	0.01862	0.06348	0.03738	2	APOH/HRG
GO:0030889	negative regulation of B cell proliferation	0.0187	0.06348	0.03738	1	MNDA
GO:0033033	negative regulation of myeloid cell apoptotic process	0.0187	0.06348	0.03738	1	APOH
GO:0051238	sequestering of metal ion	0.0187	0.06348	0.03738	1	LCN2
GO:0002283	neutrophil activation involved in immune response	0.01889	0.06366	0.03748	3	XRCC6/MNDA/LCN2
GO:0002418	immune response to tumor cell	0.01985	0.06405	0.03771	1	HRG
GO:0006266	DNA ligation	0.01985	0.06405	0.03771	1	XRCC6
GO:0034433	steroid esterification	0.01985	0.06405	0.03771	1	APOC1
GO:0034434	sterol esterification	0.01985	0.06405	0.03771	1	APOC1
GO:0034435	cholesterol esterification	0.01985	0.06405	0.03771	1	APOC1
GO:0042119	neutrophil activation	0.01993	0.06405	0.03771	3	XRCC6/MNDA/LCN2
GO:0002446	neutrophil mediated immunity	0.02003	0.06405	0.03771	3	XRCC6/MNDA/LCN2
GO:1901343	negative regulation of vasculature development	0.02021	0.06414	0.03777	2	APOH/HRG
GO:0007597	blood coagulation, intrinsic pathway(Single)	0.02101	0.06526	0.03843	1	APOH
GO:0034375	high-density lipoprotein particle remodeling	0.02101	0.06526	0.03843	1	APOC1
GO:0060192	negative regulation of lipase activity	0.02101	0.06526	0.03843	1	APOC1
GO:0002377	immunoglobulin production	0.02144	0.06613	0.03894	2	IGLV1-36/IGKV1-16
GO:0010985	negative regulation of lipoprotein particle clearance	0.02216	0.06789	0.03997	1	APOC1
GO:0035458	cellular response to interferon-beta	0.02332	0.07092	0.04176	1	MNDA
GO:0045717	negative regulation of fatty acid biosynthetic process	0.02562	0.07739	0.04557	1	APOC1
GO:0045089	positive regulation of innate immune response	0.02598	0.07795	0.0459	2	XRCC6/MNDA
GO:0002347	response to tumor cell	0.02677	0.07869	0.04633	1	HRG
GO:0050995	negative regulation of lipid catabolic process	0.02677	0.07869	0.04633	1	APOC1
GO:1900746	regulation of vascular endothelial growth factor signaling pathway	0.02677	0.07869	0.04633	1	HRG
GO:0046475	glycerophospholipid catabolic process	0.02792	0.08045	0.04737	1	APOC1
GO:1902547	regulation of cellular response to vascular endothelial growth factor stimulus	0.02792	0.08045	0.04737	1	HRG
GO:2001026	regulation of endothelial cell chemotaxis	0.02792	0.08045	0.04737	1	HRG
GO:0031639	plasminogen activation	0.02906	0.08266	0.04867	1	APOH
GO:0051894	positive regulation of focal adhesion assembly	0.02906	0.08266	0.04867	1	HRG
GO:0010594	regulation of endothelial cell migration	0.02945	0.08322	0.049	2	APOH/HRG
GO:0150117	positive regulation of cell-substrate junction organization	0.03021	0.08482	0.04994	1	HRG

Supplementary Table 13. GO terms enriched in the plasma of COVID-19 patients without vs with anti-Spike RBD antibodies detected.

ID	Description	pvalue	p.adjust	qvalue	Count	geneID
GO:1901750	leukotriene D4 biosynthetic process	4.17E-11	1.70E-08	1.30E-08	5	GGT1/GGT2/GGT3P/GGTLC2/GGTLC3
GO:1901748	leukotriene D4 metabolic process	4.17E-11	1.70E-08	1.30E-08	5	GGT1/GGT2/GGT3P/GGTLC2/GGTLC3
GO:0006751	glutathione catabolic process	4.17E-11	1.70E-08	1.30E-08	5	GGT1/GGT2/GGT3P/GGTLC2/GGTLC3
GO:0019370	leukotriene biosynthetic process	2.51E-09	7.65E-07	5.87E-07	5	GGT1/GGT2/GGT3P/GGTLC2/GGTLC3
GO:0042219	cellular modified amino acid catabolic process	1.89E-08	4.59E-06	3.52E-06	5	GGT1/GGT2/GGT3P/GGTLC2/GGTLC3
GO:0043171	peptide catabolic process	2.26E-08	4.59E-06	3.52E-06	5	GGT1/GGT2/GGT3P/GGTLC2/GGTLC3
GO:0006691	leukotriene metabolic process	2.69E-08	4.68E-06	3.59E-06	5	GGT1/GGT2/GGT3P/GGTLC2/GGTLC3
GO:0051187	cofactor catabolic process	3.19E-08	4.86E-06	3.72E-06	6	GGT1/GGT2/GGT3P/GGTLC2/GGTLC3/HP
GO:0031179	peptide modification	2.28E-07	3.09E-05	2.37E-05	3	GGT1/GGT2/GGT3P
GO:0046456	icosanoid biosynthetic process	5.22E-07	5.78E-05	4.44E-05	5	GGT1/GGT2/GGT3P/GGTLC2/GGTLC3
GO:0044273	sulfur compound catabolic process	5.22E-07	5.78E-05	4.44E-05	5	GGT1/GGT2/GGT3P/GGTLC2/GGTLC3
GO:0006749	glutathione metabolic process	5.72E-07	5.81E-05	4.45E-05	5	GGT1/GGT2/GGT3P/GGTLC2/GGTLC3
GO:0006575	cellular modified amino acid metabolic process	1.11E-06	0.0001	8.01E-05	7	CKM/GGT1/GGT2/GGT3P/GGTLC2/GGTLC3/ALDH9A1
GO:1901570	fatty acid derivative biosynthetic process	9.75E-06	0.00085	0.00065	5	GGT1/GGT2/GGT3P/GGTLC2/GGTLC3
GO:0002446	neutrophil mediated immunity	1.18E-05	0.00096	0.00074	9	WDR1/RAB6A/PTX3/CPPED1/DIAPH1/FUCA1/HP/PSMA2/PGM1
GO:0042398	cellular modified amino acid biosynthetic process	1.30E-05	0.00099	0.00076	4	CKM/GGT1/GGT3P/ALDH9A1
GO:0006690	icosanoid metabolic process	1.94E-05	0.00139	0.00106	5	GGT1/GGT2/GGT3P/GGTLC2/GGTLC3
GO:0051186	cofactor metabolic process	4.15E-05	0.00281	0.00215	8	GGT1/GGT2/GGT3P/GGTLC2/GGTLC3/SPTA1/HP/AHCYL1
GO:0043312	neutrophil degranulation	7.13E-05	0.00454	0.00348	8	RAB6A/PTX3/CPPED1/DIAPH1/FUCA1/HP/PSMA2/PGM1
GO:0002283	neutrophil activation involved in immune response	7.44E-05	0.00454	0.00348	8	RAB6A/PTX3/CPPED1/DIAPH1/FUCA1/HP/PSMA2/PGM1
GO:0042119	neutrophil activation	8.57E-05	0.00498	0.00382	8	RAB6A/PTX3/CPPED1/DIAPH1/FUCA1/HP/PSMA2/PGM1
GO:1901568	fatty acid derivative metabolic process	0.00012	0.00634	0.00486	5	GGT1/GGT2/GGT3P/GGTLC2/GGTLC3
GO:0044272	sulfur compound biosynthetic process	0.00023	0.01165	0.00894	5	GGT1/GGT2/GGT3P/GGTLC2/GGTLC3
GO:0044331	cell-cell adhesion mediated by cadherin	0.00028	0.01358	0.01041	3	CDH11/CDH13/CDH2
GO:0016339	calcium-dependent cell-cell adhesion via plasma membrane cell adhesion molecules	0.00036	0.01692	0.01297	3	CDH11/CDH13/CDH2
GO:0007043	cell-cell junction assembly	0.00053	0.02162	0.01657	4	CDH11/WDR1/CDH13/CDH2
GO:0006790	sulfur compound metabolic process	0.00069	0.02724	0.02089	6	GGT1/GGT2/GGT3P/GGTLC2/GGTLC3/AHCYL1
GO:0034332	adherens junction organization	0.00088	0.03353	0.02571	3	CDH11/CDH13/CDH2
GO:0006750	glutathione biosynthetic process	0.00109	0.04012	0.03076	2	GGT1/GGT3P
GO:0034329	cell junction assembly	0.00113	0.04059	0.03112	6	CDH11/WDR1/CDH13/CDH2/DUSP3/CTTN
GO:0019184	nonribosomal peptide biosynthetic process	0.00136	0.04607	0.03533	2	GGT1/GGT3P
GO:0006891	intra-Golgi vesicle-mediated transport	0.00012	0.00634	0.00486	3	COPA/RAB6A/RAB6B
GO:0022604	regulation of cell morphogenesis	0.00047	0.021	0.01611	7	WDR1/SEMA3A/CDH2/SPTA1/DIAPH1/CTTN/ENPP2
GO:0002576	platelet degranulation	0.00051	0.02162	0.01657	4	WDR1/ITIH3/ISLR/RAB27B
GO:0008228	opsonization	0.00053	0.02162	0.01657	2	C4B/PTX3
GO:0032482	Rab protein signal transduction	0.00133	0.04607	0.03533	3	RAB6A/RAB6B/RAB27B
GO:0050852	T cell receptor signaling pathway	0.02066	0.13413	0.10285	3	DUSP3/PSMA2/PSMB9
GO:0050868	negative regulation of T cell activation	0.04169	0.1734	0.13297	2	DUSP3/FGL1
GO:0050863	regulation of T cell activation	0.0624	0.20365	0.15616	3	DUSP3/SPTA1/FGL1
GO:0050856	regulation of T cell receptor signaling pathway	0.10692	0.25113	0.19257	1	DUSP3
GO:0042110	T cell activation	0.15047	0.28437	0.21806	3	DUSP3/SPTA1/FGL1
GO:0042102	positive regulation of T cell proliferation	0.24109	0.36828	0.2824	1	SPTA1
GO:0042129	regulation of T cell proliferation	0.36476	0.45233	0.34685	1	SPTA1
GO:0042098	T cell proliferation	0.41468	0.48652	0.37307	1	SPTA1
GO:0050870	positive regulation of T cell activation	0.44472	0.51288	0.39328	1	SPTA1

Supplementary Table 14. GO terms enriched in vaccinated individuals with/without antibodies vs Health group

GeneSet	Pvalue (Vaccinated individuals without antibodies vs Health)	Pvalue (Vaccinated individuals with antibodies vs Health)
POSITIVE_REGULATION_OF_CD4_POSITIVE_ALPHA_BETA_T_CELL_ACTIVATION	0.042	>0.05
ALPHA_BETA_T_CELL_ACTIVATION	0.048	>0.05
NEGATIVE_REGULATION_OF_ALPHA_BETA_T_CELL_ACTIVATION	0.004	0.031
NEGATIVE_REGULATION_OF_CD4_POSITIVE_ALPHA_BETA_T_CELL_ACTIVATION	0.010	0.020
ALPHA_BETA_T_CELL_PROLIFERATION	0.020	0.014
NEGATIVE_REGULATION_OF_CD4_POSITIVE_ALPHA_BETA_T_CELL_DIFFERENTIATION	0.028	0.051

Note: Health:non-infected, non-vaccinated groups

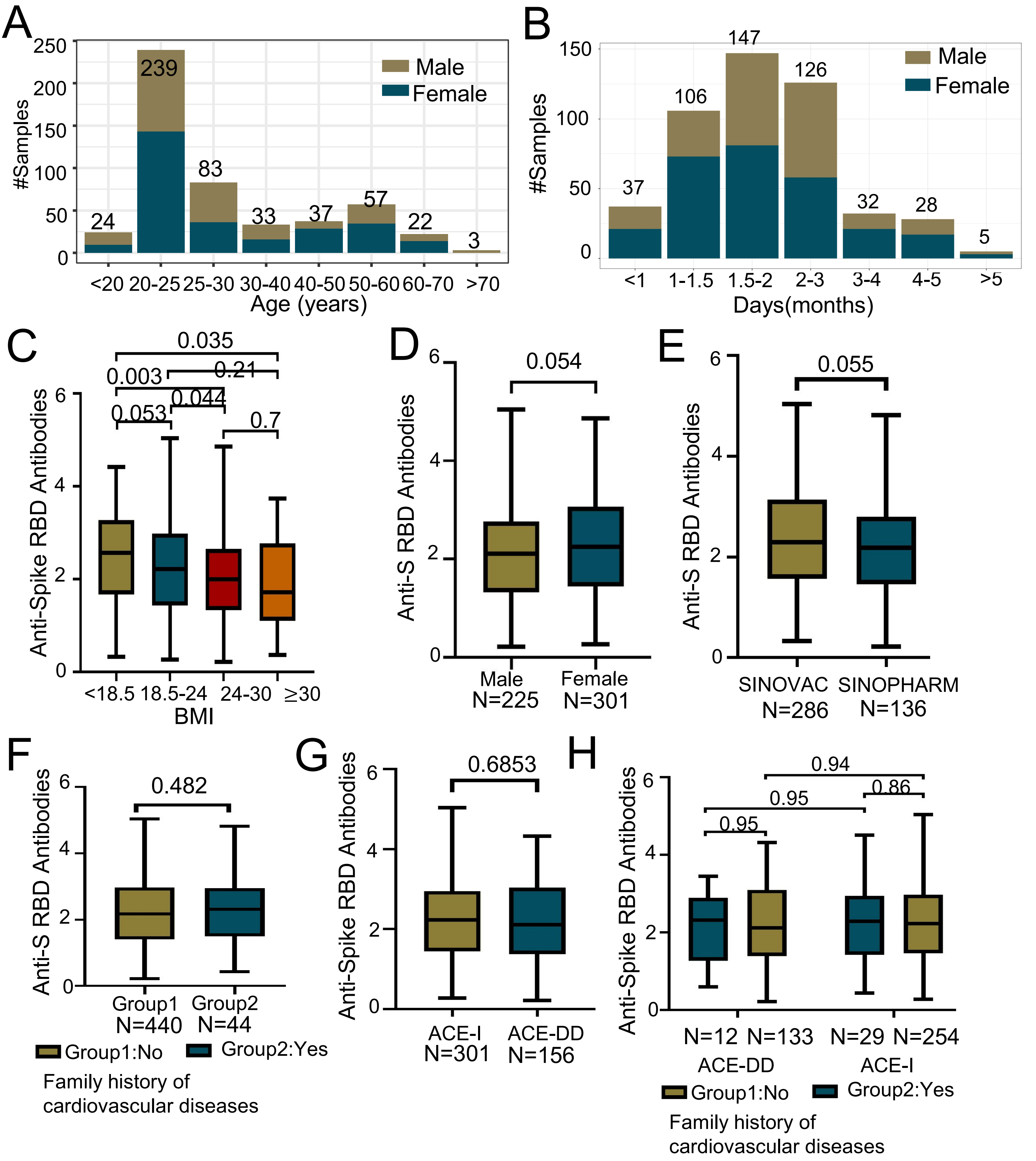

Supplementary Figure 1. The characteristics of vaccinated subjects. A, The distribution of vaccinated subjects across age. B, The distribution of vaccinated subjects over days interval between antibody testing and vaccination. C,D,E,F,G,H, The anti-Spike RBD antibody levels across different BMI groups (C), gender (D), different brands (E), with or without family history of cardiovascular diseases (F), ACE genotype (G) and the different combination of family history of cardiovascular diseases and ACE genotype, respectively. N represents the number of patients.

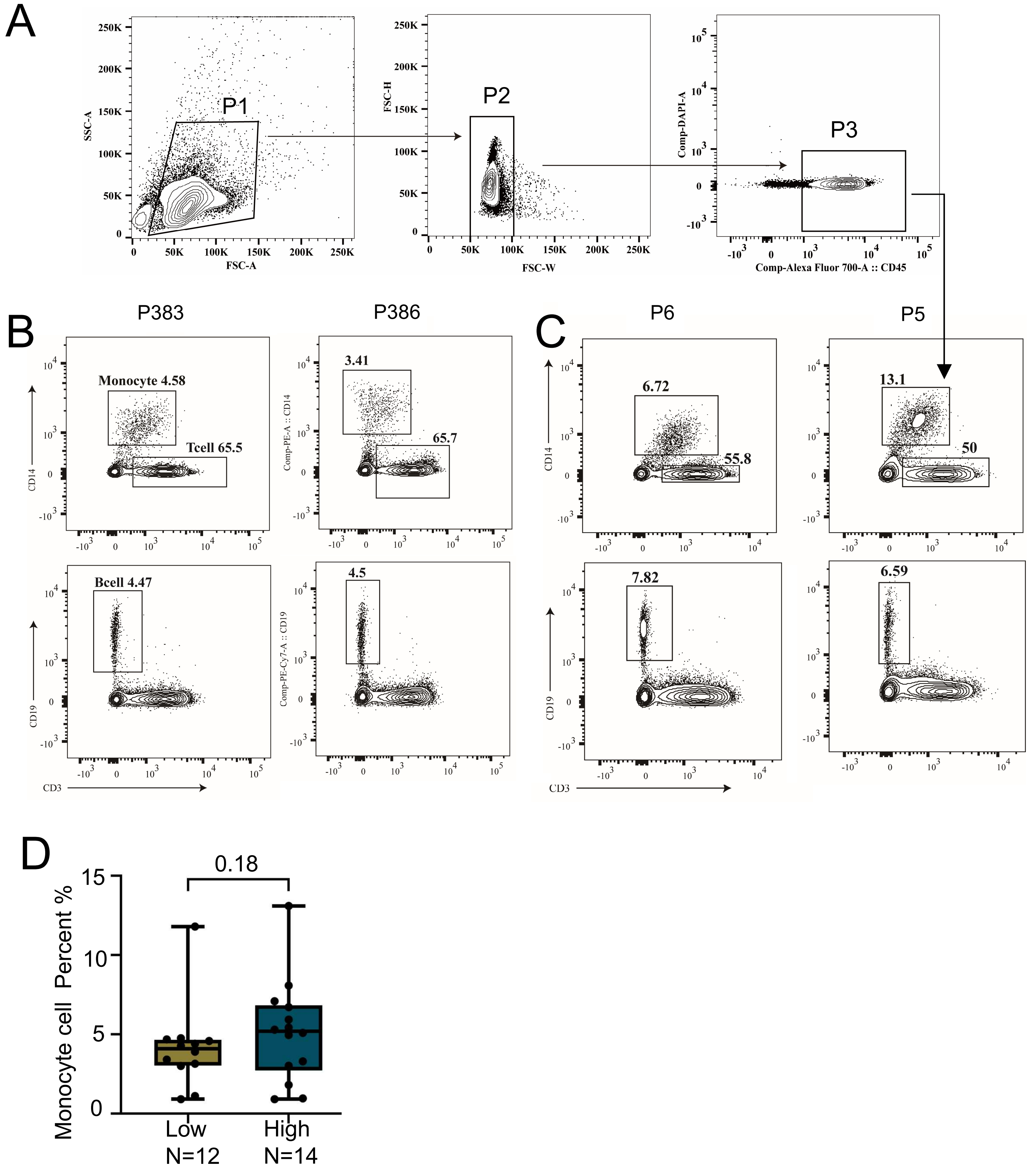

Supplementary Figure 2. Flow cytometry analysis result for 26 samples PBMC. A, Flow cytometry plots showing sorting strategy for T cells,B cells, monocyte.Plots were generated using FlowJo software. (B-C). Frequency of T cells, B cells, monocyte cells among expressing in two vaccinated subjects without anti-S RBD antibodies (B) and two subjects with anti-S RBD antibodies (C). The top panel shows the expression pattern of CD3 and CD14 on T cell and monocyte cell. The bottom panel shows the expression pattern of CD3 and CD19 on B cell.The value in the figure id the Frequency of cell(%). D, Boxplot analysis showing the frequency monocyte cell of $CD45^{+}$ cells between 14 vaccinated subjects with antibodies and 12 vaccinated ones without antibodies. P value was calculated by Mann Whitney U test. N represents the number of patients.
